# Observed poleward heat transport by mesoscale eddies in the deep Southern Ocean

**DOI:** 10.1038/s41467-026-76674-2

**Published:** 2026-08-12

**Authors:** Tongya Liu, Xiaoming Zhai, Qingyou He, Andrew McC. Hogg, Dake Chen

**Affiliations:** 1 State Key Laboratory of Satellite Ocean Environment Dynamics, Second Institute of Oceanography, Ministry of Natural Resources, Hangzhou, China; 2 School of Environmental Sciences, University of East Anglia, Norwich, UK; 3 State Key Laboratory of Tropical Oceanography, South China Sea Institute of Oceanology, Chinese Academy of Sciences, Guangzhou, China; 4 Australia’s Climate Simulator (ACCESS-NRI) and Research School of Earth Sciences, Australian National University, Canberra, ACT Australia; 5 Southern Marine Science and Engineering Guangdong Laboratory (Zhuhai), Zhuhai, China

**Keywords:** Physical oceanography, Ocean sciences

## Abstract

Mesoscale eddies in the Southern Ocean extend from the surface to the deep ocean and contribute to regional and global ocean heat budgets. However, sparse in situ observations have limited estimates of eddy meridional heat transport (EMHT) to the near surface, leaving deep-ocean processes poorly understood. Here we quantify deep eddy kinetic energy (EKE) and EMHT using Argo observations. We find that deep EMHT reaches at least the same order of magnitude as surface transport, despite deep EKE being only one-fifth of surface levels. Float observations and sensitivity experiments reveal that, relative to mean-flow transport alone, deep EMHT extends the meridional movement of subtropical warm waters towards Antarctica by over ten degrees of latitude. About 60% of this deep transport originates from the Indian Ocean sector. These findings provide observational evidence that mesoscale eddies can modulate deep Southern Ocean heat redistribution, with potential implications for Antarctic climate.

## Abstract (web summary)

Two decades of Argo observations reveal that mesoscale eddies redistribute heat in the deep Southern Ocean at rates comparable in magnitude to surface eddy heat transport, with about 60% of this deep transport originating from the Indian Ocean sector.

## Introduction

The Southern Ocean is a vital component of the Earth’s climate system, playing a pivotal role in global heat and carbon cycles^1–3^. Central to this role is the eastward-flowing Antarctic Circumpolar Current (ACC), which forms the ocean’s only continuous circumglobal pathway^4^. Driven by prevailing westerly winds and buoyancy forcing, the ACC provides a robust conduit for zonal interbasin exchange, yet its strong fronts and jets act as a kinematic barrier limiting meridional transport^4–6^. Concurrently, strongly sloping density surfaces associated with the ACC store available potential energy that is released by baroclinic instability, generating vigorous mesoscale eddies^6–8^. These eddies often extend to depths greater than 1500 m^9^ and facilitate the redistribution of heat across the ACC^10,11^, thereby influencing the meridional overturning circulation and the distribution of Antarctic sea ice^12–15^. As such, a quantitative understanding of eddy meridional heat transport (EMHT) in the Southern Ocean is essential for predicting global heat redistribution and climate variability.

The importance of EMHT in the Southern Ocean heat budget has been recognized for decades. Pioneering investigations primarily utilized hydrographic inversions^16^, localized current meters (e.g., in the Drake Passage)^17,18^, and sparse early Lagrangian floats^19^ to infer heat transport across the ACC. To achieve large-scale coverage, subsequent estimates have heavily relied on sea surface satellite observations or numerical simulations, yielding a consensus that the EMHT in the Southern Ocean is predominantly poleward with a peak around 40°S^20–23^. However, these broad-scale estimates typically provide depth-integrated values, thereby obscuring the vertical structure of EMHT. Meanwhile, studies of isopycnal mixing have shown that meridional mixing is strongly suppressed near the sea surface due to zonal jets, but becomes highly efficient at greater depths^24–29^. This eddy-enhanced mixing underscores the potential role of mesoscale eddies in deep-ocean heat transport. Yet, direct observational constraints on EMHT in the deep Southern Ocean remain extremely limited.

A further complication is that deep EMHT is expected to vary strongly among ocean basins. Its magnitude depends jointly on eddy activity and the background temperature field, both of which exhibit significant regional variability. The spatial heterogeneity of eddy activity is evident along the ACC pathway, such as the Agulhas Return Current region and complex topography areas, where eddy kinetic energy (EKE) can be 2–3 times higher than in other regions^9,30–33^. Moreover, background temperature differs markedly among the three ocean basins, with the Indian sector containing substantially warmer waters than the Pacific and Atlantic sectors^34,35^ (Supplementary Fig. 1). These pronounced regional contrasts suggest that mesoscale eddies may play distinct roles in interbasin heat exchange. Previous model- and satellite-based studies have found that the Indian sector serves as the main pathway for the poleward heat transport^11,19,36^. However, the contribution of the deep EMHT originating from each major ocean basin remains unquantified from observations.

In this study, we utilize velocity and temperature derived from a two-decade accumulation of Argo floats to directly quantify the intensity of EKE and EMHT in the deep Southern Ocean, and unveil the pathways and efficiency of the deep EMHT originating from different ocean basins. This work provides direct observational evidence for the deep-ocean heat transport. Our results underscore the dominant role of mesoscale eddies in governing the heat budget in the deep Southern Ocean, with profound implications for the climate variability in high-latitude regions.

## Results

### Deep eddy kinetic energy and eddy meridional heat transport

Argo trajectory-derived velocities help overcome the scarcity of long-term direct velocity measurements in the deep Southern Ocean, but they are inferred from float displacements at the parking depth of 1000 m over approximately 10-day intervals (see “Argo data” in “Methods”). We therefore first evaluate whether these discrete velocities can reliably capture dominant EKE at 1000 m in two ways. First, spectral analyses based on velocity fields from the Biogeochemical Southern Ocean State Estimate (B-SOSE; an eddy-permitting ocean reanalysis with 1/6° horizontal resolution) and four independent deep mooring records show that motions with periods shorter than 10 days account for only a small fraction of the total kinetic energy at 1000 m, ranging from 1.2% in B-SOSE to less than 8% in the mooring records (Supplementary Fig. 2), suggesting that Argo velocities retain most of the velocity variability at this depth. Second, we advect synthetic particles with B-SOSE velocity fields and reconstruct Argo-like discrete velocities from particle displacements (see “Validation of Argo-based estimates” in “Methods”). The reconstructed velocities well reproduce the spatial patterns and magnitudes of mean kinetic energy (MKE) and EKE from gridded B-SOSE, with a slight underestimation of EKE between 45°S and 60°S (Supplementary Fig. 3).

**Fig. 1:**
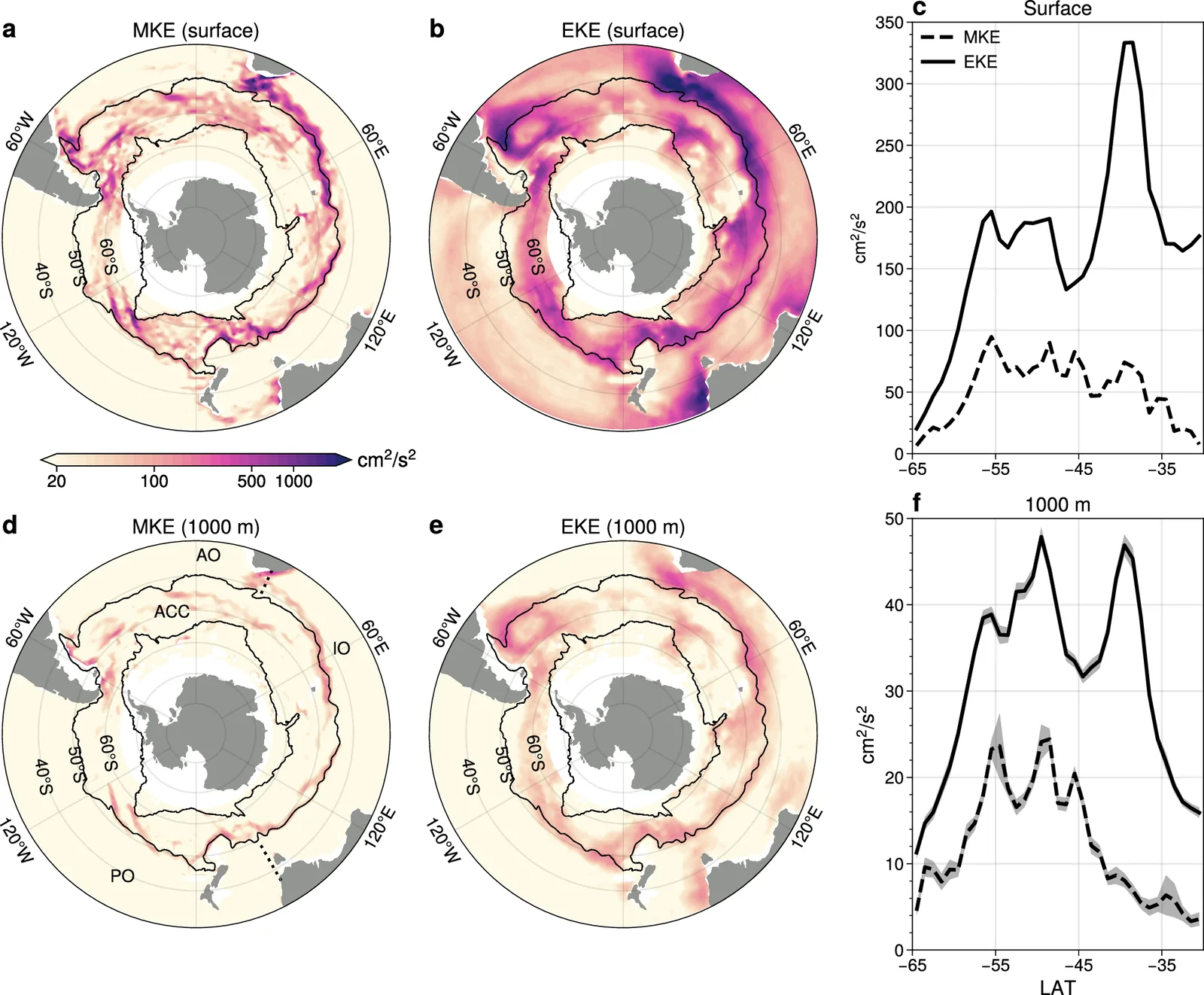
Kinetic energy distribution in the Southern Ocean. **a** Surface mean kinetic energy (MKE). **b** Surface eddy kinetic energy (EKE). **c** Zonal-mean kinetic energy at the surface. The black dashed and black solid lines denote the zonal-mean MKE and EKE, respectively. **d** MKE at 1000 m. **e** EKE at 1000 m. **f** Same as **c**, but for 1000 m. Shading in (**f**) indicates twice the standard deviation estimated from 1000 bootstrap resamplings. Black lines in (**a**, **b**, **d**, **e**) indicate the northern and southern boundaries of the Antarctic Circumpolar Current.

Given this validation, we use the Argo-derived velocities to construct observation-based maps of MKE and EKE at 1000 m depth in the Southern Ocean (Fig. 1; see “EKE” in “Methods”). The deep EKE distribution reveals pronounced hotspots along the northern boundary of the ACC and near major topographic features such as the Drake Passage, where values exceed 200 cm²/s² (Fig. 1e). These regions are characterized by enhanced baroclinic instability and complex interactions between the ACC and bathymetry, driving persistent eddy generation^9,37^. Deep EKE is spatially similar to surface patterns, but approximately an order of magnitude weaker around the ACC’s northern boundary (~40°S). In addition, the MKE distribution reveals that mean flow strength at 1000 m exhibits a more rapid reduction with depth in this region, indicating a higher fractional contribution of EKE to total kinetic energy at 1000 m (Fig. 1c, f). This observation implies that eddies may exert a more dominant role in meridional heat transport in the deep ocean compared to their role near the surface due to diminished zonal flow suppression.

The observed spatial heterogeneity in deep EKE can also be reflected by regional variations in the intensity of mesoscale eddies. Here, the Southern Ocean is divided into four regions, namely the ACC, Pacific, Indian, and Atlantic Ocean sectors, to reveal pronounced differences in temperature anomalies within mesoscale eddies (as a thermodynamic proxy for eddy intensity) through composite analysis based on Argo profiles (Supplementary Fig. 4). Eddies in the ACC, Pacific, and Atlantic Ocean sectors are surface-intensified, with the strongest temperature anomalies confined to the upper 500 m. In addition, the Pacific Ocean sector displays the weakest eddy intensity, likely due to the absence of strong mean currents to supply energy. By contrast, eddies in the Indian Ocean sector exhibit subsurface-intensified temperature anomalies, with peak magnitudes centered at 700–1000 m depth. These features result in temperature anomalies in the deep Indian Ocean exceeding 1.5 °C, which are significantly stronger than those in the other three sectors. This pattern highlights the potential significance of the Indian Ocean sector in modulating EMHT in the deep Southern Ocean.

Since Argo floats can reflect velocity perturbations and measure temperature anomalies in the deep Southern Ocean, we can diagnose the EMHT at specific locations. Before applying this approach to observations, we test whether the particle-based method can recover deep EMHT in B-SOSE. It is found that the particle-based estimates derived from B-SOSE reproduce the spatial pattern of deep EMHT well, with a spatial correlation of 0.8, but underestimate the zonally integrated transport, reaching about 70% of the gridded B-SOSE estimate between 40°S and 60°S (Supplementary Fig. 5). This low bias likely has two sources. The 10-day displacement velocities smooth instantaneous meridional velocity perturbations, and the temperature is averaged along the trajectory to remain consistent with the information available in the Argo dataset. Although this approach underestimates the magnitude of deep EMHT, this validation supports applying it to climatological estimates from Argo observations.

**Fig. 2:**
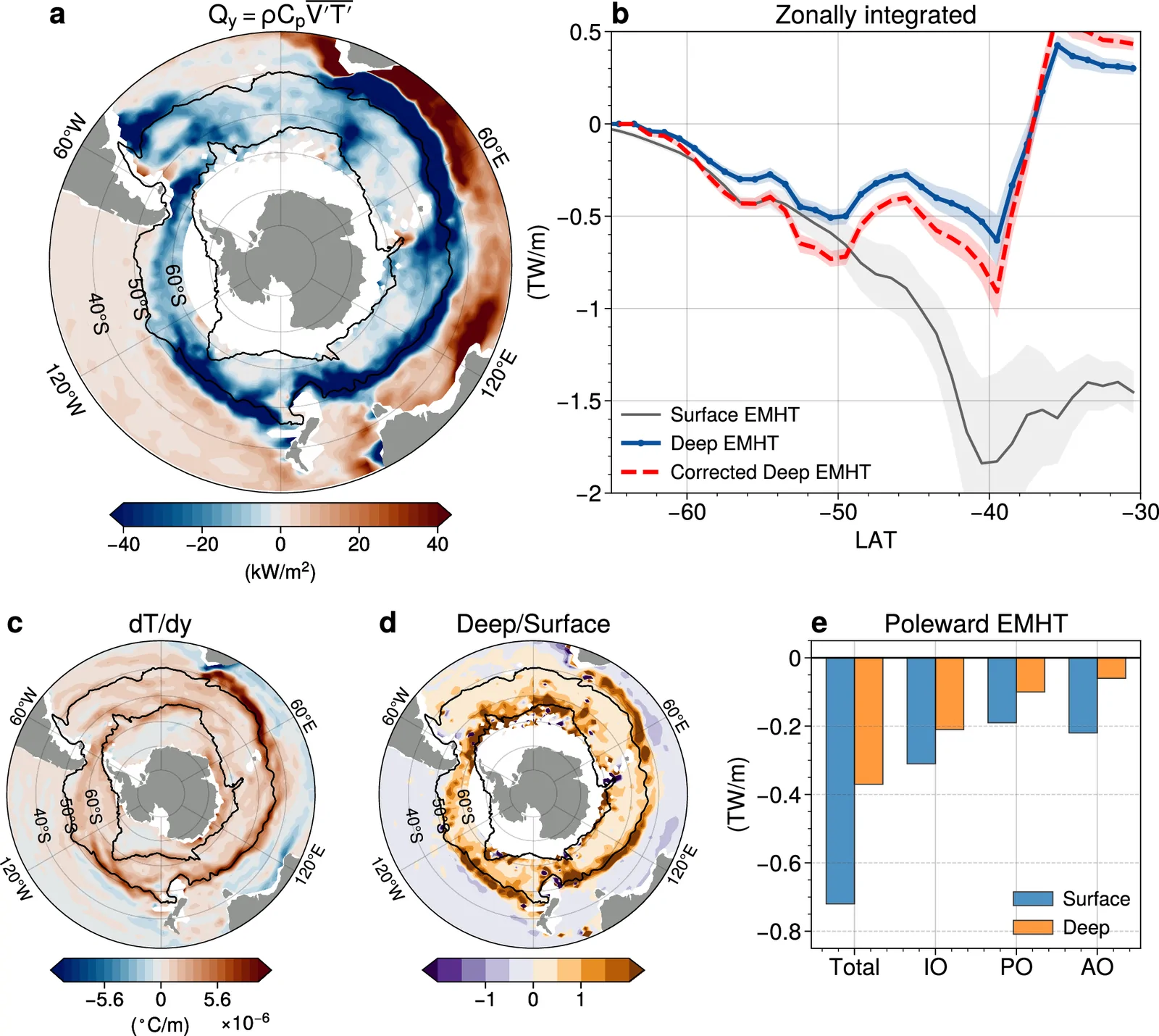
Eddy meridional heat transport (EMHT) in the deep Southern Ocean. **a** Spatial distribution of EMHT at 1000 m depth (color shading; kW/m²). Black contours mark the northern and southern boundaries of the Antarctic Circumpolar Current. **b** Zonally integrated EMHT at 1000 m depth versus latitude (blue line; TW/m). Shading denotes twice the standard deviation from 1000 bootstrap resamples. The red line shows the bias-corrected estimate. The black line shows surface EMHT from gridded satellite-based fields for comparison, with gray shading indicating the standard deviation from monthly climatology. **c** Spatial distribution of the meridional temperature gradient at 1000 m. **d** Ratio of EMHT at 1000 m depth to surface EMHT. **e** Basin-scale comparison of poleward EMHT at 1000 m and at the surface.

Here, we present the first Argo-based map of EMHT in the deep Southern Ocean (see “EMHT” in “Methods”). The EMHT spatial pattern correlates strongly with EKE distribution, peaking near the northern boundary of the ACC (Fig. 2a). Unlike surface EMHT, which predominantly transports heat poleward across the entire Southern Ocean^22,23^, EMHT at 1000 m displays equatorward anomalies on the northern side of the ACC, particularly north of the Agulhas Current, southwest of Australia, and around the East Australian Current. This spatial feature likely arises from the background temperature gradients (Fig. 2c). The Agulhas Current and the East Australian Current advect warm subtropical waters poleward, generating warm tongues along their extension jets (Supplementary Fig. 1). Anticyclonic eddies tend to detach from the northern edges of these extensions and transport warm water equatorward, while cyclonic eddies generated along the southern edges dominate poleward transport. The resultant dipole structure reflects heat divergence along the ACC’s northern flank at 1000 m depth, suggesting a potential influence of western boundary current systems on deep EMHT.

The zonally integrated EMHT at 1000 m peaks near 40°S, with a maximum poleward flux of −0.63 ± 0.06 TW/m, and a mean transport of −0.35 ± 0.02 TW/m between 40°S and 60°S (Fig. 2b). In comparison, the mean surface EMHT over the same latitude band is approximately −0.71 ± 0.13 TW/m. These results demonstrate that EMHT at 1000 m reaches half the surface magnitude, despite deep EKE being only one-fifth of surface levels (Fig. 1c, f). Although the zonally integrated EMHT at 1000 m is weaker than the surface estimate, its regional magnitude can be comparable to, or locally exceed, the surface EMHT, particularly along the northern boundary of the ACC in the Indian sector (Fig. 2d). This vertical structure provides observational support for the theoretical framework of eddy mixing suppression across strong jets^24,26,38^, in which strong mean flows such as the ACC suppress cross-jet eddy mixing near the surface, while this suppression weakens at depth as mean flows slow. In addition, large subsurface temperature anomalies associated with mesoscale eddies enhance EMHT at 1000 m depth, with this effect being especially pronounced in the Indian Ocean sector (Supplementary Fig. 4). It is also notable that, despite occupying only about one-third of the circumpolar longitude range, the Indian sector accounts for nearly 60% of EMHT at 1000 m, compared with 42% at the surface.

Moreover, the Argo-based calculation likely provides a conservative estimate of deep EMHT. In the B-SOSE validation, the particle-based estimates recover only about 70% of the zonally integrated EMHT between 40°S and 60°S. If a similar low bias applies to the Argo-based estimate, the mean EMHT at 1000 m would be approximately −0.50 ± 0.03 TW/m over this latitude band, exceeding half of the surface EMHT. However, B-SOSE itself does not reproduce such a strong deep EMHT, likely because the eddy thermal structure at 1000 m is weaker in the model than in the observations. Eddy-composite analysis shows that, in the Indian sector, the temperature anomalies of both cyclonic and anticyclonic eddies in B-SOSE are only about half of those derived from Argo profiles. This discrepancy suggests that accurately representing the deep thermal structure of mesoscale eddies is essential for simulating deep EMHT in the Southern Ocean.

### Heat exchange across different basins

In the deep Southern Ocean, mesoscale eddy features and background temperature fields exhibit pronounced regional heterogeneity, so it is important to examine the contribution of eddy heat exchange across different basins. As Lagrangian platforms, surface drifters and Argo floats track the movement of water parcels at the sea surface and in the deep layer (1000 m), respectively, effectively capturing the spatial extent of water mass under the combined influence of eddies and mean flow. We analyze the trajectories of Argo floats from four regions over a 1000-day period starting from January 1, 2013, and compare them with surface drifter paths.

**Fig. 3:**
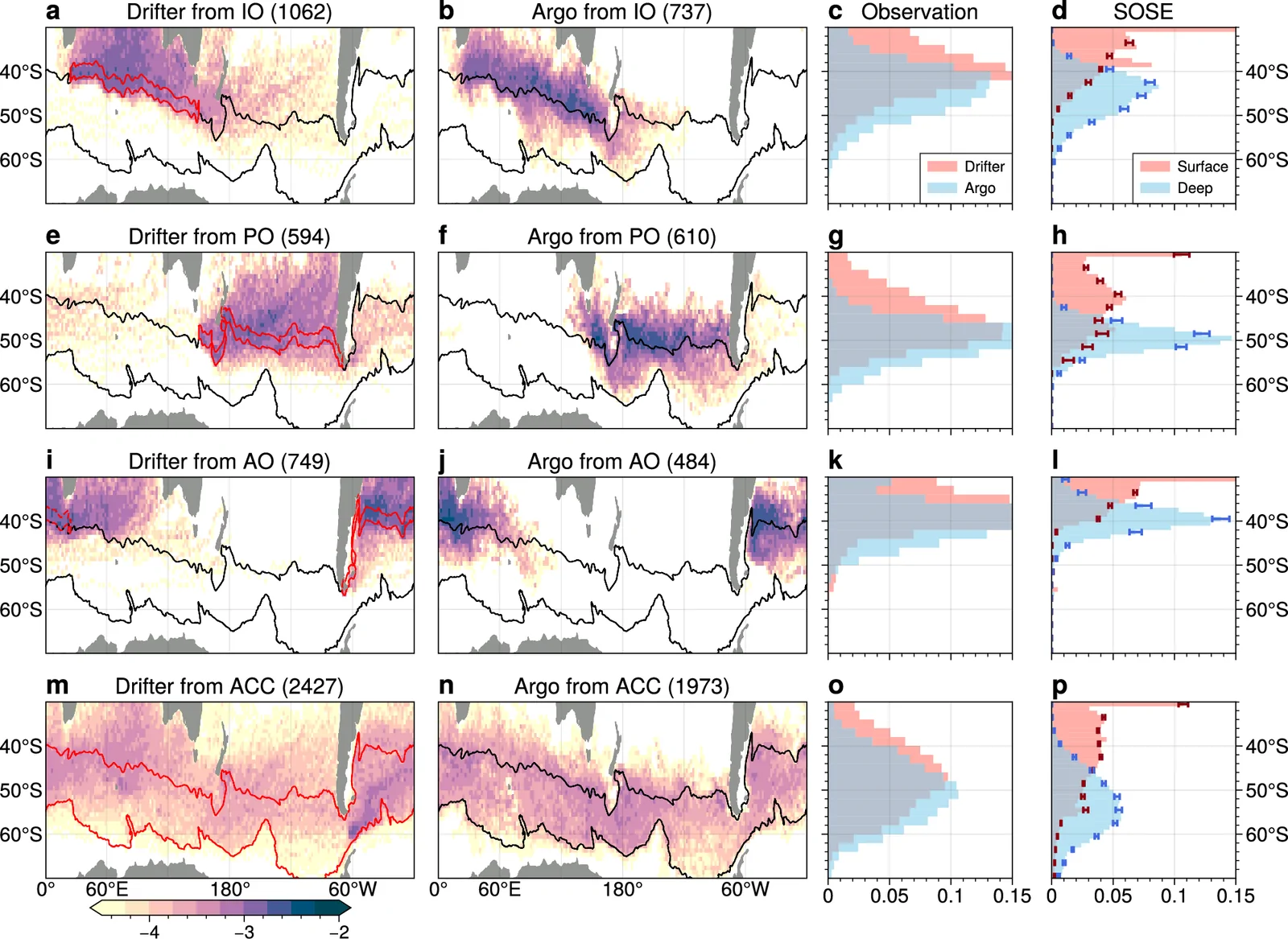
Spatial and latitudinal probability distributions of surface drifters, Argo floats, and Biogeochemical Southern Ocean State Estimate (B-SOSE) particles in the Southern Ocean. Spatial probability distributions of surface drifters (**a**) and Argo floats (**b**) from the Indian Ocean sector over 1000 days, along with the latitudinal probability distributions of the observations (**c**) and B-SOSE particles (**d**). **e**–**h** from the Pacific Ocean sector. **i**–**l** from the Atlantic Ocean sector. **m**–**p** from the entire Antarctic Circumpolar Current (ACC) region. Numbers in parentheses indicate total counts for each region. Red lines in the first column outline the initial positions of all observations and model particles. Color shading indicates log10 probability density. Black lines mark the northern and southern boundaries of the ACC. Error bars in the fourth column represent the standard deviation of 50 random subsamples.

In the Indian Ocean sector, 1062 surface drifter and 737 Argo float trajectories reveal divergent kinematic signatures (Fig. 3a–c). Surface drifters exhibit intensified zonal motion under the influence of the ACC jet, with some drifters completing full circumpolar loops. In contrast, Argo floats display weaker zonal displacement, extending only to approximately 120°W, but show pronounced meridional movement, especially in two bathymetrically complex regions (Supplementary Fig. 6) that coincide with areas of high EKE. The trajectory probability distributions show that 29% of Argo trajectory positions lie poleward of the ACC northern boundary, compared with 8% for surface drifters, indicating greater penetration of deep water than surface water into higher latitudes. Similar patterns are also observed in the other three regions. We further evaluate this pattern using particles advected by B-SOSE velocity fields, which show a pattern similar to observations but with stronger surface-deep separation, likely because the synthetic particles provide complete trajectories over the prescribed 1000-day period, whereas some observed trajectories, especially surface-drifter trajectories, do not persist for that long (Supplementary Fig. 7).

The trajectories of Argo floats are jointly influenced by mean flow and eddy motions. To quantitatively disentangle their respective contributions to tracer transport in the deep Southern Ocean, we conduct a series of 2D offline tracer experiments using velocity fields at 1000 m depth from B-SOSE (see “Offline tracer experiments” in “Methods”). We use B-SOSE output for these experiments because Argo observations are too sparse to accurately reproduce tracer advection pathways. Although B-SOSE underestimates deep eddy temperature anomalies, its velocity field is suitable for these tracer experiments because the simulated EKE at 1000 m is comparable to the Argo-based estimate. By comparing tracer dispersion patterns driven by the full flow (resolving mesoscale eddies) versus mean flow (time-averaged velocity fields), we evaluate the role of eddies in modulating tracer transport in the deep Southern Ocean.

**Fig. 4:**
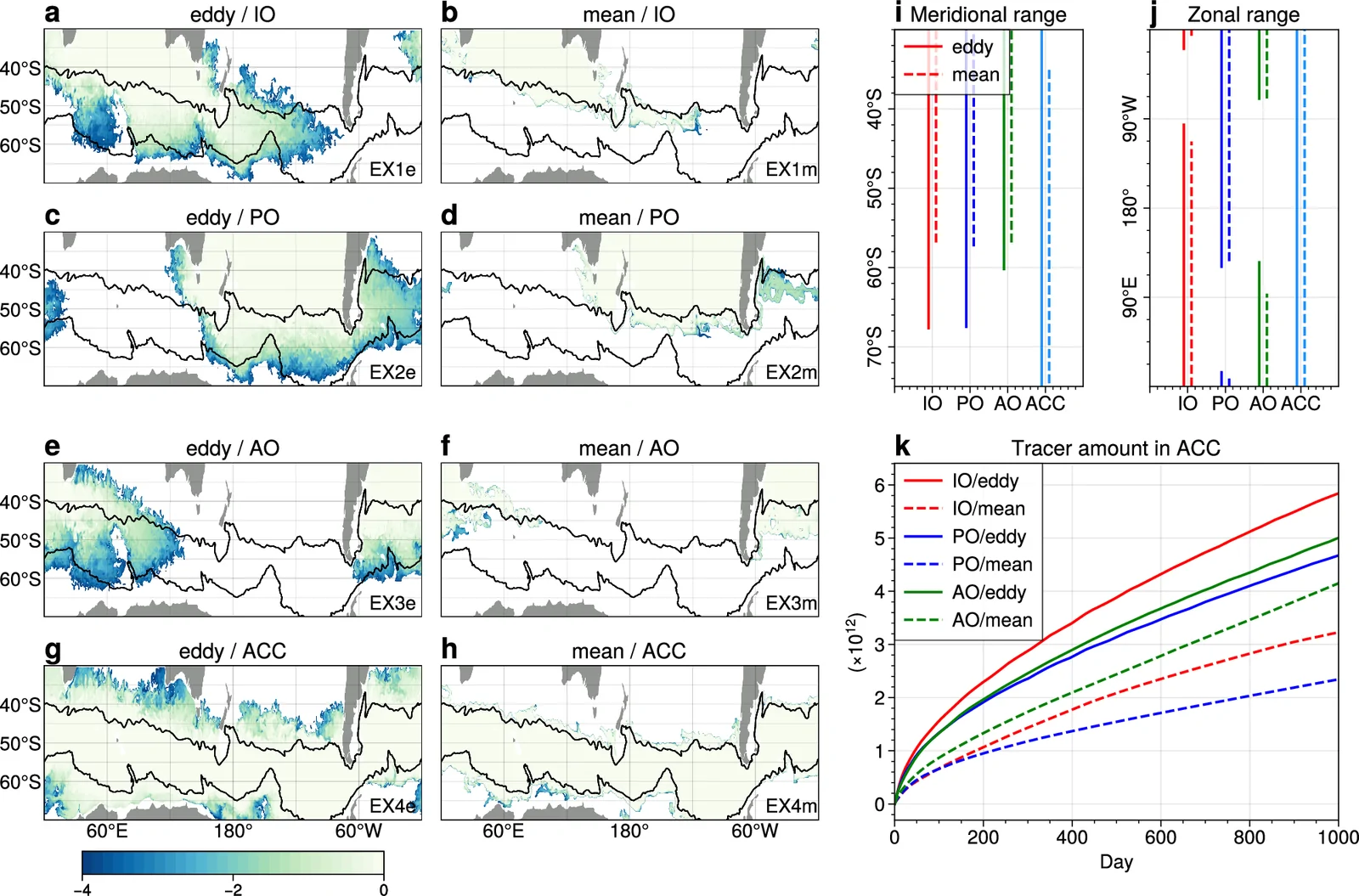
Offline tracer experiments in the deep Southern Ocean. Tracer concentration at 1000 m depth released in four regions and advected by: full flow (eddy-resolving) in the Indian Ocean (**a**), Pacific Ocean (**c**), Atlantic Ocean (**e**), and Antarctic Circumpolar Current (ACC) region (**g**); and by mean flow alone in corresponding regions (**b**, **d**, **f**, **h**). **i** Meridional range of tracer distribution across different basins. **j** Zonal extent of tracer distribution. **k** Temporal series of cumulative tracer amount entering the ACC from the three ocean sectors (Indian, Pacific, Atlantic), with solid and dashed lines representing full flow and mean flow experiments, respectively. Black contours mark the ACC boundaries. Color shading indicates log10 tracer concentration. Note that the initial tracer concentration in these four experiments is 1, which is used to mark the water in different basins.

Notably, the tracer dispersal patterns in eddying simulations closely align with observed Argo trajectories, demonstrating that B-SOSE-based offline experiments successfully replicate tracer transport in the real ocean. In mean-flow experiments, tracers remain predominantly confined along the periphery of the ACC, exhibiting minimal meridional dispersion (Fig. 4b, d, f, h). In contrast, eddy-resolving simulations reveal concurrent zonal and meridional transport, with tracers originating from the Indian, Pacific, and Atlantic basins reaching the southern boundary of the ACC after about 3 years (Fig. 4a, c, e, g). Mesoscale eddies amplify meridional transport of subtropical warm waters by over ten degrees of latitude in both Indian and Pacific sectors, doubling the cross-ACC tracer volume compared to mean-flow simulations (Fig. 4i–k). Transport efficiency under eddying conditions exceeds that of mean-flow scenarios by a factor of two—tracer quantities requiring 1000 days to accumulate in mean-flow simulations are achieved in under 500 days when eddies are present (Fig. 4k). These results confirm that mesoscale eddies facilitate deep subtropical-origin water intrusion toward polar regions and quantitatively show that they enhance both transport magnitude and efficiency by about a factor of two relative to the mean flow.

**Fig. 5:**
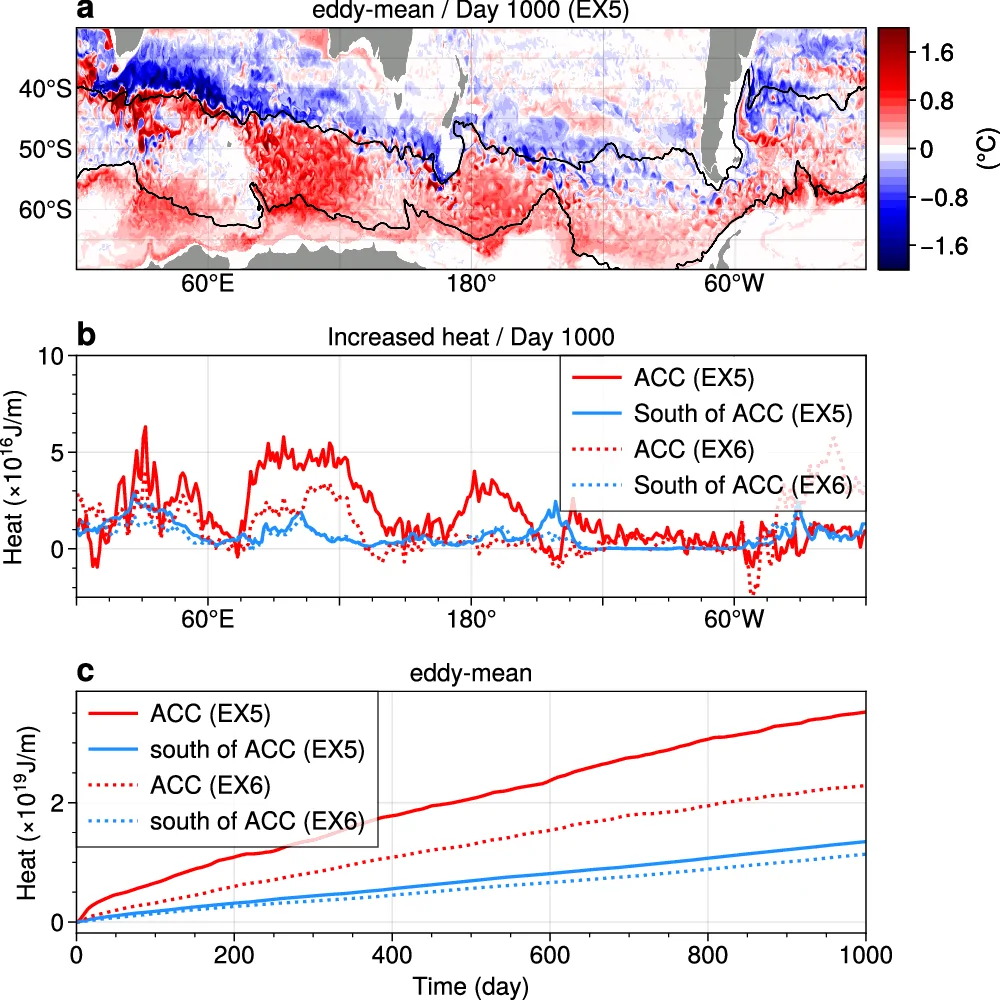
Heat transport diagnosed from tracer experiments. **a** Spatial distribution of temperature difference (eddy-resolving simulation minus mean-flow simulation) at day 1000. Black contours mark boundaries of the Antarctic Circumpolar Current (ACC). **b** Longitudinal distribution of increased heat transport at day 1000 within the ACC (red) and south of the ACC (blue). **c** Temporal evolution of cumulative heat transport difference between eddy-resolving and mean-flow simulations for the ACC region (red) and south of the ACC (blue). In (**b**) and **c**, solid and dashed lines denote EX5 and EX6, respectively. EX5 uses the observed climatological temperature field as the initial tracer, whereas EX6 uses an idealized north-to-south linearly decreasing temperature tracer.

It is necessary to note that a uniformly initialized tracer reflects only the spatial extent of water mass dispersion and cannot diagnose heat transport, which depends on tracer gradients. To address this limitation, we initialize tracer concentration fields using climatological temperature distributions (EX5), enabling direct assessment of temperature evolution over time (Supplementary Fig. 8). The difference between eddy-resolving and mean-flow simulations then reveals eddy-induced temperature anomalies in the deep Southern Ocean (Fig. 5a). As temperature tracers evolve, relatively warm tracer north of the ACC is advected southward into the ACC region. Because the tracer field is not restored or externally replenished in a 2D offline experiment, negative anomalies emerge north of the ACC. Meanwhile, the ACC region exhibits significant warming, particularly in the EKE hotspots where positive temperature anomalies exceed 1.5 °C. Building on previous studies^11,19,36^, the Argo float distributions shown in Fig. 3 and Supplementary Fig. 7 indicate that these deep warm intrusions originate primarily from the Indian Ocean sector.

Integrating the heat content within the northern and southern boundaries of the ACC (Fig. 5b) reveals that EMHT is strongest in the Indian Ocean sector (25°E–150°E), accounting for 63% of the total, followed by the Pacific (150°E–70°W, 27%) and Atlantic (70°W–25°E, 10%) Ocean sectors. In addition, waters originating from the ACC contribute substantially to poleward heat transport by eddies. To test the role of the background temperature field, we conduct an additional sensitivity experiment (EX6) initialized with an idealized north-to-south linearly decreasing temperature tracer (Fig. 5b, c), which retains a simple meridional gradient but removes the basin-scale heterogeneity present in the observed climatology used in EX5. The cumulative heat entering the ACC in EX6 reaches only about 60% of that in EX5, and the relative contributions of the Indian, Pacific, and Atlantic sectors shift to 46%, 8%, and 46%, respectively. This sensitivity experiment confirms that differences in EMHT among the ocean basins are strongly influenced by the magnitude and spatial structure of the background temperature gradients. These results demonstrate the critical role of mesoscale eddies in facilitating cross-frontal heat exchange between subtropical and polar waters at climatological timescales, and highlight a pronounced zonal asymmetry in deep Southern Ocean heat transport, with the Indian Ocean sector serving as the dominant pathway.

## Discussion

The Southern Ocean limits the direct poleward penetration of warm subtropical water because meridional mean flows are weak. Mesoscale eddies, therefore, provide a major pathway for poleward heat transport across the ACC. Yet direct observations of EMHT remain largely limited to the surface where eddy activity is strongest, leaving its deep structure poorly understood. In this study, we use the two-decade accumulation of discrete Argo data to provide the first observation-based estimate of deep EKE and EMHT at climatological scales in the Southern Ocean. We find that climatological EMHT in the deep Southern Ocean reaches at least the same order of magnitude as the surface transport, with its zonally averaged value at 1000 m reaching about half of the surface value, even though deep EKE is only one-fifth of surface levels. Deep EMHT is not visible from satellite observations and thus has been hidden from standard calculations. This substantial eddy heat flux shapes the Southern Ocean’s heat budget and is critical for understanding the future of both the remote Southern Ocean and Antarctic coastline. We also find that, relative to mean flow, mesoscale eddies drive the poleward extension of subtropical water by more than ten degrees of latitude in the deep Southern Ocean, while doubling both warm water volume and transport efficiency. Notably, about 60% of deep EMHT originates from the Indian Ocean sector. These results quantitatively demonstrate the critical role of eddies in facilitating poleward heat transport in the deep Southern Ocean and highlight the importance of warm waters from the Indian Ocean.

It is necessary to note that data scarcity restricts the direct quantification of EMHT to the standard Argo parking depth (1000 m), whereas heat redistribution in the Southern Ocean is inherently a three-dimensional process (Supplementary Fig. 9). Once subtropical warm water is advected across the ACC in the deep Southern Ocean, it is upwelled toward the upper ocean through two mechanisms: isopycnal transport and mixing along tilted density surfaces^25,39,40^ and diapycnal mixing across weakly stratified layers in the region south of the ACC^41^. These processes could collectively enhance basal melt of ice shelves and modulate sea ice distribution^15,42^. In addition, recent studies have revealed a remarkable warming trend in the Indian Ocean sector of the Southern Ocean^43^, which coincides with our findings that this sector dominates EMHT in the deep Southern Ocean. This highlights the critical role of eddies in the Indian Ocean in modulating high-latitude heat balance under global warming that warrants particular attention in future research. Another important point is that the frequent occurrence of subsurface eddies highlights their potentially important role in redistributing heat^44^, which also deserves greater attention. Moreover, mesoscale eddies transport not only heat but also other tracers such as nutrients and dissolved carbon^45,46^. Our findings have important implications for understanding how eddy activity in the deep Southern Ocean influences marine ecosystems and biogeochemical cycles in high-latitude regions.

This study also demonstrates that accurately resolving deep eddy activity and temperature fields is critical for reliable simulations of high-latitude ocean heat balance and Antarctic sea ice variability. Current climate models exhibit persistent warm biases throughout the entire Southern Ocean water column and fail to adequately resolve mesoscale eddies^47,48^, likely contributing to significant discrepancies between simulated and observed sea ice extent^49^. Improving eddy parameterizations to incorporate depth-dependent transport efficiencies, particularly in the Indian Ocean sector, which dominates deep EMHT, may help mitigate these model biases. On the observational front, Argo floats hold promise for unraveling mesoscale variability in the data-scarce deep ocean. Nevertheless, Argo-derived velocities are estimated from float displacements over ~10-day cycles and may thus underestimate variability on timescales shorter than 10 days. Our estimates of deep eddy activity and associated heat transport should therefore be regarded as conservative. While this study mainly focuses on the Southern Ocean, its analytical framework is scalable to the global ocean to characterize deep ocean mesoscale eddy features. Moreover, as Argo datasets continue to expand, future research may leverage enhanced observations to quantify long-term trends in deep eddy activity and their role in climate feedback mechanisms.

## Methods

### Satellite altimetry

The SSALTO/DUACS altimeter products (distributed by AVISO+) provide daily sea surface height, geostrophic currents, and geostrophic velocity anomalies at 1/4° resolution. Here, we use altimetry data spanning 2001 to 2020 to diagnose surface MKE and EKE.

### Argo data

Argo data are collected through an array of autonomous profiling floats distributed throughout the global oceans, providing observations of temperature, salinity, and pressure. These floats operate on a 10-day cycle, spending most of their time (~9 days) drifting passively at a parking depth, typically around 1000 m, to follow ocean currents. At the end of the drift phase, the float quickly descends to a deeper depth, often reaching 2000 m, before ascending to the surface^50^. During this ascent, the float’s sensors capture measurements of temperature, salinity, and pressure at various depths, creating a detailed vertical profile of the water column. Once at the surface, the float transmits the collected data via satellite before descending again to repeat the cycle. Traditional Argos floats stay on the sea surface for 6–18 h for data transmission, while modern Argo floats equipped with Iridium communication require less than 20 min^51^.

Argo floats spend the majority of their cycle time drifting at their parking depth, carried by deep ocean currents. This characteristic allows for the estimation of absolute velocities by calculating the drift distance and duration at the parking depth. Utilizing this methodology, the Scripps Argo Trajectory-based Velocity Product has been developed^52^. This dataset provides absolute velocity estimates derived from quality-controlled Argo trajectory files. Here, a total of 528,315 transmitted velocities at depths of 800–1200 m from 2001 to 2020 are used to calculate deep ocean currents and diagnose eddy intensity in the Southern Ocean. A key advantage of this dataset is that velocity is estimated from the distance and time interval between the extrapolated surface descent position of one cycle and the extrapolated surface arrival position of the next cycle^52^. This extrapolation technique is exclusively applied to traditional Argos floats with more than six valid surface positioning fixes, which reduces contamination from surface drift and provides a more representative estimate of velocity at parking depth. Corrected traditional Argos records, and all Iridium Argos observations together account for 97.8% of the samples used in this study.

### Drifter data

We utilize the Global Drifter Program hourly dataset^53^, which provides trajectories of surface drifters with hourly estimates of position, sea surface temperature, and velocity at a nominal depth of 15 m. This dataset spans from 1987 to 2020, incorporating quality-controlled measurements from over 17,000 drifters globally. In this study, we focus on drifter trajectories south of 30°S, which constitute about 30% of the global dataset. It should be noted that we do not exclude drifters after drogue loss in order to retain sufficient spatial and temporal coverage. We acknowledge that undrogued drifters may be affected by wind, which could introduce additional uncertainty in the drifter trajectories. However, the validation analysis based on the B-SOSE surface particle experiments shows pathways similar to the drifter observations (Fig. 3), supporting the robustness of our interpretation.

### WOA23

The World Ocean Atlas 2023 (WOA23) dataset is used to provide the climatological temperature and density distribution of the Southern Ocean.

### ACC boundary

The northern and southern boundaries of the ACC are defined based on the mean dynamic topography, with the northern boundary at 0.30 m and the southern boundary at −1.11 m^54^. To distinguish the characteristics of eddy activity in different regions, the Southern Ocean between 30°S and the northern boundary of the ACC is divided into three sectors: the Indian (25°–150°E), the Pacific (150°E–70°W), and the Atlantic (70°W–25°E) Ocean sectors.

### B-SOSE

The Biogeochemical Southern Ocean State Estimate^55^ is a data assimilation product designed to provide a dynamically consistent estimate of the Southern Ocean’s physical, biogeochemical, and sea ice properties. B-SOSE integrates observational data (e.g., Argo) into the MITgcm model using an adjoint method and has been validated against satellite and in situ observations to ensure its reliability. The dataset spans the region from 30°S to 78°S with an eddy-permitting horizontal resolution of 1/6° and 52 vertical layers of varying thickness. B-SOSE iteration 122 provides daily-averaged physical and biogeochemical variables, including temperature, salinity, velocity fields, and nutrient concentrations from 2013–2017. B-SOSE has been widely applied in studies of mesoscale eddy activity in the Southern Ocean^56,57^. In this study, we mainly use daily-averaged velocity fields to conduct offline particle and tracer experiments.

### Composite vertical structure of mesoscale eddies

The vertical structure of eddy-associated temperature anomalies is used here as a useful thermodynamic proxy for the strength and vertical extent of mesoscale eddies. Following the widely used Argo–eddy compositing approach^9^, temperature anomalies from Argo profiles around mesoscale eddies are projected into a normalized eddy-centered coordinate system. We use 618,110 Argo temperature profiles collected during 2001–2020, including only profiles with quality flag “A”. Temperature anomalies are defined relative to the monthly climatological mean field, and composite sections are constructed over a cross-eddy distance from −2 to 2 eddy radii. The eddy center and radius are taken from the Mesoscale Eddy Trajectory Atlas (META3.1exp), and only eddies with lifetimes longer than 30 days between 30°S and 65°S are retained. The same compositing procedure is also applied to B-SOSE output, with mesoscale eddies identified following the same detection criteria as in META3.1exp.

### MKE and EKE

The MKE is defined as:

1 $${MKE}=\frac{1}{2}({\overline{u}}^{2}+{\overline{v}}^{2}),$$

where (u,v) is the 2D horizontal velocity vector and the bar indicates the time average. The EKE is defined as:

2 $${EKE}=\frac{1}{2}(\overline{{{u}^{{\prime} }}^{2}+{{v}^{{\prime} }}^{2}}),$$

where the prime indicates the velocity perturbation from the mean flow. The surface MKE and EKE can be directly calculated using the surface geostrophic currents and their anomalies from satellite altimetry.

While satellite-derived products offer high spatiotemporal-resolution observations of EKE at the surface, no comparable data exist for the deep Southern Ocean. To fill this observational gap, we seek to diagnose deep MKE and EKE using the Argo velocity dataset. This product estimates deep (~1000 m) currents by averaging displacements between consecutive surfacing positions over approximately 10-day intervals, capturing low-frequency motions (periods >10 days) but being unable to resolve high-frequency processes (<10 days).

To assess the contribution of high-frequency motions, we use B-SOSE velocity fields to calculate the temporal power spectrum of kinetic energy at the surface and 1000 m over 40–60°S. High-frequency variability (<10 days) accounts for about 20% of the total kinetic energy at the surface, but only 1.2% at 1000 m. We further examine four publicly available deep mooring records in the Southern Ocean (Supplementary Table 1; WHOI 835: 40.13°S, 16.57°E, 958 m, Feb 1985–Feb 1987; SAZ47-2010: 46.83°S, 141.65°E, 1100 m, Sep 2010–Aug 2011; SAZ47-2015: 46.83°S, 141.65°E, 1100 m, Mar 2015–Mar 2016; Drake M7: 58.96°S, 58.10°W, 980 m, Jan 2006–Dec 2007). The mooring spectra show that high-frequency motions account for less than 8% of the total kinetic energy at these depths (Supplementary Fig. 2). These results indicate that the deep flow is dominated by low-frequency (>10 days) motions, supporting the use of Argo-derived velocities to represent mesoscale eddy variability in the deep Southern Ocean.

Since high-frequency motions are negligible at 1000 m, the observed Argo velocity can be treated as the instantaneous velocity in our analysis. The velocity anomaly is defined relative to the local ensemble mean as ${u}^{{\prime} }=u-\bar{u}$, where $\bar{u}$ is the mean velocity calculated from all Argo samples within each 2° × 1° grid cell. The same procedure is applied to the meridional velocity v to obtain $v{\prime}$. This approach enables the diagnosis of deep MKE and EKE using discrete Argo data.

### EMHT

The EMHT is defined as:

3 $$Q=\rho {C}_{P}\left\langle v^{\prime} T^{\prime} \right\rangle,$$

where ρ is the density, *C*_*P*_ = 4000 J kg^−1^ °C^−1^ is the specific heat of sea water, $v^{\prime}$ is the meridional velocity perturbation, and ${T}^{{\prime} }=T-\bar{T}$ is the temperature perturbation relative to the monthly climatological temperature field from gridded Argo data. Here, T is the drift temperature from the Argo trajectory-based velocity product, and both $v{\prime}$ and ${T}^{{\prime} }$ are assigned to the midpoint of the corresponding drift cycle. The bracket denotes the ensemble average within 2° × 1° grid cells. While similar approaches have been applied to earlier sparse float observations^19,58^, we extend this framework using two decades of Argo-derived velocity and temperature data to quantify deep EMHT over the entire Southern Ocean. Chinn and Gille (2007) suggested that robust eddy heat flux estimates require about 100 samples in a 2° × 2° grid cell^58^. Our Argo-based estimates satisfy this criterion over most of the Southern Ocean, except poleward of 60°S.

To compare with deep EMHT, we estimate surface EMHT using satellite data. The resulting spatial distribution and magnitudes are in good agreement with recent estimates^59,60^. Sea surface temperature data, used for calculating surface temperature perturbations, are derived from the Optimum Interpolation Sea Surface Temperature (OISST) dataset.

### Trajectory analysis

To examine differences in eddy-driven transport between the surface and deep ocean, we compare the trajectories of surface drifters and Argo floats. All samples are divided into four regions: the Indian, Pacific, Atlantic Ocean sectors, and the ACC region. For each float, we track its trajectory for 1000 days after first entering a given region, with each trajectory recorded as an individual sample. To minimize potential biases arising from different initial sampling locations, the initial positions in the Indian, Pacific, and Atlantic sectors are further restricted to a narrow band within 3° north of the ACC. The number of drifter samples in the Indian, Pacific, and Atlantic Ocean sectors and the ACC region are 1062, 594, 749, and 2427, respectively. The corresponding numbers of Argo float samples are 737, 610, 484, and 1973, respectively. Spatial probability density is then calculated for each set of trajectories and normalized such that the total probability sums to one.

A caveat is that Argo floats are profiling instruments rather than ideal Lagrangian particles. Therefore, 1000-day Argo float trajectories should not be interpreted as exact trajectories of water parcels at 1000 m. This limitation is particularly relevant for pre-Iridium Argo floats, for which surface drift during data transmission may introduce additional uncertainty into the total displacement. We therefore interpret the Argo-based distributions as broad float displacement patterns rather than precise Lagrangian pathways of individual deep water parcels.

### Validation of Argo-based estimates

To evaluate the robustness of the Argo-based diagnostics, we conduct an independent validation using daily surface and 1000 m velocity fields from B-SOSE to advect Lagrangian particles. Particles are initialized on a 0.4° × 0.2° grid, yielding more than 160,000 particles, and are integrated continuously from 1 January 2013 to 31 December 2017. Zonal and meridional velocities are then reconstructed from particle displacements at 10-day intervals, producing approximately 29 million velocity samples, far exceeding the ~530,000 samples contained in the Argo velocity product. To mimic the sparse sampling of the Argo observations, we randomly subsample the B-SOSE-derived velocities to match the exact number of Argo samples. These subsampled datasets are then used to estimate MKE, EKE, and EMHT at both the surface and 1000 m, and the resulting particle-based estimates are compared with the corresponding gridded B-SOSE estimates (Supplementary Figs. 3 and 5). The random subsampling is repeated 50 times to ensure statistical robustness. For the observational Argo-based estimates, the available samples are fixed and too sparse for an equivalent random subsampling experiment. We therefore use 1000 bootstrap resamplings to quantify the uncertainty of the observed MKE, EKE, and EMHT estimates.

### Offline tracer experiments

We conduct a series of two-dimensional offline tracer simulations using the MITgcm (Table 1). The computational domain and horizontal resolution of the tracer model are consistent with those of B-SOSE. The evolution of the passive tracer is completely determined by the horizontal velocity fields (u,v) at 1000 m depth from B-SOSE.

**Table 1:** Configuration of tracer experiments

Experiment no.	Initial tracer setup	Velocity field
EX1e & EX1m	Initialize and relax the tracer concentration to 1 in the Indian Ocean. All other regions are set to 0 without relaxation.	Daily (e) and climatological mean (m)
EX2e & EX2m	Similar to EX1 but applied to the Pacific Ocean	
EX3e & EX3m	Similar to EX1 but applied to the Atlantic Ocean	
EX4e & EX4m	Similar to EX1 but applied to the ACC	
EX5e & EX5m	Initialize the tracer to the climatological temperature field from WOA23	
EX6e & EX6m	Initialize tracer with an idealized north-to-south linearly decreasing temperature field	
EX7	Initialize and relax the tracer concentration to 1 at the surface north of the ACC	Daily three-dimensional
EX8	Initialize and relax the tracer concentration to 1 at 1000 m north of the ACC	

Three types of tracer initialization are implemented (Supplementary Fig. 8). In the first set of experiments (EX1 to EX4), designed to examine water mass dispersion from different basins, tracers are initialized with a concentration of 1 in four regions—the Indian, Pacific, Atlantic Ocean sectors, and ACC region—and are continuously relaxed to this value. The rest of the domain is initialized at 0 with no relaxation. In the second set of experiments (EX5), tracers are initialized using climatological temperature fields from the WOA23, allowing us to quantitatively diagnose heat transport based on the tracer distribution. In the third set of experiments (EX6), tracers are initialized with an idealized north-to-south linearly decreasing temperature field to assess the role of the meridional temperature gradient. To quantify the contributions of mesoscale eddies and mean flow, all experiments (EX1 to EX6) are conducted using two velocity field configurations: daily velocity fields (resolving mesoscale eddies) and climatological mean flow fields. Each experiment is integrated for 1000 days starting from January 1, 2013. In our analysis of EX1–EX4, a minimum tracer concentration threshold of 10^−4^ is used because smaller thresholds have little effect on the integrated tracer amount (not shown). To assess the sensitivity of the EX1–EX4 tracer diagnostics to release timing, we also conduct four additional eddy-resolving experiments initialized 90, 180, 270, and 360 days after January 1, 2013. The resulting ensemble spread is quite small, with spatial-range standard deviations below 0.2°, and the variation range of total tracer entering the ACC generally below 2% after 400 days (Supplementary Fig. 10).

Given that Southern Ocean EMHT is a three-dimensional process, we also conduct an additional set of three-dimensional tracer experiments. The daily three-dimensional velocity fields (u,v,w) and the vertical grid configuration are both taken from B-SOSE. Two tracer initializations are designed. In EX7, the tracer is initialized at the surface north of the ACC with a concentration of 1 and is continuously relaxed toward this value, representing surface heat input from lower latitudes. In EX8, the tracer is initialized at 1000 m north of the ACC in the same way, allowing a direct comparison of the relative impacts of surface and deep EMHT on high-latitude regions.

## Supplementary information

Supplementary Information

Transparent Peer Review file

## Data Availability

All data used in this study are publicly available. The META3.1exp dataset is available at https://www.aviso.altimetry.fr/en/data/products/value-added-products/global-mesoscale-eddy-trajectory-product/meta3−1-exp-dt.html. The surface drifter data are available at https://www.aoml.noaa.gov/phod/gdp/hourly_data.php. The Argo velocity data can be found at https://library.ucsd.edu/dc/collection/bb6630688j. The Argo profile data are available at https://argo.ucsd.edu/data/argo-data-products/. The WOA23 dataset can be accessed at https://www.ncei.noaa.gov/products/world-ocean-atlas. The B-SOSE data can be downloaded from https://sose.ucsd.edu/BSOSE6_iter122_solution.html. The satellite altimetry data can be found at https://www.aviso.altimetry.fr/en/data/products/. The OISST dataset is available at https://www.ncei.noaa.gov/products/climate-data-records/sea-surface-temperature-optimum-interpolation. The mooring records are available from the following repositories: WHOI 835 from https://cmrecords.net/quick/southern/agulret/agulret.html, SAZ47-2010 and SAZ47-2015 from https://thredds.aodn.org.au/thredds/catalog/IMOS/DWM/SOTS/catalog.html, and Drake M7 from https://www.seanoe.org/data/00500/61186/. The processed datasets generated in this study are available from Zenodo at https://doi.org/10.5281/zenodo.21739996.

## Code availability

The MITgcm model is available at https://mitgcm.org/source-code/. The code used in this study is available from https://doi.org/10.5281/zenodo.21739996.
