# Supplementary material for: Observed poleward heat transport by mesoscale eddies in the deep Southern Ocean
Source: Nat Commun. 2026 Aug 12;17:9664. doi: 10.1038/s41467-026-76674-2 (PMC13558553; doi:10.1038/s41467-026-76674-2)
Supplement: Supplementary file 1 [file 41467_2026_76674_MOESM1_ESM.pdf]

Supplementary information for

Observed poleward heat transport by mesoscale eddies in the deep Southern Ocean

Tongya Liu[1]*, Xiaoming Zhai[2], Qingyou He[3], Andrew McC. Hogg[4], and Dake Chen[1,5]

1 State Key Laboratory of Satellite Ocean Environment Dynamics, Second Institute of Oceanography, Ministry of Natural Resources, Hangzhou, China

2 School of Environmental Sciences, University of East Anglia, Norwich, UK

3 State Key Laboratory of Tropical Oceanography, South China Sea Institute of Oceanology, Chinese Academy of Sciences, Guangzhou, China

4 Australia's Climate Simulator (ACCESS-NRI) and Research School of Earth Sciences, Australian National University, Canberra, Australia

5 Southern Marine Science and Engineering Guangdong Laboratory (Zhuhai), Zhuhai, China

Corresponding author: liutongya@sio.org.cn

This file includes:

Supplementary Fig. 1: Observed temperature distributions from World Ocean Atlas 2023.

Supplementary Fig. 2: Temporal spectra of kinetic energy from Biogeochemical Southern Ocean State Estimate and deep mooring observations .

Supplementary Fig. 3: Validation of particle-based mean kinetic energy and eddy kinetic energy using Biogeochemical Southern Ocean State Estimate.

Supplementary Fig. 4: Composite vertical structure of mesoscale eddy temperature anomalies from Argo and Biogeochemical Southern Ocean State Estimate.

Supplementary Fig. 5: Validation of particle-based eddy meridional heat transport estimates using Biogeochemical Southern Ocean State Estimate.

Supplementary Fig. 6: Argo trajectories from the Indian Ocean sector.

Supplementary Fig. 7: Distribution of trajectory durations for surface drifters and Argo floats.

Supplementary Fig. 8: Initial tracer distributions.

Supplementary Fig. 9: Three-dimensional tracer dispersion.

Supplementary Fig. 10: Sensitivity of tracer diagnostics to release-time perturbations.

Supplementary Table 1: Deep mooring records in the Southern Ocean used in this study.

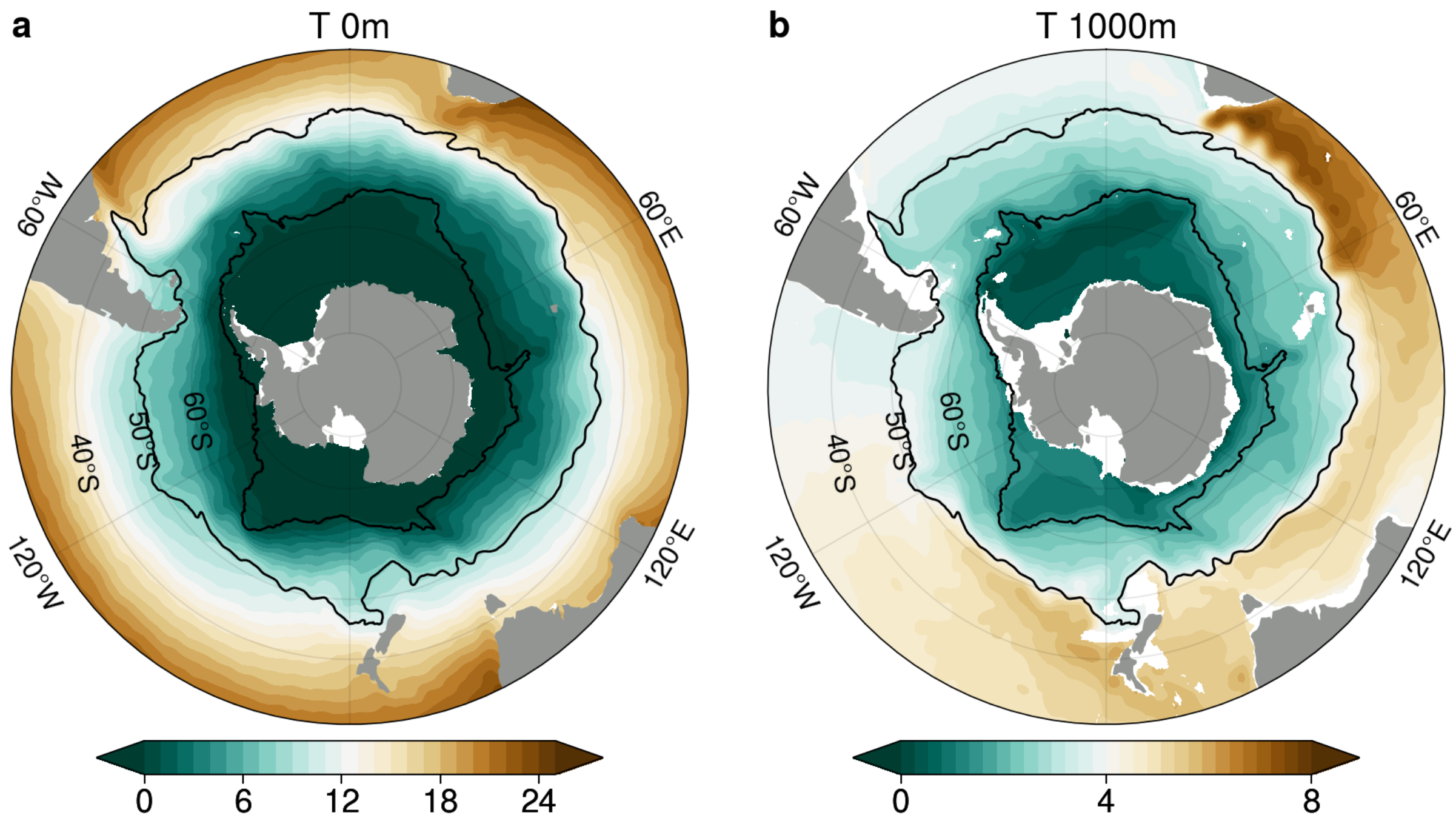

Supplementary Fig. 1 | Observed temperature distributions from World Ocean Atlas 2023. **a**, surface temperature. **b,** temperature at 1000 m depth. Black contours mark the northern and southern boundaries of the Antarctic Circumpolar Current.

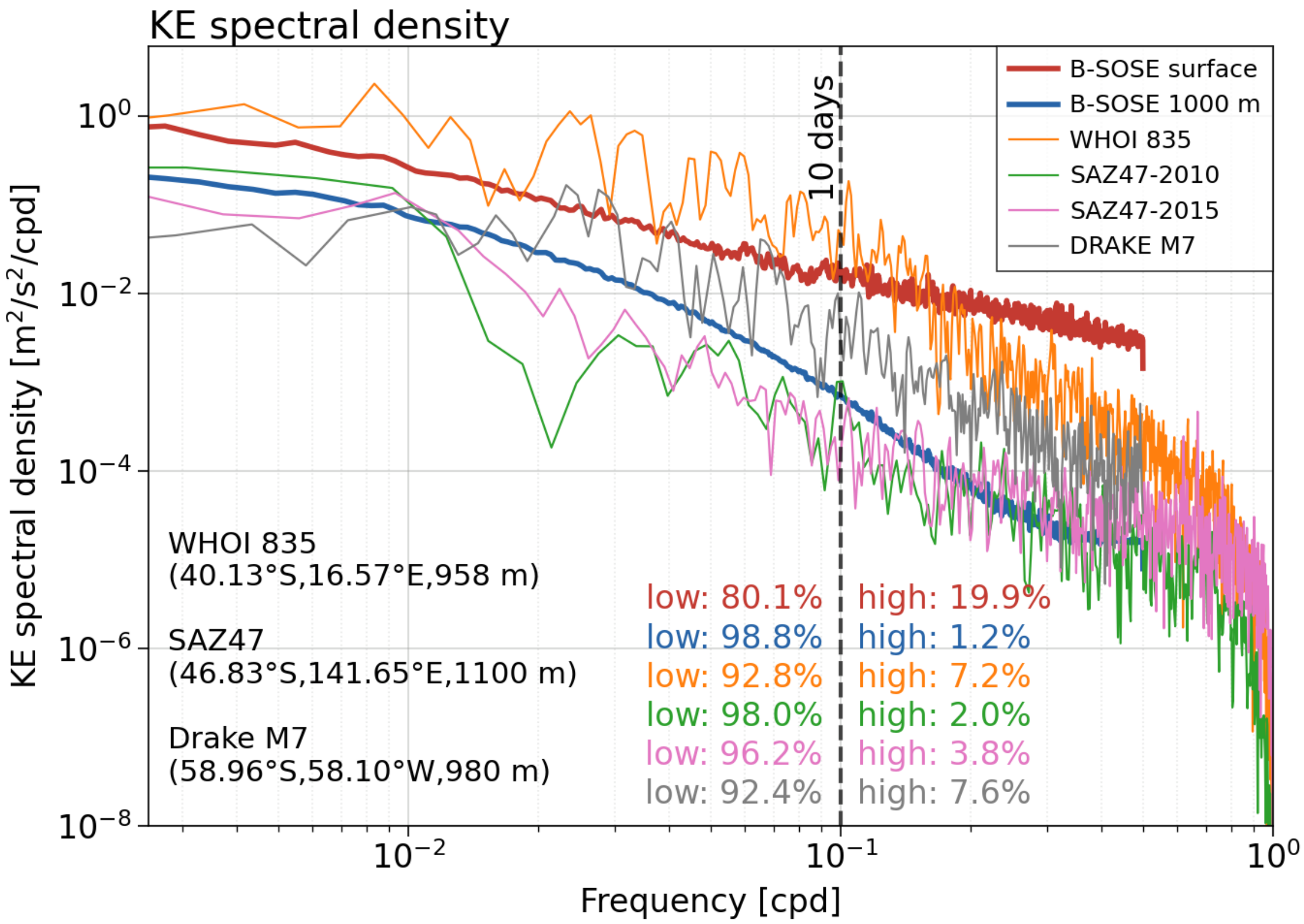

Supplementary Fig. 2 | Temporal spectra of kinetic energy from Biogeochemical Southern Ocean State Estimate (B-SOSE) and deep mooring observations. Temporal power spectra of kinetic energy derived from spatially averaged B-SOSE velocity fields over the Southern Ocean at the surface (red) and 1000 m (blue). The four mooring spectra are from WHOI 835 (40.13°S, 16.57°E, 958 m; orange), SAZ47-2010 (46.83°S, 141.65°E, 1100 m; green), SAZ47-2015 (46.83°S, 141.65°E, 1100 m; pink), and Drake M7 (58.96°S, 58.10°W, 980 m; grey). The WHOI 835, SAZ47-2010 and SAZ47-2015 records have 30 min sampling and are smoothed in this study with a 1-day running mean. The Drake M7 record is distributed as daily output with 2-day smoothing. The dashed vertical line marks a period of 10 days. Percentages indicate the fractions of kinetic energy contained in low-frequency (>10 days) and high-frequency (<10 days) motions.

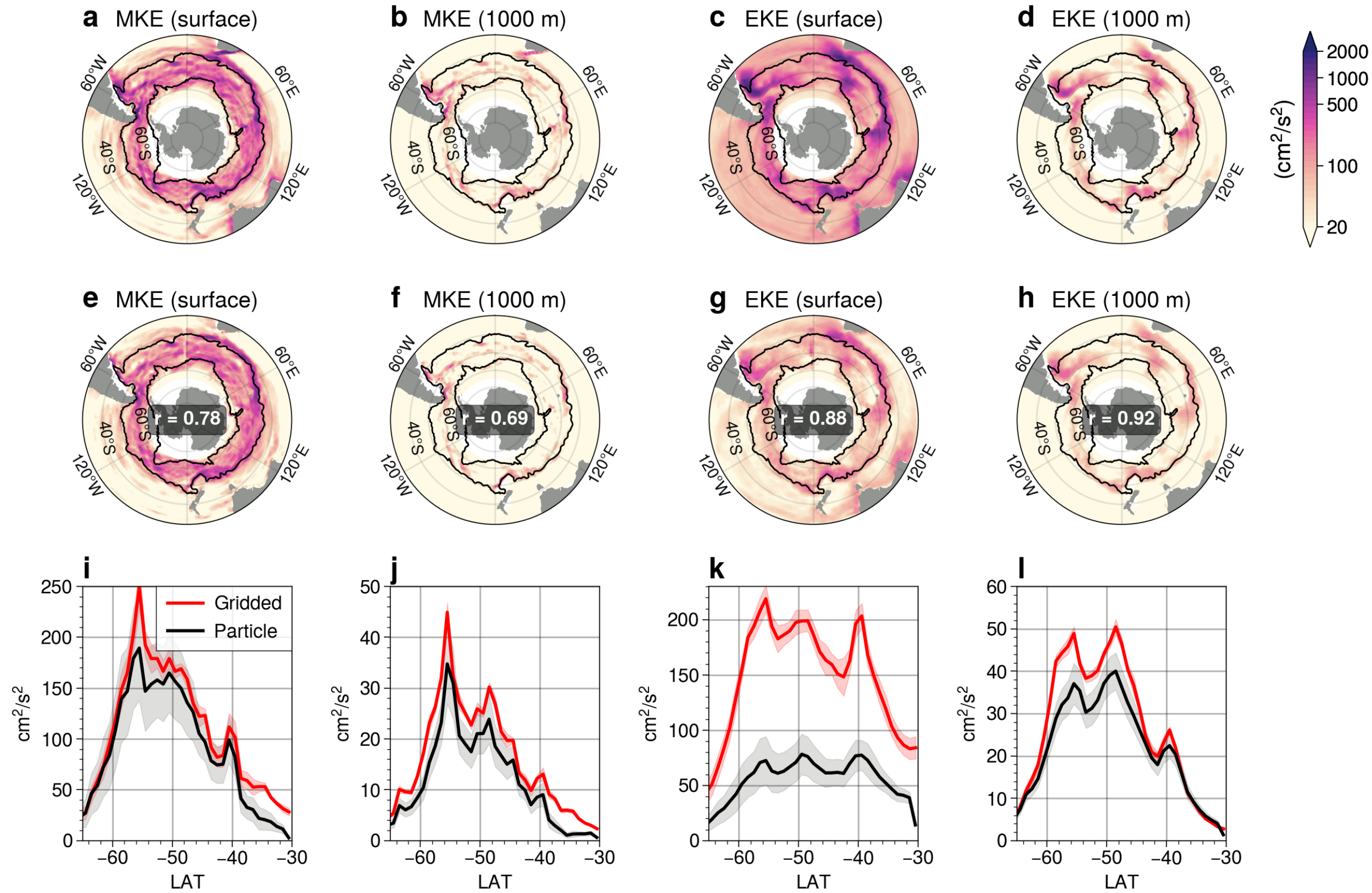

Supplementary Fig. 3 | Validation of particle-based mean kinetic energy (MKE) and eddy kinetic energy (EKE) using Biogeochemical Southern Ocean State Estimate (B-SOSE). a–d, Gridded B-SOSE estimates of surface MKE (**a**), MKE at 1000 m (**b**), surface EKE (**c**), and EKE at 1000 m (**d**). **e–h**, As in **a–d**, but for estimates based on particles advected by B-SOSE velocity fields. Numbers in **e–h** indicate the spatial correlation with the corresponding gridded B-SOSE estimates. **i–l**, Zonal-mean comparisons between the gridded B-SOSE estimates and particle-based estimates for surface MKE (**i**), MKE at 1000 m (**j**), surface EKE (**k**), and EKE at 1000 m (**l**). Red lines denote gridded B-SOSE estimates, with shading showing the standard deviation from monthly climatology. Black lines denote particle-based estimates, with shading indicating the standard deviation from 50 random subsamples. Random subsampling is performed to match the sample size of the Argo velocity product used in the analysis.

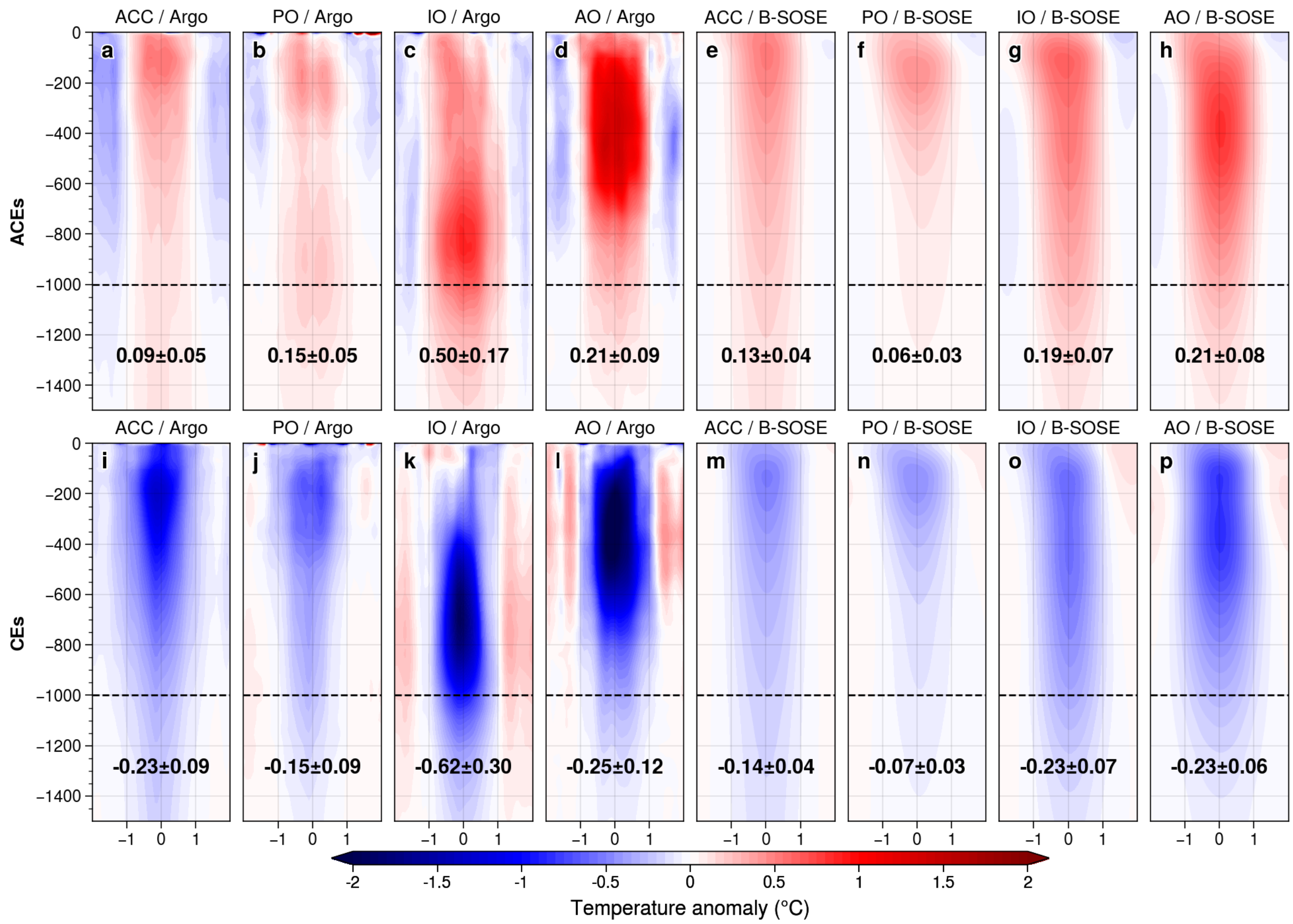

Supplementary Fig. 4 | Composite vertical structure of mesoscale eddy temperature anomalies from Argo and Biogeochemical Southern Ocean State Estimate (B-SOSE). a–d, Composite temperature anomalies for anticyclonic eddies derived from Argo in the Antarctic Circumpolar Current (ACC) (**a**), Pacific (**b**), Indian (**c**), and Atlantic (**d**) sectors. **e–h,** Same as **a–d**, but derived from B-SOSE. **i–l,** Composite temperature anomalies for cyclonic eddies derived from Argo in the ACC (**i**), Pacific (**j**), Indian (**k**), and Atlantic (**l**) sectors. **m–p,** Same as **i–l**, but derived from B-SOSE. Numbers in each panel indicate the mean temperature anomaly ± one standard deviation within one normalized eddy radius of the eddy center. The standard deviation is calculated across all individual temperature-anomaly samples. Temperature anomalies are calculated relative to the monthly climatological mean field.

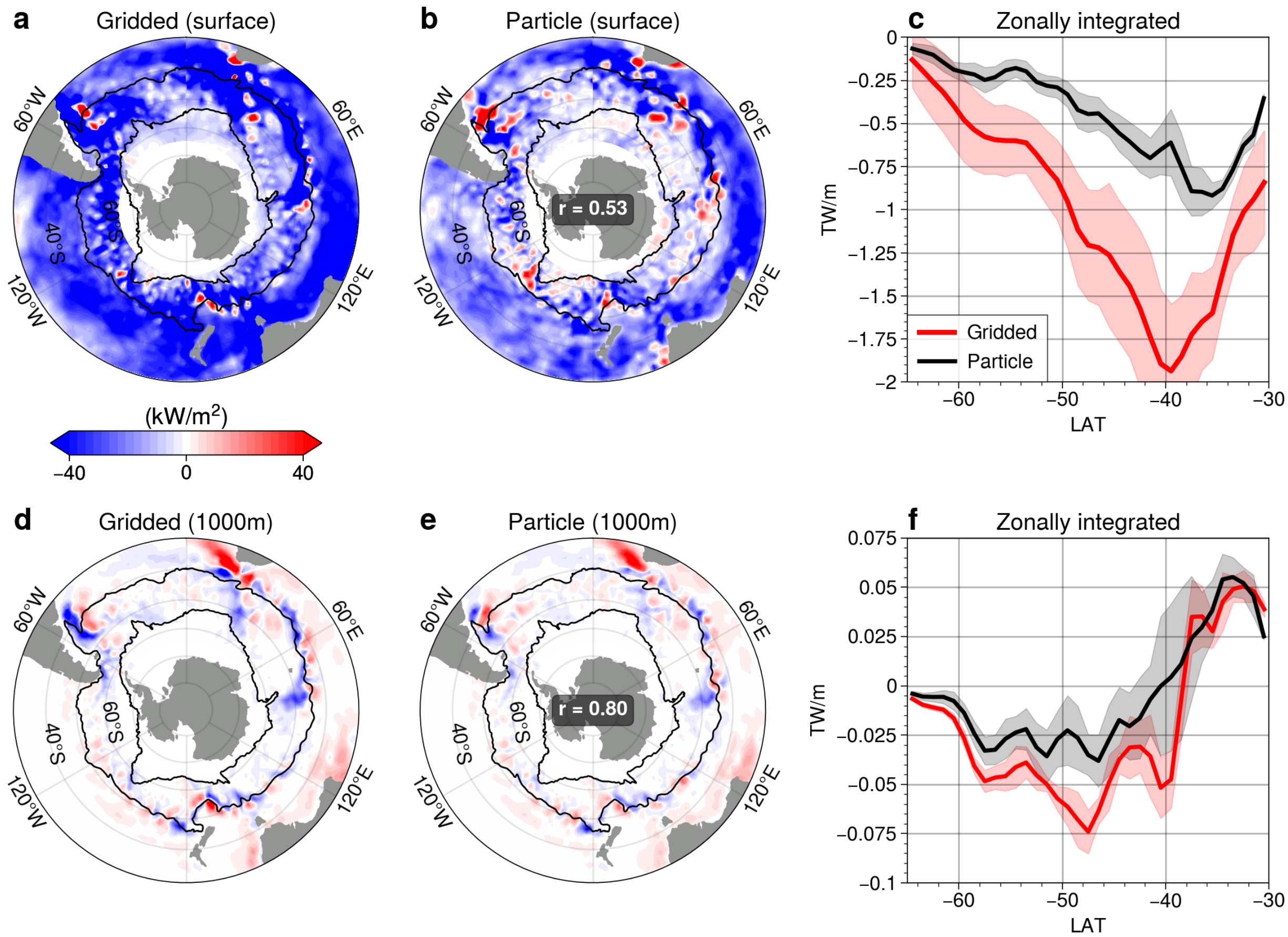

Supplementary Fig. 5 | Validation of particle-based eddy meridional heat transport (EMHT) estimates using Biogeochemical Southern Ocean State Estimate (B-SOSE). **a**,**b**, Surface EMHT estimated from gridded B-SOSE fields (**a**) and from particles advected by B-SOSE fields (**b**). **c**, Zonal-mean comparison of surface EMHT between the gridded B-SOSE and particle-based estimates. **d**,**e**, As in **a**,**b**, but for EMHT at 1000 m. **f**, Zonal-mean comparison of EMHT at 1000 m between the gridded B-SOSE and particle-based estimates. Numbers in **b** and **e** indicate the spatial correlation with the corresponding gridded B-SOSE estimates. In **c** and **f**, red lines denote gridded B-SOSE estimates, with shading showing the standard deviation from monthly climatology, and black lines denote particle-based estimates, with shading indicating the standard deviation from 50 random subsamples. Random subsampling is performed to match the sample size of the Argo velocity product used in the analysis.

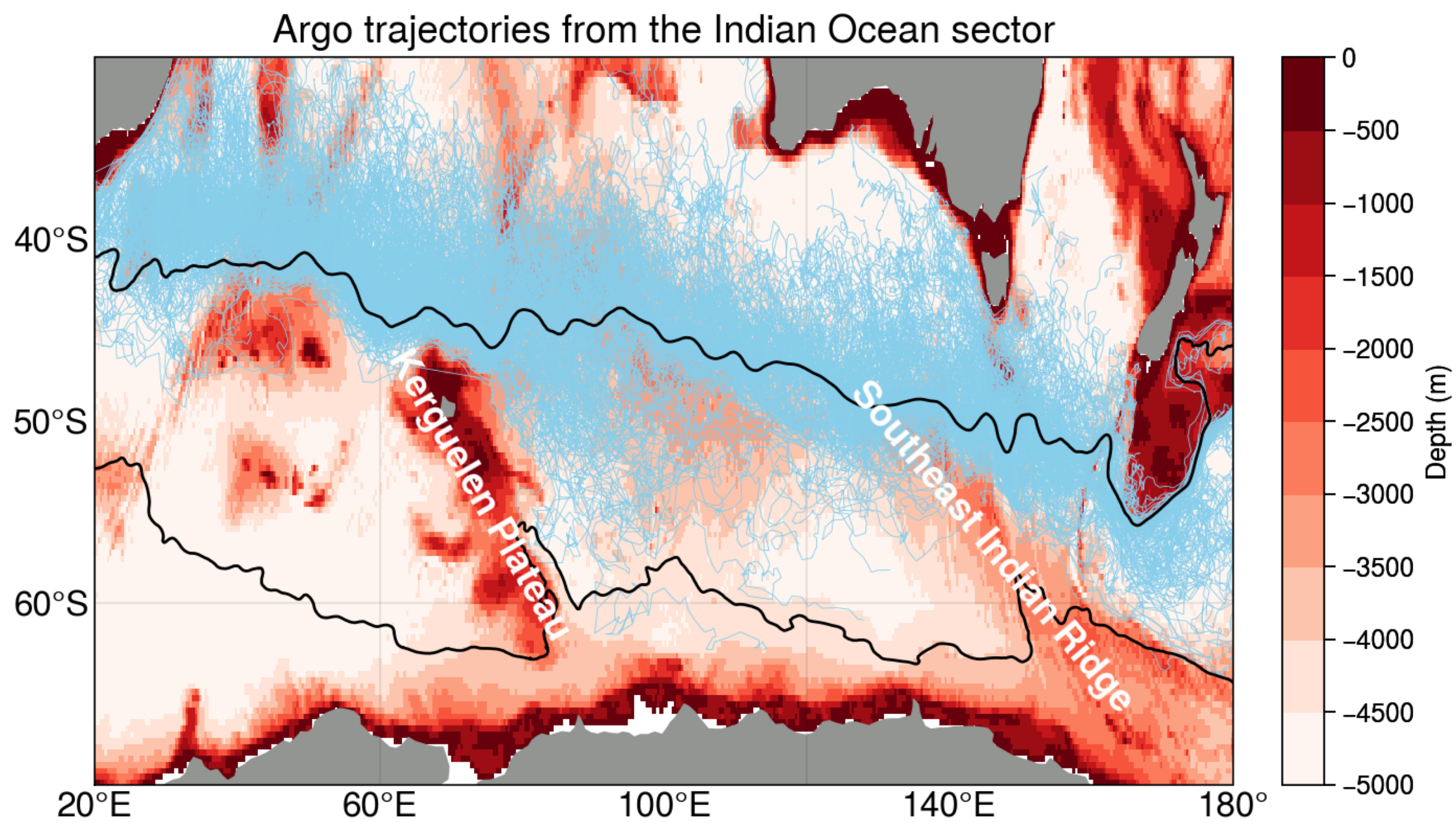

Supplementary Fig. 6 | Argo trajectories from the Indian Ocean sector. Argo trajectories corresponding to Fig. 3b are shown by blue lines. Colour shading indicates bathymetry from ETOPO1. The Kerguelen Plateau and Southeast Indian Ridge are marked. Black lines denote the northern and southern boundaries of the Antarctic Circumpolar Current.

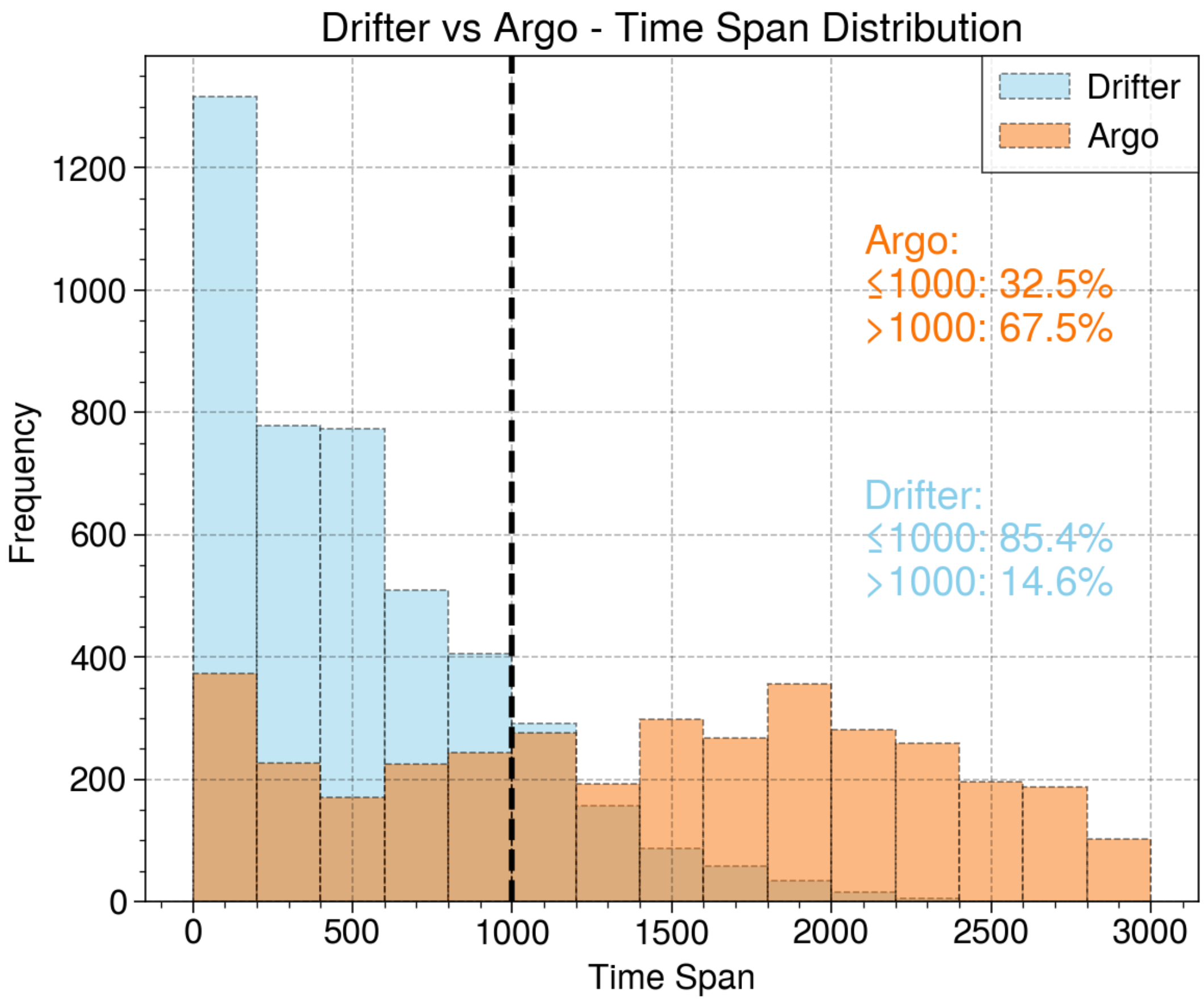

Supplementary Fig. 7 | Distribution of trajectory durations for surface drifters and Argo floats. Histogram of trajectory durations used in the trajectory analysis. Blue bars denote surface drifters, and orange bars denote Argo floats.

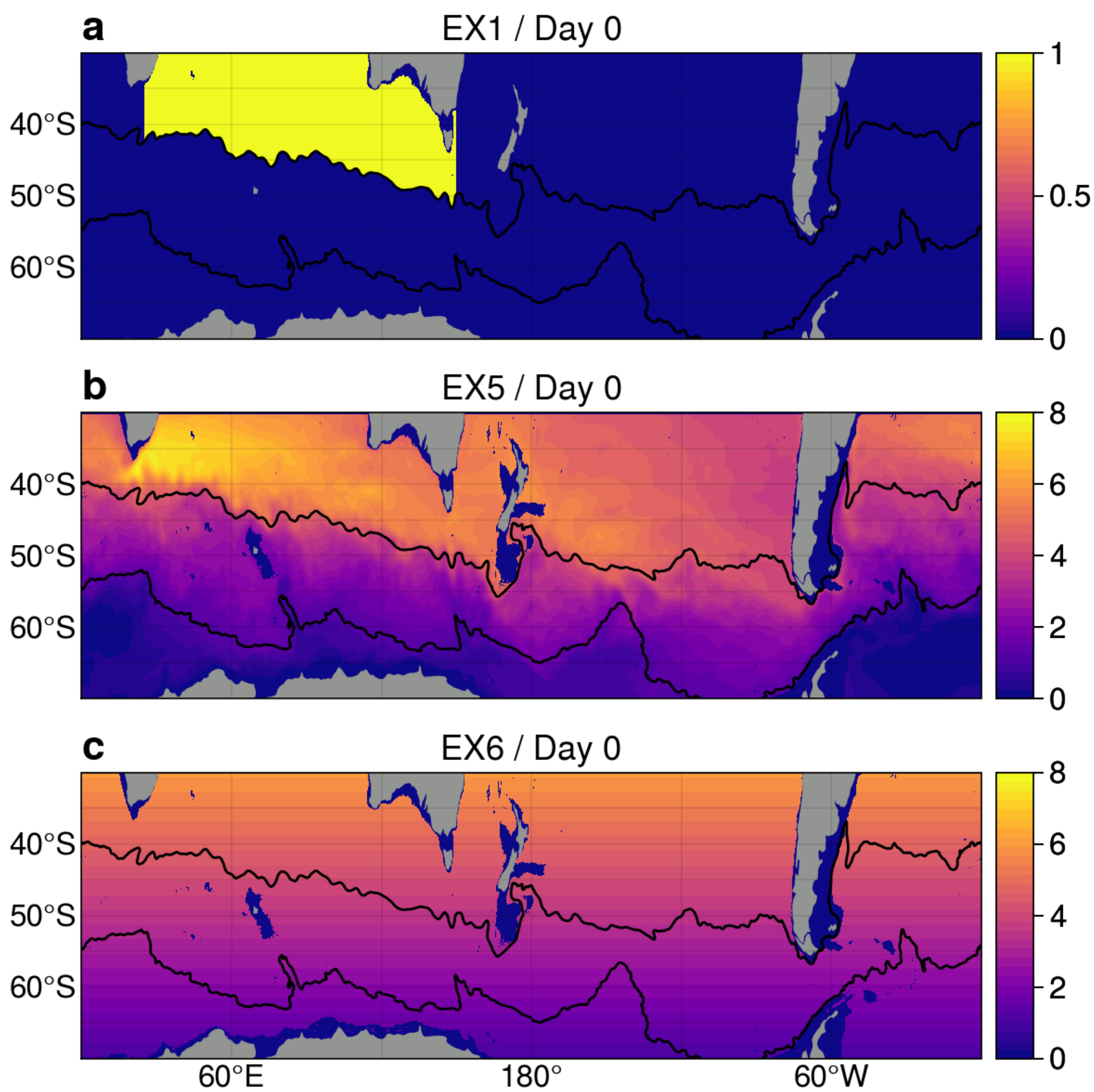

Supplementary Fig. 8 | Initial tracer distributions. Spatial distributions of the initial tracer concentration for **a**, EX1, **b**, EX5 and **c**, EX6.

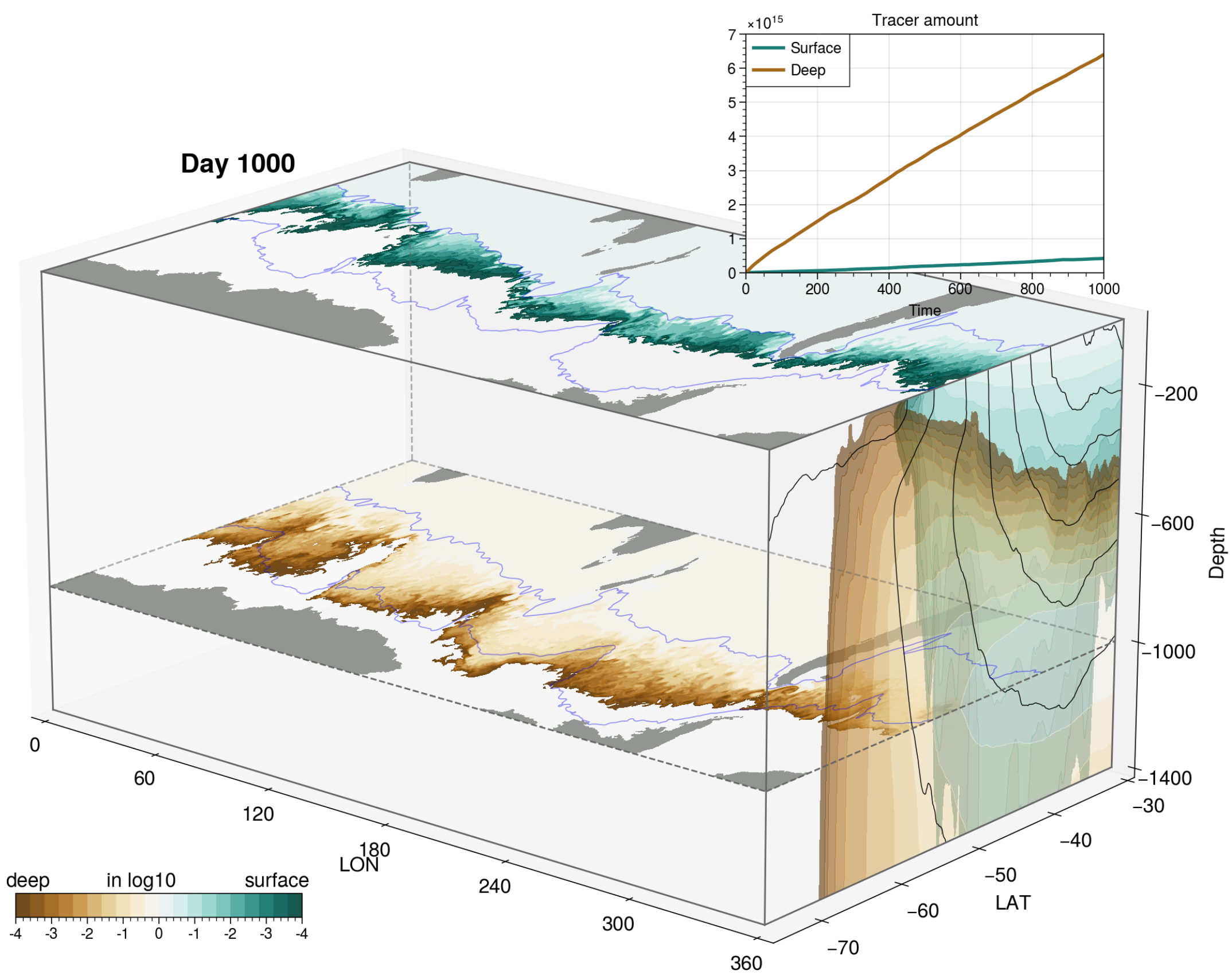

Supplementary Fig. 9 | Three-dimensional tracer dispersion. The two horizontal planes show tracer concentration at the surface and 1000 m at day 1000. The tracer is initialized to 1 north of the Antarctic Circumpolar Current (ACC) and continuously restored to 1 in this source region. The offline experiment is driven by the three-dimensional B-SOSE velocity field. Green and red denote tracers released from the surface and 1000 m, respectively. Blue solid lines mark the northern and southern boundaries of the ACC. The right-hand sections show zonal-mean tracer concentration and temperature profiles at day 1000. The inset time series shows the cumulative tracer amount crossing the ACC, with red and green lines denoting contributions from the 1000 m and surface tracers, respectively.

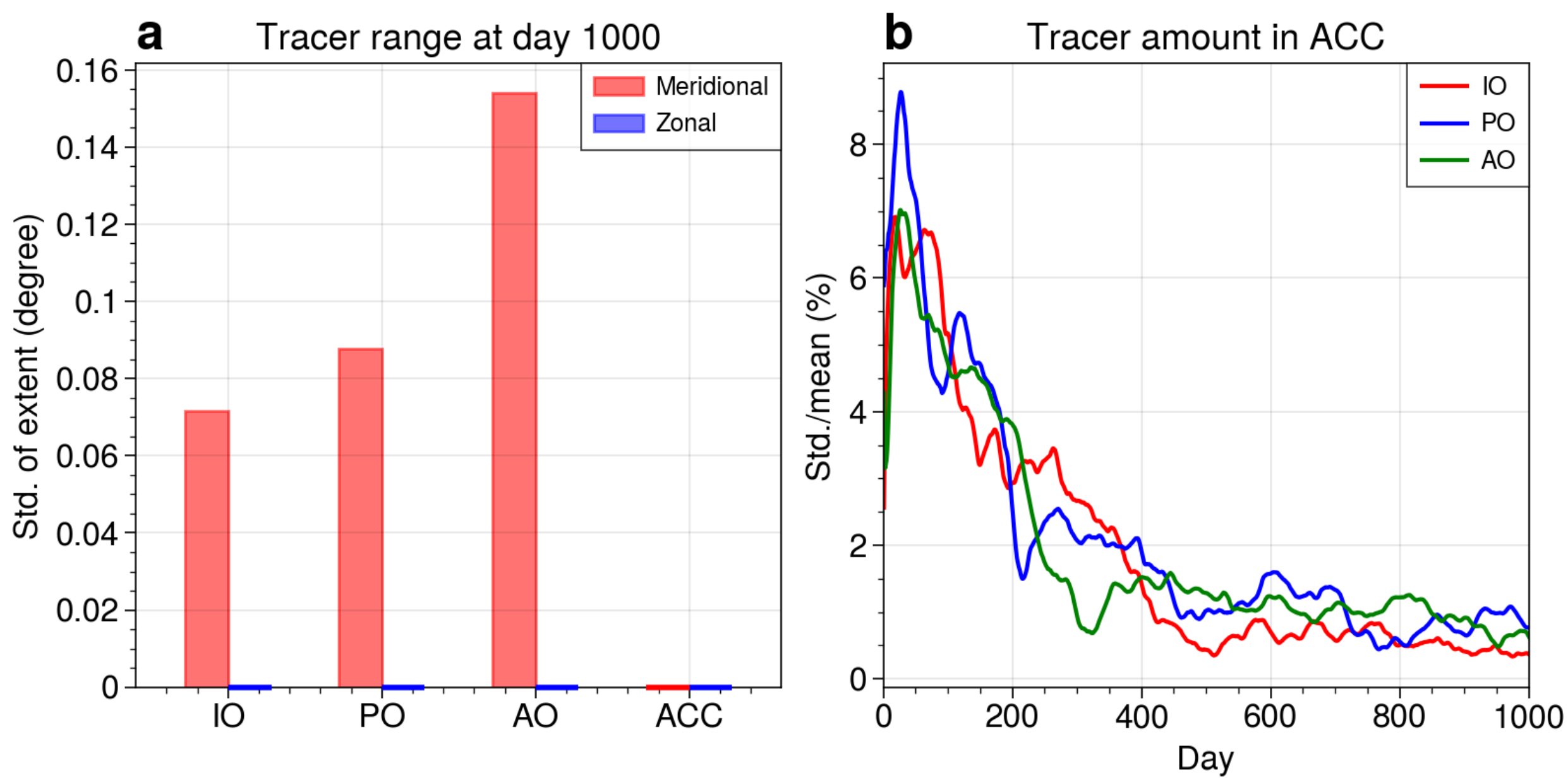

Supplementary Fig. 10 | Sensitivity of tracer diagnostics to release-time perturbations. a, Standard deviation of the meridional and zonal extents of tracer distributions at day 1000 across the five eddy-resolving experiments. **b**, Time-dependent ratio of the standard deviation to the ensemble mean of cumulative tracer amount entering the ACC, calculated across the five experiments. The five experiments use velocity fields initialized on 1 January 2013 and 90, 180, 270, and 360 days later.

Supplementary Table 1 | Deep mooring records in the Southern Ocean used in this study.

Mooring	LAT	LON	Depth (m)	Time period	Sampling interval	Source
WHOI 835	40.13°S	16.57°E	958	1985-02-26 to 1987-02-15	0.5 h	WHOI
SAZ47-2010	46.83°S	141.65°E	1100	2010-09-07 to 2011-08-15	0.5 h	IMOS/AODN
SAZ47-2015	46.83°S	141.65°E	1100	2015-03-21 to 2016-03-23	0.5 h	IMOS/AODN
Drake M7	58.96°S	58.10°W	980	2006-01-23 to 2007-12-13	24 h	SEANOE
