# Supplementary material for: Observed poleward heat transport by mesoscale eddies in the deep Southern Ocean
Source: Nat Commun. 2026 Aug 12;17:9664. doi: 10.1038/s41467-026-76674-2 (PMC13558553; doi:10.1038/s41467-026-76674-2)
Supplement: Supplementary file 2 [file 41467_2026_76674_MOESM2_ESM.pdf]

nature portfolio

Peer Review File

Observed poleward heat transport by mesoscale eddies in the deep Southern Ocean

Corresponding Author: Professor Tongya Liu

This file contains all reviewer reports in order by version, followed by all author rebuttals in order by version.

Version 0:

Reviewer comments:

Reviewer #1

(Remarks to the Author)
Enhanced poleward heat transport by mesoscale eddies in the deep Southern Ocean
The primary objective of this work is to quantify and map deep (1000 m) eddy kinetic energy and meridional eddy driven heat transport in the deep Southern Ocean as well as in the Southern Atlantic, Pacific and Indian Ocean Basins. The authors approach this by using the Scripps Argo Trajectory-based Velocity Product which uses the displacements of Argo floats at their parking depth to reconstruct velocity fields at 1000 meters. They find that, despite deep EKE being substantially reduced in the deep layers, rates of deep EMHT are of the same order of magnitude of that at the surface and intensified in the Indian Ocean sector. They substantiate their claim that deep mesoscale eddies are responsible for significant poleward heat transport using individual Argo trajectories at depth and comparing to drifters at the surface.
Overall, I appreciate the value in trying to observationally get at deep values of meridional heat transport, especially in a place as data sparse and climatically relevant as the Southern Ocean. However, I have some major concerns about the manuscript that will need addressing before publication. I think some very significant details and references are missing from the text. For example (more on this below), it is unclear to me how V’ is calculated from the Scripps product. I’m also not overly convinced that that these results are addressing any significant knowledge gaps as is. It is well understood that the mesoscale eddy field extends to depth and acts to move heat poleward in the Southern Ocean. Quantifying the magnitude of EMHT from observations is interesting however I think it’s possible these magnitudes are artificially inflated due to the smoothing that has to happen with the velocity product and/or the methodology used to get at V’. I think more work is needed to verify or corroborate results. I also found the tracer experiment to be a confusing addition to the manuscript. My specific concerns are listed below.

Major Comments:
Both the introduction and the methods section need a major overhaul and consider adding at least some information on data sources to the results section of the manuscript. There are a few mistakes in the intro and I think it needs more detail. Also I think you are missing some fundamental references that should be cited and in some places the reference that is cited seem inappropriate for the statement. I’m adding a few references at the end but consider a more complete literature search. As for the methods, after reading a number of times, it is still unclear to me how you got V’ values. At first I thought you were able to get V’ directly from the Scripps velocity product however I realized that is a time mean velocity so I assume you calculated your deviations from that using individual trajectories from Argo? This needs to be stated explicitly and in much more detail. If that is the case, I understand how you were able to separate stirring from trapping however I don’t feel as if this analysis added much to the manuscript. Consider removing or moving to supplementary.
I’m worried about the magnitude of EMHT that you calculated. I guess I just find it hard to believe that we would observe values at 1000 meters that are only half as large as at the surface. I worry that you are claiming a lot here without addressing the possibility that your values might be inflamed. For example, is the smoothing required for the Scripps product artificially reducing the magnitude of the deep velocity fields making the magnitude of your anomalies too large? I think it would be really helpful to see some additional estimates of MKE, EKE and EMHT from observations and from SOSE (if you keep in manuscript) at both the surface and at 1000 meters. Here is what I think you should include and compare the differences in magnitude and spatial pattern
• Surface MKE, EKE and EMHT from altimetry, SOSE and surface drifters. What if you subsample surface drifters to the sampling frequency (10 days) of Argo profiles, do you get the same magnitude of EMHT doing that?
• Deep MKE, EKE and EMHT from SOSE and Argo

The trajectory distribution of surface drifters and Argo floats (Fig. 3) is interesting however I have a few concerns. I would suggest making these into particle distribution maps rather than simply plotting trajectories. It feels a bit unpolished in current form. I'm also not convinced this is truly comparable to maps of EMHT. Couldn't the Argo floats just be a signature of southward interior flow? Also at the surface, you are going to have such a strong influence by the wind and Ekman that I don't know that you can claim this is concrete evidence that deep eddies are more efficient in poleward transport than in the surface layers. Also how do you reconcile that figure 2 suggests that north of the ACC EMHT is equatorward, especially in the Indian Ocean, but the floats are still going poleward? This at least needs to be addressed in the text. See comment below about how I think you can more effectively use the model to address this and directly compare to Lagrangian observations.
The tracer experiment felt a bit out of place with the rest of the study. I found it confusing and difficult to following and connect to the observations. I strongly suggest you 1) plot both surface and deep MKE, EKE and EMHT in the model and 2) consider replacing the tracer experiment with a particle release experiment. Then you could compare surface trajectories and deep trajectories like you do with the drifters and floats. I think you could probably still get at a zonally averaged longitudinal distribution of poleward heat transport from this to show that particles are preferentially crossing zonal jets at topographic ridges.

Minor Comments:
L29: I don't like the word underappreciated in this sentence. I don't think the role of mesoscale eddies in the Southern Ocean has been underappreciated at all.
L38 – 43: more detail here. For a reader who isn't familiar with the southern ocean this could be confusing.
L44: Eddies in the ACC don't move water from the subtropics to the subpolar
L49: does figure 1 show these? I don't really know what you mean by temperature anomalies. Expand here maybe
L50: remove 'from surface to the deep ocean'
L55: expand here
L56 – 58: this statement is false, along isopycnal upwelling transports deep water along density surfaces and facilitates ventilation. Eddy induced isopyncal mixing transports water across isopycnal surfaces.
Extended figures: consider which of these figures you actually need
L64 – 65: not just surface EKE. Missing a bunch of references here. Expand on this.
L69: I don't know what you mean by 'extreme temperature events'
L101 and fig. 1e: I'm confused by the eddy temperature anomalies and the plot in figure 1. What are they, what are you plotting and why are they a proxy for eddy intensity? What do you mean by composite analysis based on Argo? What mean are you subtracting from?
Fig 2: I think you are plotting EMHT in c/d/e but you need to specify that somewhere
L130: I think somewhere before this section you need to explain the difference between stirring and trapping of water by coherent vorticities/eddies
L150: I don't feel as if you have enough evidence to make this claim
L155: It doesn't look to me like temperature anomalies are all that large at 1000 meters compared to those at closer to 500 meters. I think what you are trying to claim here is that EMHT is dominated by the T' term? Maybe consider diagnosing each of the V'T' terms separately?
L157: This information feels like it comes too late. It's interesting that you are able to diagnose these eddy stirring vs. eddy trapping contributions separately. Results aren't surprising but it's an interesting piece to your analysis. Because it is unclear how V' values are calculated, it took me a long time to figure out how you separated between stirring and trapping. At first I thought you were using the velocity product to calculate EMHT but then I couldn't figure out how profiles were separated between stirring and trapping. More details are needed. Might consider adding some details about how many profiles are used in each calculation.
L179: While I believe this statement, I don't know that you've done enough to show us that Argo floats are preferentially crossing at bathymetric regions. At minimum I think you need to highlight where the topographic ridges are. Ideally, maybe you show a subset of trajectories that travel southward at these locations.
Fig 3k & 4g: It's interesting that more of the floats/tracer end up moving poleward and crossing the northern boundary of the ACC rather than the southern boundary. I would expect that we would see more poleward movement within the ACC itself. Would be interesting and add to your story to maybe breakdown the movement of floats between those that go southward vs. northward within the ACC
L262 – 264: rewrite these sentences. Confusing and seem contradictory

References:
Klocker et al. 2012, Reconciling float based and tracer based estimates of lateral diffusivities
Chapman and Sallée 2017 Isopyncal mixing suppression by the ACC and the Southern Ocean meridional overturning circulation
Phillips and Rintoul 2000 eddy variabitliy and energetics from direct current measurements in the ACC south of Australia
Roach et al. 2016 Horizontal mixing in the Sothern Ocean from Argo float trajectories
Abernathey and Cessi 2014, Topographic enhancement of eddy efficiency in baroclinic equilibration
Watts et al. 2016 estimates of eddy heat flux crossing the ACC from observations in Drake Passage
Ferrari and Nikurashin 2010, Suppression of eddy diffusivity across jets in the Southern Ocean
LaCase et al. 2017; Float derived isopycnal diffusivities in the DIMES experiment
Naveira Garabato et al. 2011, eddy stirring in the Southern Ocean
Balwada et al, 2016, Circulation and stirring in the southeast Pacific Ocean and Scotia Sea sectors of the ACC

Reviewer #2

(Remarks to the Author)
This manuscript addresses a long-standing and interesting question in Southern Ocean research by looking at eddy kinetic energy and meridional eddy heat transport using Argo data, in combination with analyses of surface drifters, satellite-based eddy locations, and model output from the Biogeochemical Southern Ocean State Estimate (B-SOSE). While the research questions are interesting, the manuscript has several shortcomings that suggest it is not ready for publication in its present form.

Overarching concerns:

1. the results are based on noisy observations but are presented without error bars or discussion of uncertainty. The authors draw conclusions about differences between ocean basins (Figure 1e), about mechanisms responsible for meridional heat transport (Figure 2b), about distinctions between surface and subsurface velocities (Figure 3c,f,i,j), and about eddy vs mean processes (Figure 4k), all without presenting uncertainty analyses to demonstrate that the curves are different in a statistically significant way.

2. The analysis has been presented without acknowledging an extensive body of previous work dating back several decades. This includes work on the role of eddy transport in the Southern Ocean (e.g. Johnson and Bryden, 1989), on the role of the Indian Ocean in warming the ACC (de Szoeke and Levine, 1981), eddy fluxes inferred from pre-cursors to Argo (Gille, 2003. Float observations of the Southern Ocean: Part 2, Eddy fluxes; subsequent work by a number of groups e.g. Sun et al, 2019, 10.1038/s41598-018-38069-2), and possible biases in such methods (Chinn and Gille, 2007, doi: 10.1175/JTECH2057.1). The lack of context makes it difficult to identify the novelty of the present work.

Specific comments:

3. In the introduction the discussion of eddy meridional heat flux has significant gaps. Previous authors have identified the role of eddies and the vertical structure of eddies. The authors should be clear about the distinctions between prior work and their new findings. I believe they are suggesting that their work puts forward an idea that regional temperature variations matter. This sounds useful, but in the end, I'm not sure that the authors have demonstrated how their work differs from heat budgets assembled by De Szoeke and Levine or from ocean inverse models.

4. Figure 1e. As noted above, the plot needs an uncertainty analysis. How robust are results? The implications are interesting but need to be demonstrated to be robust. Could the sector-by-sector findings be attributable to latitude ranges or distribution of data, or to the presence of the Kerguelen Plateau?

5. Figure 2b. As noted above, the figure cannot be interpreted without uncertainty estimates.

6. lines 149-151. The partitioning of the heat transport estimates does not appear justified. We usually think of the Antarctic Circumpolar Current as having an equivalent barotropic structure, with variability tapering with depth. It might make sense to incorporate this structure into an extraplation with depth. It does not seem credible to partition the ocean into a shallow surface layer and a uniform deep layer.

7. line 153. "surface poleward heat transport is suppressed": There is no reason to expect poleward heat transport at the surface in the Southern Ocean. Decades of analyses of meridional overturning circulation in the Southern Ocean have emphasized that surface transport is expected to take water northward, and the mid-depth flow should be poleward.

8. line 154. "large subsurface temperature anomalies": What is the evidence for subsurface temperature anomalies. This is something that requires figures and quantitative evidence.

9. lines 159-160. "Profiles persistently located within an eddy's interior for consecutive 10 days are attributed to eddy trapping": Since Argo floats profile every 10 days, and the Jason/Sentinel 6 series of altimeters measures every 10 days, a day-day threshold seems curiously short for this analysis. Maybe the text is not clearly worded. However, I think a longer time window would be appropriate, and if a 10-day window is the optimal choice, then the justification needs to be more clearly explained.

10. line 165. "likely stems from intense eddy-eddy and eddy-flow interactions": What evidence demonstrates that this is the likely explanation? Is this based on reference [31] or based on calculations carried out for this study? This needs to be explained in the manuscript.

11. lines 173-174. "We analyze the trajectories of Argo floats from four regions over a 1000-day period starting from January 1, 2013, ..." Argo floats are not designed to provide long trajectories, and the transition from ARGOS to Iridium led to substantial changes in the behavior of multi-cycle float trajectories. The text implies that consecutive displacements have been concatenated. Is that the case? Have they been screened to remove floats with long surface parking depths? These choices require more explanation.

12. lines 180-181. "Statistical results indicate that 17% of Argo floats cross the ACC northern boundary toward the pole, compared to only 7% of surface drifters, ..." I don't understand this sentence. Is this saying that floats move southward below the surface? That doesn't seem like a novel finding, given the bulk of previous literature. The quantitative results need to be reported with statistical uncertainties based on the standard error of the mean.

13. lines 181-182. "... Argo float trajectories demonstrating greater penetration of deep water than surface water into higher latitudes." These findings are not demonstrated by Figures 2 and 3. More evidence is needed if the authors wish to make this point.

14. line 184, reference to Fig. 3l. Comments about meridional movement are only meaningful if the authors can also show that the pdfs are statistically different. The distributions in Fig. 3l could result from differences in deployment locations or sparse sampling leading to non-robust statistics.

15. Figure 4. As noted above, at a minimum results in Figure 4k should include uncertainties, and ideally the maps should use cross-hatching to indicate non-statistically significant concentrations (or differences), and Figures 4i and 4j should show uncertainty in the ranges of distributions.

16. lines 227-230. "These results establish mesoscale eddies as critical conduits for deep subtropical-origin water intrusion into polar regions, with transport magnitude and efficiency reaching twice that of mean flow, underscoring their role in meridional heat and mass exchange in the deep Southern Ocean." This is not a profound result, given that the key role of eddies in governing Southern Ocean overturning circulation and heat transport has permeated the published literature since the 1980s. (See Johnson and Bryden, 1989, for example.)

17. lines 247-284. "The preceding Argo trajectory analysis indicates these warm intrusions originate primarily from the Indian Ocean." As noted above, the notion that the ACC warms in the Indian sector has permeated the published literature for many decades. This is not a novel resut and needs to be presented in the context of prior work (e.g. De Szoeke and Levine, 1981).

18. lines 461-462. "To evaluate the impact of high-frequency motions," The B-SOSE results reported here appear to differ from data based results by Chinn and Gille, 2007.

19. line 479 and following. The calculation of EMHT using contributions from coherent vortices needs further explanation. Eddy fluxes are normally computed assuming eddies to encompass all anomalies---not just coherent vortices. This separation needs to be justified more thoroughly.

20. lines 482-483. "it it remains within the same eddy for 10 days". Assuming that the authors do intend to distinguish eddy impacts due to coherent vortices and eddy variability due to everything else, the uncertainties of this separation need to be quantified. As noted above, 10 days is fairly short, particularly given the satellite orbits and the Argo surfacing times. Do some floats appear to enter and leave and then reenter the same eddies? How uncertain is this calculation?

21. Points of grammar/style:

a. line 67: "temperature" -> "temperatures that are"

b. line 72. "this process" -> "these processes"

c. line 74. "further": The text is unclear about what the authors investigate further than previous work investigated. I would suggest omitting the word.

Reviewer #3

(Remarks to the Author)
I read the manuscript “enhanced poleward heat transport by mesoscale eddies in the deep Southern Ocean” by Liu et al. with great interest. In this manuscript the authors use observations to show nicely that the eddy heat transport across the Southern Ocean is much larger relative to surface eddy heat transports than was previously assumed. These results are quite crucial to understand future changes in the Southern Ocean, in particular since models used for climate projections usually parameterise these processes, with parameterisations generally not able to represent regionally varying eddy transport of heat with unknown downstream impacts on the rest of the climate system.

The main issues I have here are regarding some of the text which has some room for improvement. In general, I think that these are small fixes that are quick to do, and I hope to see this manuscript published soon.

Here are my comments in order of appearance:

- title: I’m not sure I like the word “enhanced” in the title since it is unclear by reading just the title what the heat transport is enhanced relative to.

- line 38: “The prevailing westerly winds over this region drive the eastward-flowing ACC….this is quite outdated thinking!

One of the co-authors should surely know better (https://agupubs.onlinelibrary.wiley.com/doi/full/10.1029/2010GL044777). You get a strong ACC even in the limit of zero wind (e.g. https://journals.ametsoc.org/view/journals/phoc/43/3/jpo-d-12-095.1.xml, https://tellusjournal.org/articles/3231/files/6582dc3c97162.pdf, https://tellusjournal.org/articles/10.16993/tellusa.4107).

- line 61: "The magnitude of the EMHT is determined by both eddy activity and the background temperature field". This, and subsequent text latter in the manuscript, makes little mention of the effects of the mean flow, and then the authors then use words like "counterintuitive" or "surprising" to describe that eddy heat transport is so large at depth. The suppression of eddy mixing by mean flow at depth is now quite well-known (e.g. https://journals.ametsoc.org/view/journals/phoc/40/7/2010jpo4278.1.xml, https://journals.ametsoc.org/view/journals/phoc/42/9/jpo-d-11-0205.1.xml, https://elischolar.library.yale.edu/journal_of_marine_research/352/). So rather than explaining these results as so surprising, the authors should explain that according to theory/idealised experiments eddy mixing is dependent on EKE and mixing length, with the latter being modified by the mean flow. As a next step, the authors can then move on to the main point of the manuscript, showing that their observations are consistent with this theory, leading to enhance heat transport at depth.

- line 127-129: Are the acronyms "AC" and "EAC" really necessary for this little use in the manuscript??

- line 148-156: see comment on line 61; relate to the existing literature rather than pretend this is so counterintuitive. These results are actually quite logic, and that doesn't make them less exciting.

- line 157-166: Is this paragraph really needed in the manuscript? I'm not convinced it adds anything.

- line 200-202: I'm not sure why the mean flow experiments are necessary. Of course there is almost no meridional flow here. And if wanting to "..evaluate the role of eddies…" why not show eddy-only simulations (such as https://elischolar.library.yale.edu/journal_of_marine_research/352/) or at least compare to previous results.

I hope this helps to improve your manuscript a bit!

Version 1:

Reviewer comments:

Reviewer #1

(Remarks to the Author)
I would like to thank the authors for their careful and complete resubmission. They have significantly improved this manuscript, and I believe it is just about ready for publication after a few minor edits.
Paragraph starting at L116: After removing the eddy trapping/stirring section, this paragraph/analysis feels a bit out of place and confusing. Consider removing from here and the methods section (L390).
L169: I feel like this sentence needs a bit more. Explain what the theoretical framework of eddy mixing suppression is.
L176: I thought the SOSE validation was a great addition, nice job!
Fig 3: can the colorbar be moved off the figure, like in figure 4? Also I assume the values are negative because these are exponents? Maybe just make that clear in the colorbar label. Same goes for colorbar on fig 4
L271: I know what you mean when you say 'without external replenishment' but this phrasing is a bit weird. That water moving southward obviously has to be replaced somehow. Is it upwelling? Is it possible that cooler waters are being moved northward?
L273: I'm not exactly sure which part of the analysis you are pointing to when you say 'preceding Argo trajectory analysis further…' sentence is just a bit confusing. Revise.
L297: I find this sentence a bit misleading, you have shown that mean EMHT at depth is about half that at the surface. You noted that at depth, it can reach values of that at the surface but I took that to mean instantaneous deep EMHT can reach values of mean EMHT at surface.
Section starting at L403: I'm sorry to harp on this, I think I understand but I just want you to be explicitly clear on how you arrived at your values of u' and v'. Do you get them directly from the Argo Trajectory based velocity product? Meaning, does this product have a time dimension? I was under the impression that the Argo product produced a time mean velocity field, but I could absolutely be wrong about that. If not, I assume the prime quantities were calculated directly by you from individual trajectories?
L433: remove 'at' at the end of this sentence I think?
The table at the end is helpful, thank you. Nice edits!

Reviewer #2

(Remarks to the Author)
In this revised manuscript, the authors have made a significant effort to address reviewer comments. I appreciate their efforts to provide greater clarity and better citation of previous work in this area. I continue to have several concerns that need to be addressed. At a basic level, the authors have not met fundamental expectations for reproducible science by providing uncertainty estimates and by documenting known challenges associated with the data used for the study.

1. Line 89 and following. Resolution and high-resolution performance of the Biogeochemical version of SOSE. In the results discussion of Biogeochemical-SOSE, it would be useful to indicate the resolution of the version being used here. (Note that by convention, the developers of the BGC version of SOSE refer to it as "B-SOSE".) More importantly, in the methods section, there seem to be gaps in the information about the fidelity of the state estimate fields at high wavenumber. Is the spectral slope realistic for scales shorter than 50 km? Are eddies realistic? Supplementary Fig. 2 shows spectra from SOSE, but it would be useful to compare high-wavenumber and/or high-frequency variability in the model with mooring data or with an equivalent assessment of a higher-resolution forward model (such as llc4320), or other independent information, to back up the assertion that motions with scales less than 10 days account for only about 1% of kinetic energy. The drop-off in energy in Supplementary Fig. 2 for temporal scales shorter than about 25 days is consistent with behavior that might result from the model's suppression of energy at scales shorter than about 4 times the grid spacing. This could suggest that the model behavior is governed by imposed diffusivity, which could have distinctly different physics than the eddies in the ocean.

2. Figure 2 caption. I appreciate the inclusion of shaded uncertainty ranges in panel b, but I do not understand why the surface values do not include an uncertainty range. The surface values should be reported with an uncertainty.

3. Line 163. The maximum and mean EMHT values should be reported with uncertainty estimates. It's worth noting that the mean surface transport of -0.71 TW/m represents a mean of a highly variable quantity. Typically, readers expect mean quantities to be reported along with a standard error of the mean.

4. Figure 3 shows trajectories from Argo floats over 1000 days. As my previous review mentioned, Argo trajectories are not meaningful representations of 1000-day motions because the floats move much faster near the surface than they do at 1000 m. The reply to reviewers indicates that the authors have not fully grasped this point. They write, "Once a drifter or float first enters the prescribed source region, we follow its subsequent trajectory for up to 1,000 days, if such records are available." Let me be clear about this. In order to transmit data, the older pre-Iridium floats spent as much as 24 hours at the surface during each 10-day cycle. An estimated 50% of net displacement occurred at the surface. Thus the 1000-day trajectories are as much an indicator of surface motion as 1000-m motion. Iridium floats avoid this challenge for the most part, but they still are imperfect Lagrangian particles, and their trajectories should be interpreted with caution. Caveats about the trajectories appear to be missing from the manuscript.

5. With reference to my recommendation to add error bars to Figure 4, the authors wrote in their response document, "For a given velocity field and initial tracer concentration, the tracer distribution is deterministic. Therefore, statistical uncertainty or statistical significance is not directly defined for this figure." This comment suggests that the authors have not fully thought out the nature of a random variable. Although they have only one realization of the model, we can hypothesize the possibility of an ensemble of realizations, or an ensemble of particle release experiments, all with slightly different values. Uncertainty estimates are important as a means for assessing robustness for these values. Small perturbations in release data and small perturbations in velocity would imply differences in distribution that should be assessed.

6. Experiments 5 and 6 are compared in Figure 5. Notably, Fig 5c reports heat flux in units of J/m as function of time. It's not clear if the average temperature gradient is the same in Experiments 5 and 6. Since the results are reported in units of J/m (rather than as an effective diffusivity), the net heat transport is expected to vary with the magnitude of the temperature gradient. More detail is needed to demonstrate that the plotted results have any useful meaning.

7. Line 366. The drifters are reported as representing velocities at a nominal depth of 15 m. It would be useful if the methods section clarified whether drifters are excluded when they lose their drogues.

8. Supplementary Fig. 2. The caption is not clear about the data source for this figure.

9. Supplementary Fig. 3. The caption is not clear about whether this figure is based on observed floats and drifters or on particle release experiments using SOSE fields. The caption should be rewritten for clarity. If SOSE fields are used, then clarity should be provided about differences between SOSE and particle means.

10. Supplementary Fig. 4. Mean anomalies should be reported with uncertainties representing the standard error of the mean.

11. Supplementary Fig. 5. The red lines indicating SOSE values are shown without uncertainty estimates. It's not clear why uncertainties are omitted. Does SOSE provide a fully closed budget?

12. Supplementary Fig. 5. "grey shading indicates the standard deviation from 50 random subsamples". This wording is ambiguous. Is the standard deviation dependent on the number of elements included in each subsample? Are the number of samples scaled to match the number of Argo floats used for this analysis?

13. Supplementary Fig. 6. It's important to reiterate that 1000-day Arog trajectories are not meaningful because Argo floats are not designed as Lagrangian particles, and in particular pre-Iridum Argo floats experience a major fraction of their total motion at the surface.

14. Grammar and style
a. Line 44. "deeply (> 1500 m)" would be clearer as "to depths greater than 1500 m"

b. Line 108. “that” -> “their role”
c. Line 131. I would remove “further”, since the opening clause of the sentence clarifies the temporal sequence.
d. Line 132. “particle-based estimates”. Please clarify that this refers to “SOSE-derived particle-based estimates”.
e. Line 452-454. “The number of samples obtained from drifters (Argo floats) in the Indian, Pacific, Atlantic Ocean sectors, and ACC region is 1062 (737), 594 (610), 749 (484), and 2427 (1973), respectively”. The use of contrasting examples in parentheses makes the text difficult for readers to interpret. It would be worth putting the information in a table, or else stretching out the sentence to be clearer about the drifter vs Argo examples.

Reviewer #3

(Remarks to the Author)
I'm happy with the changes the authors made in response to my comments, and hope to see this nice manuscript published soon!

Version 2:

Reviewer comments:

Reviewer #2

(Remarks to the Author)
The authors have provided a careful response to the issues that I raised in the most recent round of reviews, and I think this manuscript now meets the standards for publication.

Open Access This Peer Review File is licensed under a Creative Commons Attribution 4.0 International License, which permits use, sharing, adaptation, distribution and reproduction in any medium or format, as long as you give appropriate credit to the original author(s) and the source, provide a link to the Creative Commons license, and indicate if changes were made.
In cases where reviewers are anonymous, credit should be given to 'Anonymous Referee' and the source.
The images or other third party material in this Peer Review File are included in the article’s Creative Commons license, unless indicated otherwise in a credit line to the material. If material is not included in the article’s Creative Commons license and your intended use is not permitted by statutory regulation or exceeds the permitted use, you will need to obtain permission directly from the copyright holder.
To view a copy of this license, visit https://creativecommons.org/licenses/by/4.0/

Responses to the reviewers

General Response

We would like to thank the three anonymous reviewers for their constructive comments on our manuscript, which have significantly helped us to improve the quality of this work. Based on these valuable suggestions, we have performed a comprehensive revision of the manuscript.

The major modifications are summarized as follows:

- **Method Validation**: We add new particle-tracking experiments to test the reliability of estimating deep EKE and EMHT from discrete observational samples. Using SOSE velocity fields, we show that the discrete sampling approach can robustly reproduce EKE and EMHT in the deep Southern Ocean.
- **Novelty**: We have thoroughly updated the literature review with foundational references. We now clarify the main novelty of our study: it is the first to quantify deep EKE, and EMHT based on observational data, and to systematically assess the distinct contributions of different ocean basins to deep EMHT in the Southern Ocean.
- **Textual Improvements**: We have substantially reorganized the Introduction and refined the textual descriptions throughout the manuscript to improve clarity, precision, and logical flow.
- **Sharper Focus**: We have removed the previous section on eddy trapping and stirring to keep the manuscript focused on deep EKE, EMHT and basin-scale heat redistribution.

We provide a detailed, point-by-point response to each reviewer’s comment below, with the reviewers' comments shown in blue and our responses in black.

Reviewer #1

The primary objective of this work is to quantify and map deep (1000 m) eddy kinetic energy and meridional eddy driven heat transport in the deep Southern Ocean as well as in the Southern Atlantic, Pacific and Indian Ocean Basins. The authors approach this by using the Scripps Argo Trajectory-based Velocity Product which uses the displacements of Argo floats at their parking depth to reconstruct velocity fields at 1000 meters. They find that, despite deep EKE being substantially reduced in the deep layers, rates of deep EMHT are of the same order of magnitude of that at the surface and intensified in the Indian Ocean sector. They substantiate their claim that deep mesoscale eddies are responsible for significant poleward heat transport using individual Argo trajectories at depth and comparing to drifters at the surface.

Overall, I appreciate the value in trying to observationally get at deep values of meridional heat transport, especially in a place as data sparse and climatically relevant as the Southern Ocean. However, I have some major concerns about the manuscript that will need addressing before publication. I think some very significant details and references are missing from the text. For example (more on this below), it is unclear to me how V' is calculated from the Scripps product. I'm also not overly convinced that that these results are addressing any significant knowledge gaps as is. It is well understood that the mesoscale eddy field extends to depth and acts to move heat poleward in the Southern Ocean. Quantifying the magnitude of EMHT from observations is interesting however I think it's possible these magnitudes are artificially inflated due to the smoothing that has to happen with the velocity product and/or the methodology used to get at V'. I think more work is needed to verify or corroborate results. I also found the tracer experiment to be a confusing addition to the manuscript. My specific concerns are listed below.

Response: We thank the reviewer for the careful reading of our manuscript and for the constructive comments. We apologize for the lack of clarity in the original version. The manuscript has been revised accordingly, and the concerns raised have been addressed. Detailed, point-by-point responses are provided below.

Major Comments

R1-1 Both the introduction and the methods section need a major overhaul and consider adding at least some information on data sources to the results section of the manuscript. There are a few mistakes in the intro and I

think it needs more detail. Also I think you are missing some fundamental references that should be cited and in some places the reference that is cited seem inappropriate for the statement. I'm adding a few references at the end but consider a more complete literature search. As for the methods, after reading a number of times, it is still unclear to me how you got V' values. At first I thought you were able to get V' directly from the Scripps velocity product however I realized that is a time mean velocity so I assume you calculated your deviations from that using individual trajectories from Argo? This needs to be stated explicitly and in much more detail. If that is the case, I understand how you were able to separate stirring from trapping however I don't feel as if this analysis added much to the manuscript. Consider removing or moving to supplementary.

Response: We thank the reviewer for these constructive comments. We have substantially revised the Introduction to correct errors and provide more details. We have incorporated the key references suggested by the reviewer together with relevant recent literature (Lines 38-72). We have also added information on the data sources in the Results section for clarity (Lines 84-94).

Regarding the calculation of v' values, we have clarified the procedure in the Methods section (Lines 423-427). Since high-frequency (<10 days) motions are negligible at 1000 m (Supplementary Fig. 2), the 10-day Argo displacement velocity retains most of the velocity variability at this depth. The velocity anomaly is defined relative to the local ensemble mean as $u' = u - \bar{u}$, where $\bar{u}$ is the mean velocity calculated from all Argo samples within each 2°×1° grid cell. The same procedure is applied to the meridional velocity v to obtain v'. Similar approaches have been applied to earlier sparse float observations by Gille (2003) and Chinn and Gille (2007), and we have added these references in Lines 432-440. Following the reviewer's suggestion, we have also verified our results using SOSE data (see our response to R1-2).

To maintain focus on the main results, the analysis of stirring and trapping has been removed. This analysis was originally considered because it connects to our previous work on eddy trapping and stirring, but it is not central to the objectives of the present study.

R1-2 I'm worried about the magnitude of EMHT that you calculated. I guess I just find it hard to believe that we would observe values at 1000 meters that are only half as large as at the surface. I worry that you are claiming a lot here without addressing the possibility that your values might be inflamed. For example, is the smoothing required for the Scripps product artificially reducing the magnitude of the deep velocity fields making the

magnitude of your anomalies too large? I think it would be really helpful to see some additional estimates of MKE, EKE and EMHT from observations and from SOSE (if you keep in manuscript) at both the surface and at 1000 meters. Here is what I think you should include and compare the differences in magnitude and spatial pattern.

• Surface MKE, EKE and EMHT from altimetry, SOSE and surface drifters. What if you subsample surface drifters to the sampling frequency (10 days) of Argo profiles, do you get the same magnitude of EMHT doing that?

• Deep MKE, EKE and EMHT from SOSE and Argo

Response: Based on the reviewer's suggestion, we have conducted a comprehensive validation of our core calculation methodology. The results demonstrate that the deep EKE and EMHT calculated using discrete Argo data are not overestimated. In fact, these values are likely underestimated in the real ocean.

Specifically, we use the surface and 1000 m daily velocity fields from the SOSE to drive Lagrangian particles. The particles are initialized at a spatial resolution of 0.4°×0.2°, totaling over 160,000 particles, and are integrated continuously from January 1, 2013, to December 31, 2017. By calculating the zonal and meridional velocities based on particle positions every 10 days, we obtain a total of 29 million velocity samples, which far exceeds the approximately 550,000 samples in the Argo velocity product. For a fair comparison, we randomly subsample this dataset to match the exact number of observational samples. We then use these subsamples to calculate the MKE, EKE, and EMHT at both the surface and 1000 m. To ensure statistical reliability, this random subsampling process is **repeated 50 times**.

Here, we treat the Eulerian results calculated directly from SOSE as the true values to evaluate how well the particle-based method reproduces them. Supplementary Fig. 3 shows that the particle-based method captures the spatial patterns and magnitudes of MKE at both the surface and 1000 m. This method also performs well for deep EKE and EMHT, with spatial correlations of 0.92 and 0.80, respectively, although their magnitudes are slightly underestimated (Supplementary Fig. 3 and Supplementary Fig. 5). This bias is expected because the particle-based velocities are 10-day displacement velocities rather than instantaneous velocities. Although the SOSE energy spectrum shows that motions with periods shorter than 10 days are weak at 1000 m, displacement velocities still smooth part of the true velocity variability. In addition, when calculating the temperature anomaly $T' = T - \overline{T}$ for EMHT, T is taken as the temperature averaged along the same 10-day trajectory segment, rather

than an instantaneous value, to remain consistent with the drift-temperature information available in the Argo dataset. This validation therefore demonstrates that the particle-based method captures the climatological-scale patterns of deep EKE and EMHT, supporting its application to Argo observations.

We also note that this method substantially underestimates surface EKE and EMHT. This is because high-frequency motions at the surface are not negligible (Supplementary Fig. 2), so 10-day displacement velocities are not suitable for estimating surface EKE or EMHT. For this reason, we do not use surface drifter observations to diagnose surface EKE or EMHT in this study. Instead, surface estimates are obtained from satellite observations.

Finally, SOSE itself does not reproduce the strong deep EMHT estimated from Argo observations. This discrepancy likely arises because SOSE underestimates the temperature anomalies of mesoscale eddies at depth. Eddy-composite analysis shows that, in the Indian Ocean sector, the temperature anomalies of both cyclonic and anticyclonic eddies in SOSE are only about half of those derived from Argo profiles (Supplementary Fig. 4 in R1-14). This result further highlights the importance of accurately representing the deep thermal structure of mesoscale eddies in models. These revisions are included in Lines 83-96, Lines 130-139, Lines 175-184, Lines 457-470.

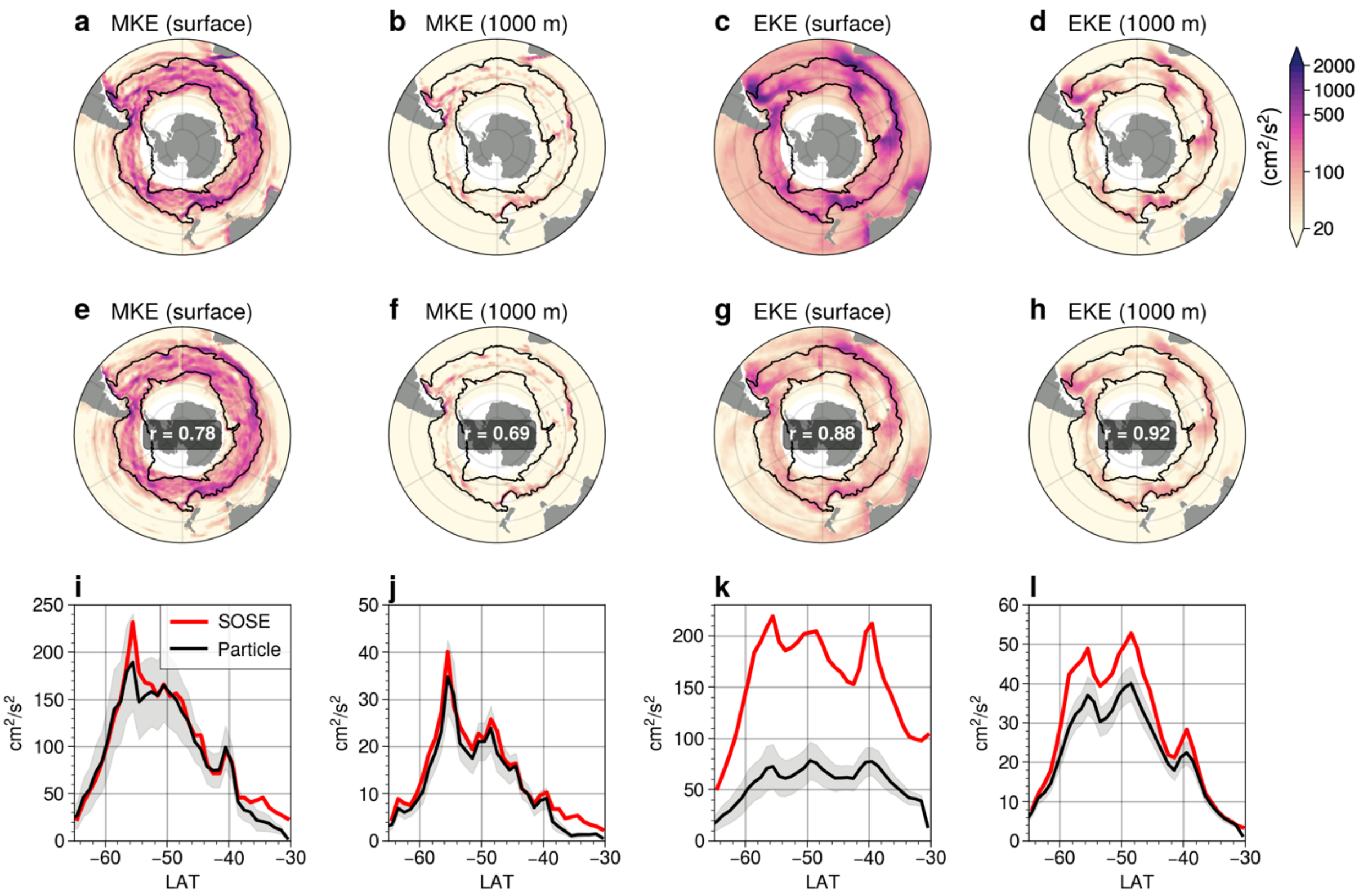

Supplementary Fig. 3 | Validation of particle-based MKE and EKE against SOSE. Surface MKE (**a**), MKE at 1000 m (**b**), surface EKE (**c**), and EKE at 1000 m (**d**) from SOSE. Particle-based estimates of surface MKE (**e**), MKE at 1000 m (**f**), surface EKE (**g**), and EKE at 1000 m (**h**). Numbers in **e–h** indicate the spatial correlation with the corresponding SOSE field. Zonal-mean comparisons of surface MKE (**i**), MKE at 1000 m (**j**), surface EKE (**k**), and EKE at 1000 m (**l**). Red lines denote the SOSE estimates, black lines denote the particle-based estimates, and grey shading indicates the standard deviation from 50 random subsamples.

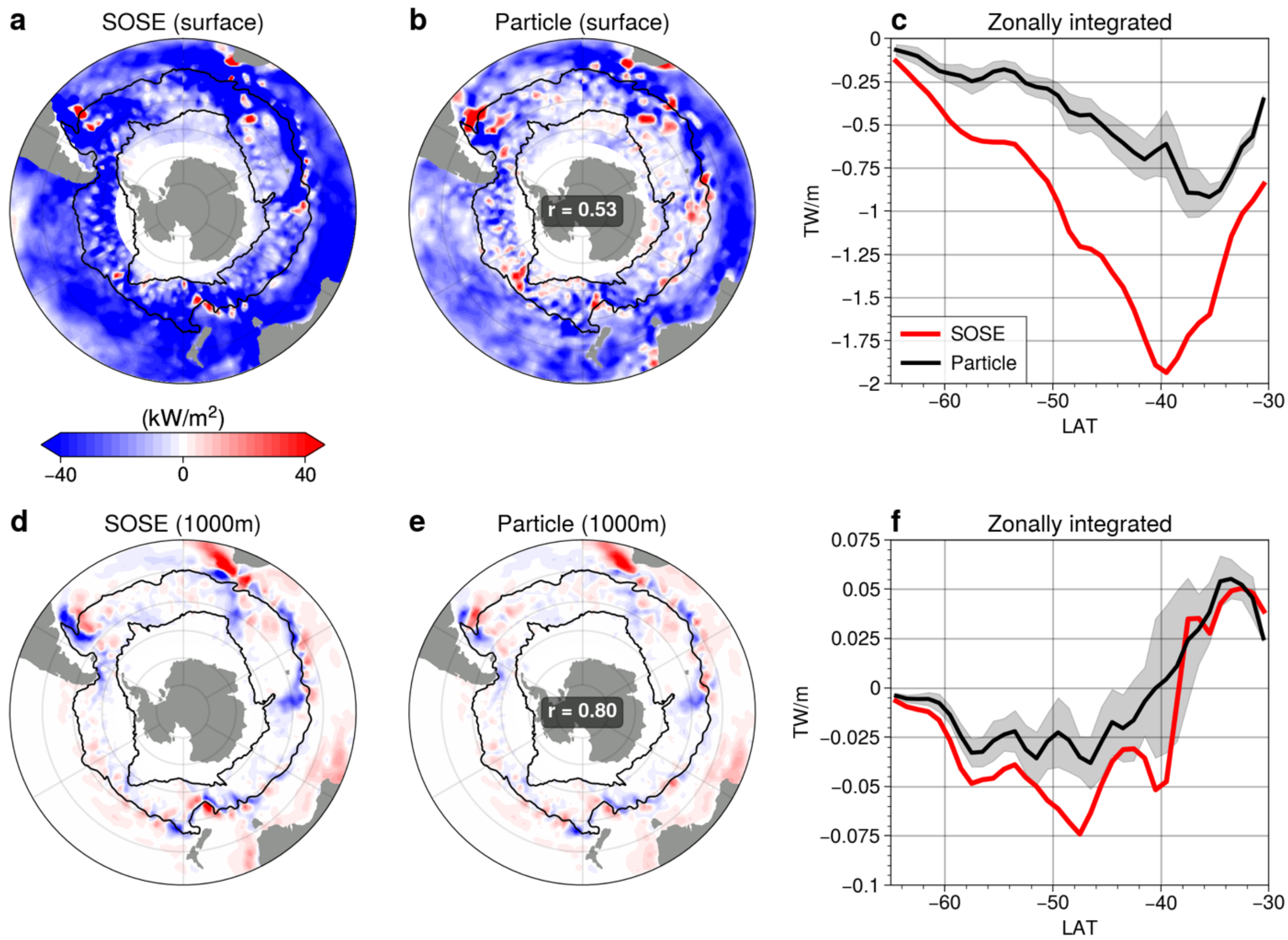

Supplementary Fig. 5 | Validation of particle-based EMHT estimates against SOSE. Surface EMHT from SOSE (**a**) and the particle-based estimate (**b**). Zonal-mean comparison of surface EMHT (**c**). EMHT at 1000 m from SOSE (**d**) and the particle-based estimate (**e**). Zonal-mean comparison of EMHT at 1000 m (**f**). Numbers in **b** and **e** indicate the spatial correlation with the corresponding SOSE field. In **c** and **f**, red lines denote the SOSE estimates, black lines denote the particle-based estimates, and grey shading indicates the standard deviation from 50 random subsamples.

R1-3 The trajectory distribution of surface drifters and Argo floats (Fig. 3) is interesting however I have a few concerns. I would suggest making these into particle distribution maps rather than simply plotting trajectories. It feels a bit unpolished in current form. I'm also not convinced this is truly comparable to maps of EMHT. Couldn't the Argo floats just be a signature of southward interior flow? Also at the surface, you are going to have such a strong influence by the wind and Ekman that I don't know that you can claim this is concrete evidence that deep eddies are more efficient in poleward transport than in the surface layers. Also how do you reconcile that figure 2 suggests that north of the ACC EMHT is equatorward, especially in the Indian Ocean, but the floats are still going poleward? This at least needs to be addressed in the text. See comment below about how I think you can more effectively use the model to address this and directly compare to Lagrangian observations.

The tracer experiment felt a bit out of place with the rest of the study. I found it confusing and difficult to following and connect to the observations. I strongly suggest you 1) plot both surface and deep MKE, EKE and EMHT in the model and 2) consider replacing the tracer experiment with a particle release experiment. Then you could compare surface trajectories and deep trajectories like you do with the drifters and floats. I think you could probably still get at a zonally averaged longitudinal distribution of poleward heat transport from this to show that particles are preferentially crossing zonal jets at topographic ridges.

Response: Following the reviewer's suggestion, we have replaced the trajectory plots with spatial probability distribution maps (Fig. 3). Additionally, we incorporate the trajectory probability distributions of SOSE-driven particles into this figure. To eliminate potential biases caused by varying initial sampling locations (a concern raised by Reviewer 2), we strictly confine the initial positions in the Indian, Pacific, and Atlantic Ocean sectors to a narrow region within 3° north of the ACC.

The results clearly demonstrate that surface drifters tend to move equatorward, whereas deep Argo floats move poleward, consistent with our previous arguments. Furthermore, when randomly subsampling an equal number of SOSE-driven particles, the simulated trajectories exhibit an even more pronounced divergence in these opposite directions. This clearer signal occurs because simulated particles maintain a continuous 1000-day tracking period, whereas the tracking durations of real observational floats may not always reach 1000 days (also see R2-11). The standard deviation derived from the 50 random resampling iterations is small, confirming the robustness of this contrasting spatial pattern.

We agree that drifter and float trajectories alone cannot be directly equated to the spatial structure of EMHT. Trajectories are influenced by the full flow, including both mean flow and eddies. This is why we design the tracer experiment. The major advantage of the tracer approach is its ability to explicitly separate the contributions of mesoscale eddies from those of the mean flow. This decomposition clearly demonstrates that the deep poleward heat transport is predominantly driven by eddies, while also allowing us to quantify the specific contributions and spatial influence ranges of different ocean basins. Therefore, we prefer to retain the tracer experiment in the manuscript, as it fundamentally supports and cross-validates the Lagrangian particle results.

Regarding your suggestion to plot both the surface and deep MKE, EKE, and EMHT in the model, please refer to our response to Comment R1-2. In addition, we apologize for the unclear description regarding the apparent contradiction north of the ACC, where EMHT is equatorward while floats appear to move poleward. Our previous statistic showing that 17% of Argo floats cross the ACC poleward was calculated over a broad domain encompassing many regions far away from the ACC. Therefore, it does not imply that floats in those specific northern regions are actively moving poleward. Meanwhile, the equatorward EMHT north of the ACC is fundamentally dictated by the local background temperature gradient (see Fig. 2c). All descriptions related to this part have been revised. Please see Lines 198-217 and Lines 446-455.

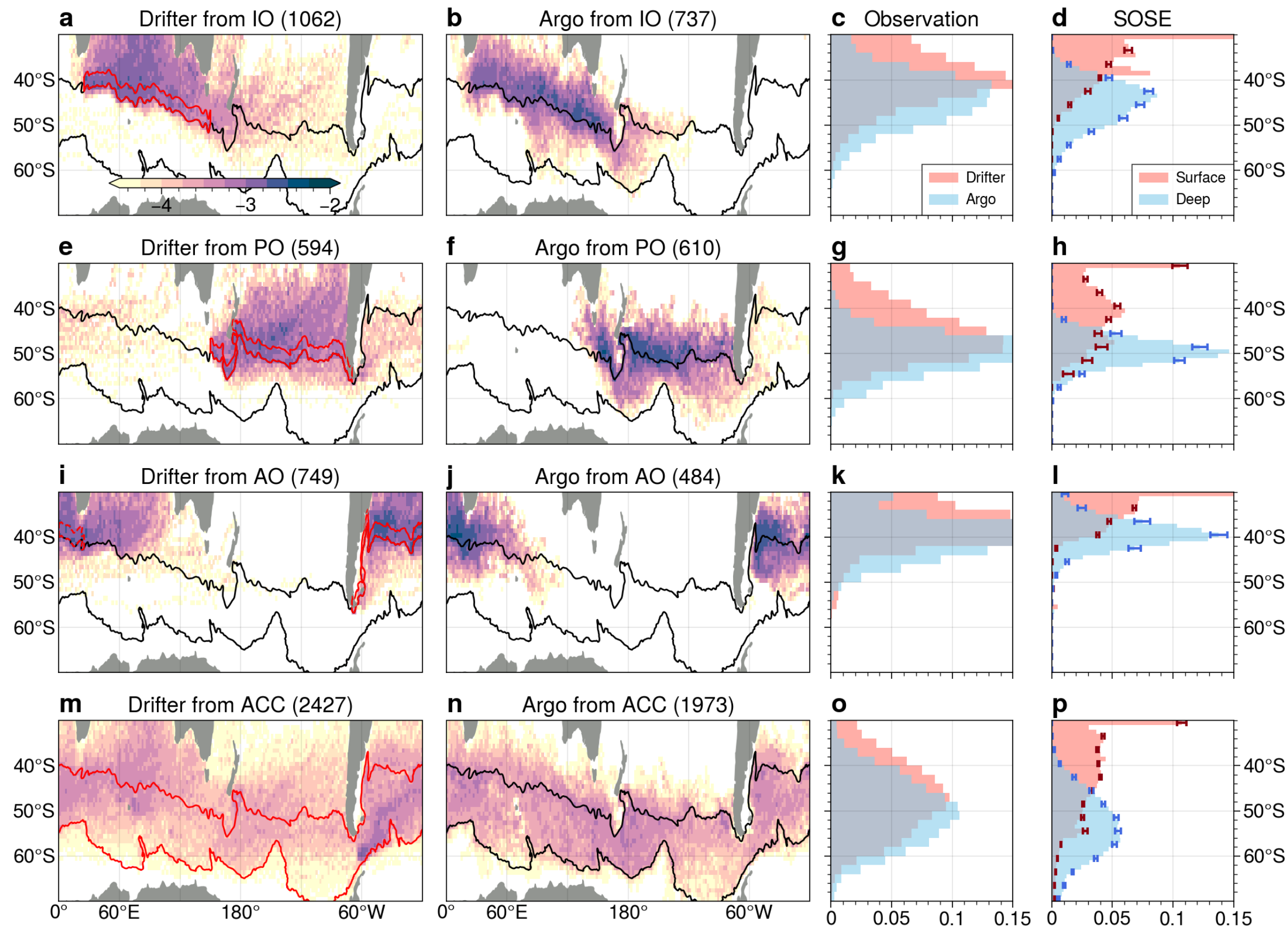

Fig. 3 | Spatial and latitudinal probability distributions of surface drifters, Argo floats, and SOSE particles in the Southern Ocean. a–d, Spatial probability distributions of surface drifters (**a**) and Argo floats (**b**) from the Indian Ocean sector over 1,000 days, along with the latitudinal probability distributions of the observations (**c**) and SOSE-driven particles (**d**). **e–h**, from the Pacific Ocean sector. **i–l**, from the Atlantic Ocean sector. **m–p**, from the entire ACC region. Numbers in parentheses indicate total counts for each region. Red lines in the first column outline the initial positions of all observations and model particles. Error bars in the fourth column represent the standard deviation of 50 random subsamples. Black lines mark the northern and southern boundaries of the ACC.

Minor Comments:

R1-4 L29: I don't like the word underappreciated in this sentence. I don't think the role of mesoscale eddies in the Southern Ocean has been underappreciated at all.

Response: We agree that "underappreciated" is too strong in this context. Our intention was to emphasize that this study provides observational evidence for deep EMHT. We have therefore revised the sentence to: "These

findings provide new observational evidence for the role of mesoscale eddies in modulating deep Southern Ocean heat redistribution and their potential impact on Antarctic climate". (Lines 29-31)

R1-5 L38 – 43: more detail here. For a reader who isn't familiar with the southern ocean this could be confusing.

Response: We have reorganized the entire Introduction, and the first paragraph has been revised accordingly with additional details to improve clarity for readers less familiar with the Southern Ocean (Lines 38-46).

R1-6 L44: Eddies in the ACC don't move water from the subtropics to the subpolar

Response: We apologize for the unclear statement. Our intention was to describe the poleward redistribution of heat, rather than the direct transport of water from the subtropics to the subpolar Southern Ocean. We have revised this sentence accordingly (Lines 44-46).

R1-7 L49: does figure 1 show these? I don't really know what you mean by temperature anomalies. Expand here maybe

Response: We have revised this part for clarity. The composite vertical structure of mesoscale eddy temperature anomalies is now shown in Supplementary Fig. 4 (R1-14). Temperature anomalies are defined relative to the monthly climatological mean field, and are used here as a useful thermodynamic proxy for the strength and vertical extent of mesoscale eddies. See Lines 390-401.

R1-8 L50: remove 'from surface to the deep ocean'

Response: This sentence has been removed in the revised manuscript.

R1-9 L55: expand here

Response: We have revised this part and added the relevant references on the suppression of eddy mixing by the mean flow. The corresponding text has been updated accordingly (Lines 54-58).

R1-10 L56 – 58: this statement is false, along isopycnal upwelling transports deep water along density surfaces and facilitates ventilation. Eddy induced isopyncal mixing transports water across isopycnal surfaces.

Response: We respectfully disagree with that last part of this point. Eddy induced isopycnal mixing transports tracers along isopycnal surfaces and can also contribute to ventilation (see Morrison et al. 2022 for a review of this process). Mixing across isopycnal surfaces is referred to as diapycnal mixing. However, the original statement has been removed from the Introduction during our revision. We now discuss this issue in the Discussion section. See Lines 308-313.

"It is necessary to note that data scarcity restricts the direct quantification of EMHT to the standard Argo parking depth (1000 m), whereas heat redistribution in the Southern Ocean is inherently a three-dimensional process (Supplementary Fig. 9). Once subtropical warm water is advected across the ACC in the deep Southern Ocean, it is upwelled toward the upper ocean through two mechanisms: isopycnal transport and mixing along tilted density surfaces[25,39,40] and diapycnal mixing across weakly stratified layers in the region south of the ACC[41]."

R1-11 Extended figures: consider which of these figures you actually need

Response: We have reorganized the Supplementary figures and retained only those necessary to support the main results.

R1-12 L64 – 65: not just surface EKE. Missing a bunch of references here. Expand on this.

Response: We have revised this part and added the relevant references (Lines 64-65).

R1-13 L69: I don't know what you mean by 'extreme temperature events'

Response: "Extreme temperature events" is commonly used in climatology, for example in the context of marine heatwaves. Here, our intention was to emphasize the role of subsurface eddies. To avoid confusion, we have removed this expression from the revised manuscript and added the discussion of subsurface eddies in Lines 319-320.

R1-14 L101 and fig. 1e: I'm confused by the eddy temperature anomalies and the plot in figure 1. What are they, what are you plotting and why are they a proxy for eddy intensity? What do you mean by composite analysis based on Argo? What mean are you subtracting from?

Response: In the revised manuscript, this figure has been moved from Fig. 1e to the Supplementary Fig. 4. Our intention was to illustrate the vertical structure of temperature anomalies within mesoscale eddies. Because direct velocity observations in the deep ocean remain sparse, these eddy-associated temperature anomalies can provide a useful thermodynamic proxy for the strength and vertical extent of mesoscale eddies. We have also clarified that the composite analysis follows the general Argo–eddy compositing approach used in previous studies (e.g., Frenger et al., 2015). Argo profiles collocated around detected eddies are projected into an eddy-centered coordinate system normalized by eddy radius. Temperature anomalies are defined relative to the monthly climatological mean field. The corresponding explanation has been added to the Methods section, and the text has been revised accordingly (Lines 390-401).

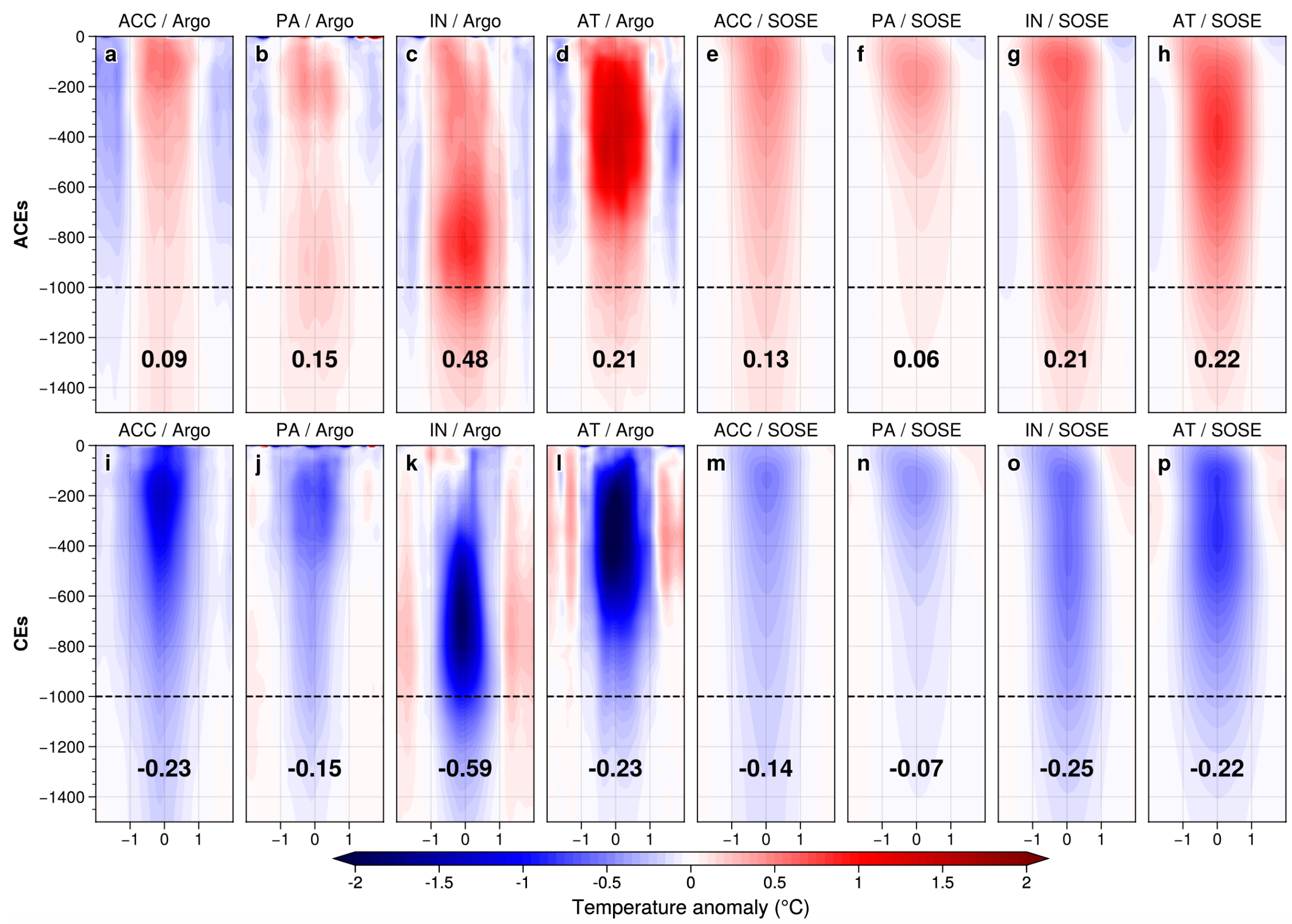

Supplementary Fig. 4 | Composite vertical structure of mesoscale eddy temperature anomalies from Argo and SOSE. a–d, Composite temperature anomalies for anticyclonic eddies derived from Argo in the ACC (**a**), Pacific (**b**), Indian (**c**), and Atlantic (**d**) sectors. **e–h,** Same as **a–d**, but derived from SOSE. **i–l,** Composite temperature anomalies for cyclonic eddies derived from Argo in the ACC (**i**), Pacific (**j**), Indian (**k**), and Atlantic (**l**) sectors. **m–p,** Same as **i–l**, but derived from SOSE. Numbers in each panel indicate the mean temperature anomaly within one normalized eddy radius of the eddy center. Temperature anomalies are calculated relative

to the monthly climatological mean field.

R1-15 Fig 2: I think you are plotting EMHT in c/d/e but you need to specify that somewhere.

Response: We have reorganized Fig. 2 in the revised manuscript. The original panels related to stirring and trapping (Fig. 2c-e) have been removed. The revised figure now includes the temperature gradient at 1000 m and the ratio of deep EMHT to surface EMHT.

R1-16 L130: I think somewhere before this section you need to explain the difference between stirring and trapping of water by coherent vorticities/eddies

Response: We have removed this part from the revised manuscript, as it is not central to the main focus of this study.

R1-17 L150: I don't feel as if you have enough evidence to make this claim

Response: We agree that our previous division between the "surface" and "deep" layers was not sufficiently rigorous to support the original claim. We have therefore revised the analysis to compare only the surface estimate with the EMHT at 1000 m, and have modified the text accordingly. In the revised Fig. 2, we show that although the zonally integrated EMHT at 1000 m is weaker than the surface estimate, its regional magnitude can be comparable to, or locally exceed, the surface signal, particularly along the northern boundary of the ACC in the Indian sector. See Lines 166-169.

R1-18 L155: It doesn't look to me like temperature anomalies are all that large at 1000 meters compared to those at closer to 500 meters. I think what you are trying to claim here is that EMHT is dominated by the T' term? Maybe consider diagnosing each of the V'T' terms separately?

Response: The original discussion was not sufficiently precise in describing the vertical structure of temperature anomalies and their contribution to EMHT. In the revised manuscript, we no longer broadly discuss the 500–1500 m range as a single "deep" layer, and instead focus more explicitly on the comparison between the surface and 1000 m.

We have clarified that the distinct spatial pattern of EMHT at 1000 m, relative to the surface, is largely shaped by the meridional temperature gradient (dT/dy). Our composite analysis also confirms that mesoscale eddies retain pronounced temperature anomalies at 1000 m. To further examine the role of the temperature term, we add an additional tracer experiment in which the tracer is initialized with an idealized north-to-south linearly decreasing temperature field. In this case, EMHT weakens substantially in the Indian sector and becomes stronger in the Atlantic sector, while the total heat entering the ACC is reduced by approximately 40%. See Lines 279-284.

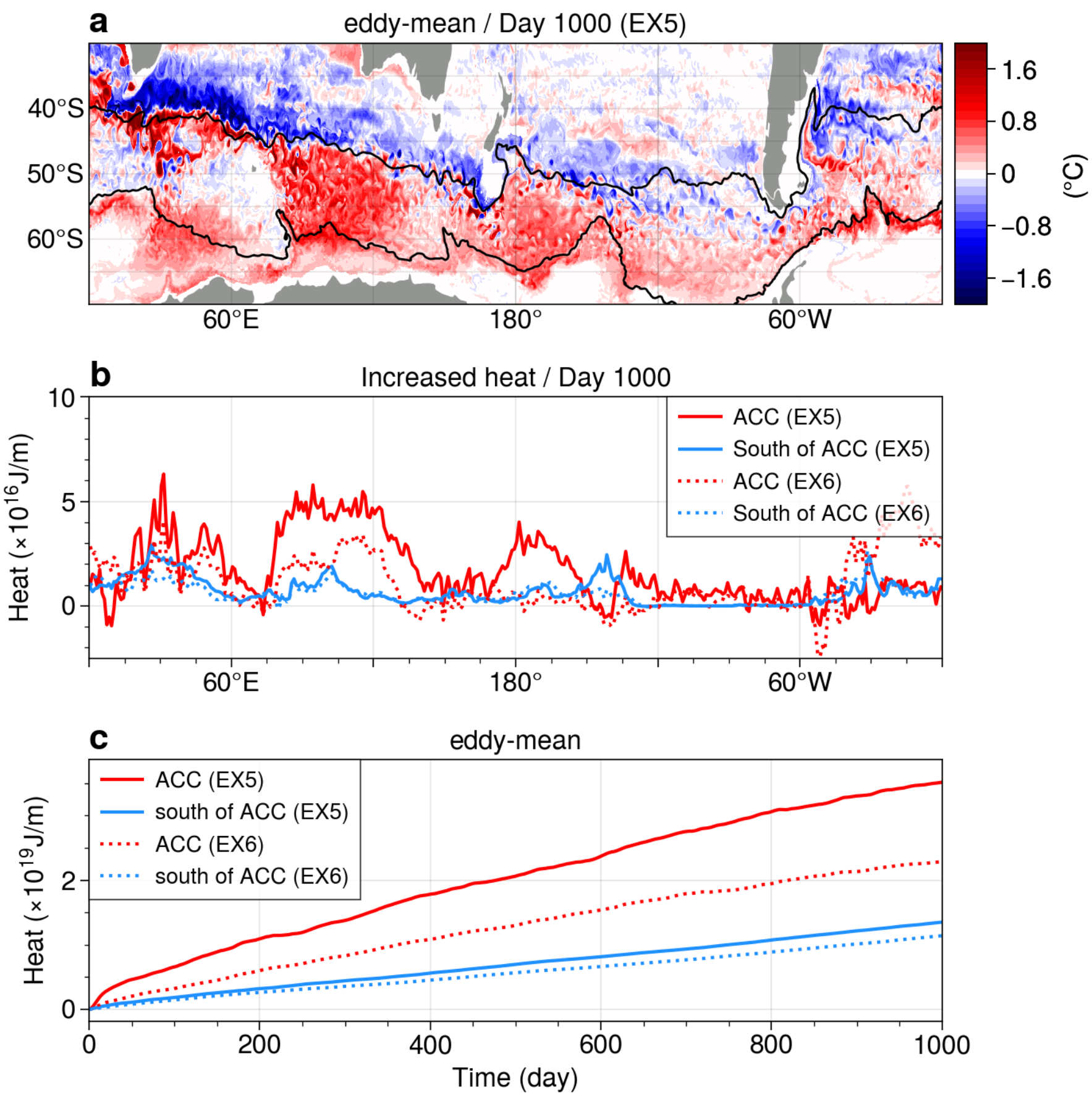

Fig. 5 | Heat transport diagnosed from tracer experiments. a, Spatial distribution of temperature difference (eddy-resolving simulation minus mean-flow simulation) at day 1000. Black contours mark boundaries of the ACC. **b**, Longitudinal distribution of increased heat transport at day 1,000 within the ACC (red) and south of the ACC (blue). **c**, Temporal evolution of cumulative heat transport difference between eddy-resolving and mean-flow simulations for the ACC region (red) and south of the ACC (blue). In **b** and **c**, Solid and dashed lines denote EX5 and EX6, respectively.

R1-19 L157: This information feels like it comes too late. It's interesting that you are able to diagnose these eddy stirring vs. eddy trapping contributions separately. Results aren't surprising but it's an interesting piece to your analysis. Because it is unclear how V' values are calculated, it took me a long time to figure out how you separated between stirring and trapping. At first I thought you were using the velocity product to calculate EMHT but then I couldn't figure out how profiles were separated between stirring and trapping. More details are needed. Might consider adding some details about how many profiles are used in each calculation.

Response: Our original idea was to first estimate the total EMHT from the discrete Argo observations, and then separate the relative contributions of trapping and stirring based on whether the float remained trapped within an eddy over the 10-day interval. This analysis follows the framework of Abernathey and Haller (2018). However, because this part was not central to the main focus of the present study, we have removed the stirring–trapping analysis from the revised manuscript. We agree that this topic is interesting and deserves a more complete treatment. We therefore plan to address it in a separate study that combines observations and numerical simulations.

R1-20 L179: While I believe this statement, I don't know that you've done enough to show us that Argo floats are preferentially crossing at bathymetric regions. At minimum I think you need to highlight where the topographic ridges are. Ideally, maybe you show a subset of trajectories that travel southward at these locations.

Response: Following the reviewer's suggestion, we have added a new supplementary figure showing the Argo trajectories together with the underlying bathymetry. In this figure, the Kerguelen Plateau and Southeast Indian Ridge are explicitly marked. It shows that Argo floats mainly cross into the ACC on the eastern sides of both the Kerguelen Plateau and the Southeast Indian Ridge.

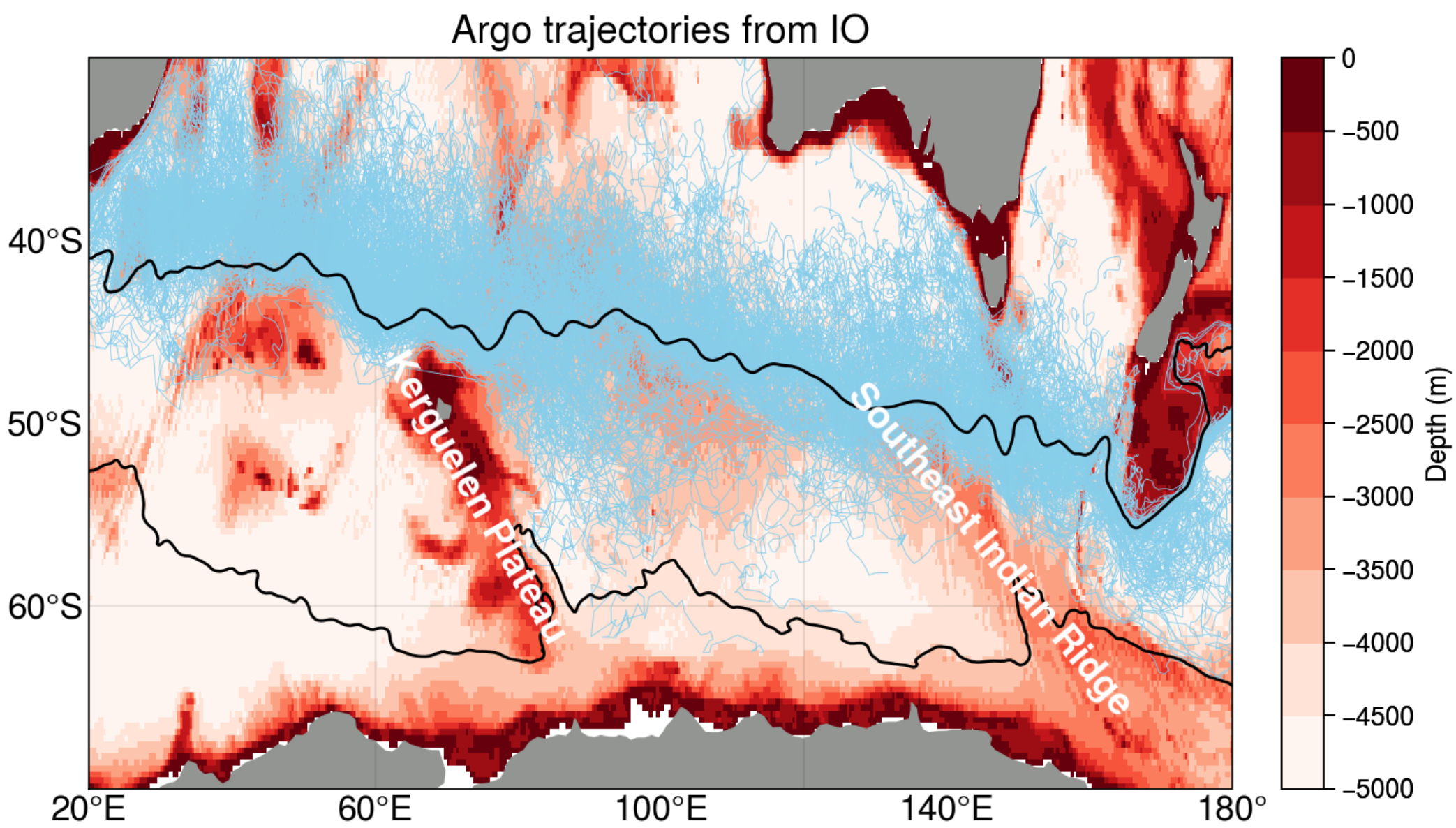

Supplementary Fig. 6 | Argo trajectories from the Indian Ocean sector. Argo trajectories corresponding to Fig. 3b are shown by blue lines. Colour shading indicates bathymetry from ETOPO1. The Kerguelen Plateau and Southeast Indian Ridge are marked. Black lines denote the northern and southern boundaries of the ACC.

R1-21 Fig 3k & 4g: It's interesting that more of the floats/tracer end up moving poleward and crossing the northern boundary of the ACC rather than the southern boundary. I would expect that we would see more poleward movement within the ACC itself. Would be interesting and add to your story to maybe breakdown the movement of floats between those that go southward vs. northward within the ACC

Response: We thank the reviewer for this interesting suggestion. The tracer experiment indeed shows that tracer within the ACC can spread both poleward and equatorward (Fig. 4g). By contrast, the Argo trajectory probability distributions show a clearer signal of poleward occupation across the northern boundary of the ACC. This difference likely reflects two factors. First, EKE is generally stronger near the northern boundary of the ACC, which favors stronger cross-boundary exchange there. Second, the number of Argo trajectories at higher latitudes is relatively limited.

R1-22 L262 – 264: rewrite these sentences. Confusing and seem contradictory.

Response: We have rewritten these sentences in the revised manuscript (Lines 291-294).

"The Southern Ocean limits the direct poleward penetration of warm subtropical water because meridional mean flows are weak. Mesoscale eddies therefore provide a major pathway for poleward heat transport across the ACC. Yet direct observations of EMHT remain largely limited to the surface where eddy activity is strongest, leaving its deep structure poorly understood."

References:

Klocker et al. 2012, Reconciling float based and tracer based estimates of lateral diffusivities

Chapman and Sallée 2017 Isopyncal mixing suppression by the ACC and the Southern Ocean meridional overturning circulation

Phillips and Rintoul 2000 eddy variabitliy and energetics from direct current measurements in the ACC south of Australia

Roach et al. 2016 Horizontal mixing in the Sothern Ocean from Argo float trajectories

Abernathey and Cessi 2014, Topographic enhancement of eddy efficiency in baroclinic equilibration

Watts et al. 2016 estimates of eddy heat flux crossing the ACC from observations in Drake Passage

Ferrari and Nikurashin 2010, Suppression of eddy diffusivity across jets in the Southern Ocean

LaCase et al. 2017; Float derived isopycnal diffusivities in the DIMES experiment

Naveira Garabato et al. 2011, eddy stirring in the Southern Ocean

Balwada et al, 2016, Circulation and stirring in the southeast Pacific Ocean and Scotia Sea sectors of the ACC

We have cited the references suggested by the reviewer and have also conducted an additional literature review. See revised References.

Reviewer #2

This manuscript addresses a long-standing and interesting question in Southern Ocean research by looking at eddy kinetic energy and meridional eddy heat transport using Argo data, in combination with analyses of surface drifters, satellite-based eddy locations, and model output from the Biogeochemical Southern Ocean State Estimate (B-SOSE). While the research questions are interesting, the manuscript has several shortcomings that suggest it is not ready for publication in its present form.

Response: We thank the reviewer for the constructive comments. We have taken your concerns seriously and have performed a comprehensive revision to improve the statistical rigor and the completeness of our literature review. Our point-by-point responses to the specific comments are provided below.

Overarching concerns:

R2-1 The results are based on noisy observations but are presented without error bars or discussion of uncertainty. The authors draw conclusions about differences between ocean basins (Figure 1e), about mechanisms responsible for meridional heat transport (Figure 2b), about distinctions between surface and subsurface velocities (Figure 3c,f,i,j), and about eddy vs mean processes (Figure 4k), all without presenting uncertainty analyses to demonstrate that the curves are different in a statistically significant way.

Response: We thank the reviewer for this important comment. We fully acknowledge that quantifying uncertainty is crucial for ensuring the robustness of our conclusions. In the revised manuscript, we have added standard error bars to figures to explicitly quantify uncertainty. The specific changes are described in our responses to the related comments below.

R2-2 The analysis has been presented without acknowledging an extensive body of previous work dating back several decades. This includes work on the role of eddy transport in the Southern Ocean (e.g. Johnson and Bryden, 1989), on the role of the Indian Ocean in warming the ACC (de Szoeke and Levine, 1981), eddy fluxes inferred from pre-cursors to Argo (Gille, 2003. Float observations of the Southern Ocean: Part 2, Eddy fluxes; subsequent work by a number of groups e.g. Sun et al, 2019, 10.1038/s41598-018-38069-2), and possible biases in such methods (Chinn and Gille, 2007, doi: 10.1175/JTECH2057.1). The lack of context makes it difficult to identify the novelty of the present work.

Response: We thank the reviewer for pointing out these foundational studies. In response to the suggestions from all three reviewers, we have reorganized the Introduction and incorporated the missing references. In addition to the literature suggested, we conducted a comprehensive search to include other relevant studies.

In the revised Introduction, we have clarified the novelty of our work relative to this literature. In particular, Gille (2003) and Chinn and Gille (2007) provided important early attempts to estimate eddy fluxes from discrete float observations. Building on these pioneering studies, we use two decades of accumulated Argo-derived velocity and temperature observations to provide the first observation-based estimates of deep EKE and EMHT at 1000 m in the Southern Ocean. In addition, although previous studies have recognized the major contribution of the Indian Ocean sector to Southern Ocean EMHT, our study provides a quantitative basin-scale assessment of deep EMHT based on observations and numerical experiments. See Lines 37-78 and Lines 435-440.

Specific comments:

R2-3 In the introduction the discussion of eddy meridional heat flux has significant gaps. Previous authors have identified the role of eddies and the vertical structure of eddies. The authors should be clear about the distinctions between prior work and their new findings. I believe they are suggesting that their work puts forward an idea that regional temperature variations matter. This sounds useful, but in the end, I'm not sure that the authors have demonstrated how their work differs from heat budgets assembled by De Szoeke and Levine or from ocean inverse models.

Response: We thank the reviewer for this important comment. Following the reviewer's suggestion, we have reorganized the Introduction to better acknowledge previous contributions and to clarify the main novelty of our work.

The first main contribution of our study is that we use two decades of accumulated Argo-derived velocity and temperature observations to provide the first observation-based estimates of deep EKE and EMHT at 1000 m in the Southern Ocean. The second contribution is that we quantify the basin-specific contributions to deep EMHT from the Indian, Pacific and Atlantic sectors using both observations and numerical experiments.

We also clarify the relationship between our work and previous studies of eddy vertical structure. For example, Frenger et al. (2015) previously examined the vertical structure of Southern Ocean eddies. Our study does not present eddy vertical structure itself as the central novelty. Instead, we use a more detailed regional

partitioning to show that regional differences in eddy thermal structure contribute to different basin contributions to EMHT at 1000 m. In particular, the Indian Ocean sector contributes about 40% of surface EMHT but about 60% of deep EMHT.

We agree that de Szoeke and Levine (1981) provided a comprehensive forward-looking heat-budget analysis. Our study provides an updated observational perspective by using the modern Argo observing system. Their analysis focused on the Polar Front and was based on relatively limited hydrographic stations, and they also noted the uneven distribution of observations and the lack of wintertime data. By contrast, our analysis uses two decades of Argo-derived velocity and temperature observations to directly quantify deep EMHT and its basin-scale structure. The revised Introduction has been updated accordingly (Lines 37-78).

R2-4 Figure 1e. As noted above, the plot needs an uncertainty analysis. How robust are results? The implications are interesting but need to be demonstrated to be robust. Could the sector-by-sector findings be attributable to latitude ranges or distribution of data, or to the presence of the Kerguelen Plateau?

Response: We have reorganized Fig. 1 and moved the plot showing the eddy vertical structure to the Supplementary Information (now Supplementary Fig. 4). In this revised figure, we have also included the SOSE model results. The comparison shows that the deep-ocean eddy anomalies simulated by the model are significantly weaker than the observations, which explains why the model fails to reproduce the strong deep EMHT.

Regarding the composite of Southern Ocean eddies, our methodology follows Frenger et al. (2015), utilizing a larger, updated volume of available Argo data. Whereas Frenger et al. (2015) divided the domain mainly by latitude, separating the region north of the ACC from the ACC, we divide the Southern Ocean into four sectors to examine basin-scale differences. The number of Argo profiles in each sector exceeds 600,000, substantially larger than in Frenger et al. (2015). This extensive data coverage supports the robustness of the observed basin-scale differences. Because our focus is on basin-scale contrasts, localized topographic features such as the Kerguelen Plateau are unlikely to dominate the composite results for the entire Indian sector.

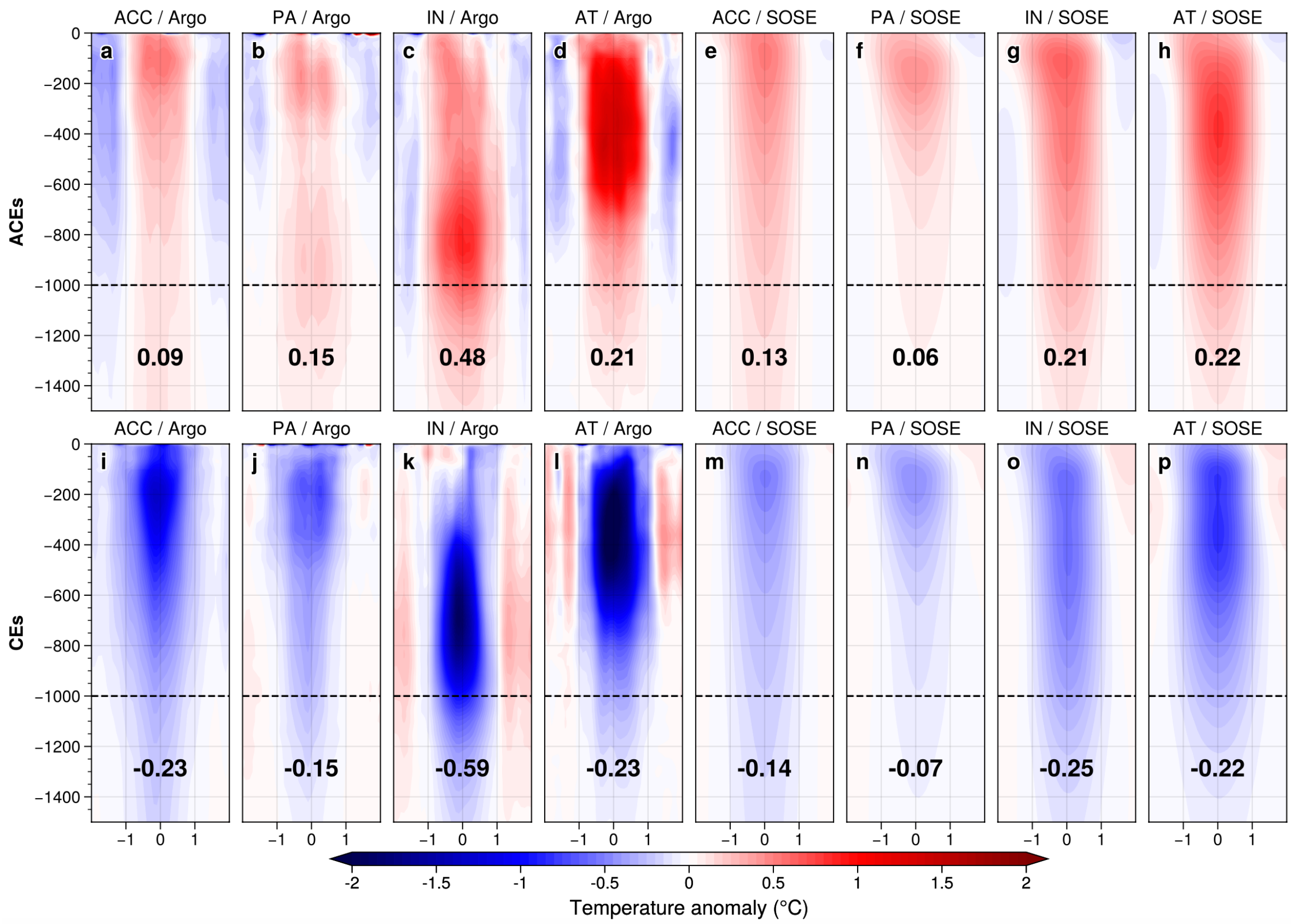

Supplementary Fig. 4 | Composite vertical structure of mesoscale eddy temperature anomalies from Argo and SOSE. a–d, Composite temperature anomalies for anticyclonic eddies derived from Argo in the ACC (**a**), Pacific (**b**), Indian (**c**), and Atlantic (**d**) sectors. **e–h,** Same as **a–d**, but derived from SOSE. **i–l,** Composite temperature anomalies for cyclonic eddies derived from Argo in the ACC (**i**), Pacific (**j**), Indian (**k**), and Atlantic (**l**) sectors. **m–p,** Same as **i–l**, but derived from SOSE. Numbers in each panel indicate the mean temperature anomaly within one normalized eddy radius of the eddy center. Temperature anomalies are calculated relative to the monthly climatological mean field.

R2-5 Figure 2b. As noted above, the figure cannot be interpreted without uncertainty estimates.

Response: In the revised manuscript, we have reorganized Fig. 2 by removing the trapping–stirring analysis and adding the meridional temperature gradient (dT/dy) and the ratio of deep to surface EMHT, which together help clarify the physical interpretation of the results.

To address the uncertainty in Fig. 2b, we now provide explicit uncertainty estimates. Following the suggestion of Reviewer 1, we first use SOSE-driven particle experiments to evaluate how well our particle-

based method can reproduce the “true” EKE and EMHT diagnosed directly from the model. The results show that the method captures the spatial patterns well, although the magnitudes are slightly underestimated. Based on 50 random resampling experiments, we quantify the uncertainty associated with this method.

For the observational estimate, the number of available Argo samples is insufficient for independent subsampling in the same way. We therefore use bootstrap resampling with replacement to estimate the uncertainty of the zonally integrated EMHT shown in Fig. 2b. This uncertainty is now displayed in the revised figure. The corresponding revisions are provided in Lines 457-470.

R2-6 lines 149-151. The partitioning of the heat transport estimates does not appear justified. We usually think of the Antarctic Circumpolar Current as having an equivalent barotropic structure, with variability tapering with depth. It might make sense to incorporate this structure into an extraplation with depth. It does not seem credible to partition the ocean into a shallow surface layer and a uniform deep layer.

Response: We thank the reviewer for pointing this out. The original discussion was not sufficiently precise in describing the vertical layers. In the revised manuscript, we no longer broadly discuss the 500–1500 m range as a single “deep” layer, and instead focus more explicitly on the comparison between the surface and 1000 m. See Lines 166-169.

R2-7 line 153. "surface poleward heat transport is suppressed": There is no reason to expect poleward heat transport at the surface in the Southern Ocean. Decades of analyses of meridional overturning circulation in the Southern Ocean have emphasized that surface transport is expected to take water northward, and the mid-depth flow should be poleward.

Response: We fully agree that the large-scale mean surface heat transport in the Southern Ocean is directed northward. Our original phrasing here was unclear. In that context, we were referring specifically to the EMHT, rather than the total or mean transport. Because the sea surface temperature decreases poleward, eddies naturally act to transport heat poleward down the temperature gradient. We have revised the text in Line 166-170.

R2-8 line 154. "large subsurface temperature anomalies": What is the evidence for subsurface temperature anomalies. This is something that requires figures and quantitative evidence.

Response: The quantitative evidence for these subsurface temperature anomalies is provided in our eddy composite results. As shown in Supplementary Fig. 4, the deep-layer temperature anomalies within eddies are substantial. Specifically, in the Indian Ocean sector, the observed temperature anomalies within the eddies exceed 1.5°C. Furthermore, we have conducted a new idealized tracer experiment. The results demonstrate that if the deep-ocean temperature profile is modified to decrease linearly with latitude (mirroring the surface layer), the heat transport entering the ACC is significantly reduced. In the revised manuscript, we have updated the text in Lines 118-120 and Lines 279-284.

R2-9 lines 159-160. "Profiles persistently located within an eddy's interior for consecutive 10 days are attributed to eddy trapping": Since Argo floats profile every 10 days, and the Jason/Sentinel 6 series of altimeters measures every 10 days, a day-day threshold seems curiously short for this analysis. Maybe the text is not clearly worded. However, I think a longer time window would be appropriate, and if a 10-day window is the optimal choice, then the justification needs to be more clearly explained.

Response: We realize that the discussion on eddy trapping and stirring is tangential to the core focus of this study. To streamline the paper, we have removed this content from the revised manuscript.

R2-10 line 165. "likely stems from intense eddy-eddy and eddy-flow interactions": What evidence demonstrates that this is the likely explanation? Is this based on reference [31] or based on calculations carried out for this study? This needs to be explained in the manuscript.

Response: The discussion regarding eddy interactions, trapping, and stirring was originally included to build upon our previous work. However, since all three reviewers noted that this section felt disconnected from the main storyline, we have removed it entirely from the revised manuscript. We have streamlined the text to focus strictly on the deep EMHT.

R2-11 lines 173-174. "We analyze the trajectories of Argo floats from four regions over a 1000-day period starting from January 1, 2013, ..." Argo floats are not designed to provide long trajectories, and the transition from ARGOS to Iridium led to substantial changes in the behavior of multi-cycle float trajectories. The text

implies that consecutive displacements have been concatenated. Is that the case? Have they been screened to remove floats with long surface parking depths? These choices require more explanation.

Response: In our analysis, each drifter or Argo float is counted only once, and consecutive Argo displacements are not concatenated to construct artificial long trajectories. Once a drifter or float first enters the prescribed source region, we follow its subsequent trajectory for up to 1,000 days, if such records are available. In fact, 67.5% of the Argo floats used in our analysis remain available for more than 1,000 days, whereas surface drifters generally have shorter lifetimes, with only about 14.6% lasting longer than 1,000 days (Supplementary Fig. 7). This is consistent with the typical Argo mission design described by Riser et al. (2016): "A typical float will repeat this 10-day cycle in excess of 200 times over the course of five years or more."

Regarding the potential influence of long surface residence times, the Argo velocity product used in this study applies an extrapolation procedure to reduce the impact of surface drift and estimate the parking-depth velocity. For the trajectory analysis, we do not further exclude samples because of the limited number of available trajectories. To assess the robustness of the observed features, we conduct an independent particle experiment driven by SOSE velocity fields at the surface and 1000 m (also see our response to R1-3). The SOSE-driven particles show a similar pattern to the observations but with a stronger surface–deep separation (Fig. 3), because the synthetic particles provide complete trajectories over the prescribed 1,000-day period. See Lines 202-217.

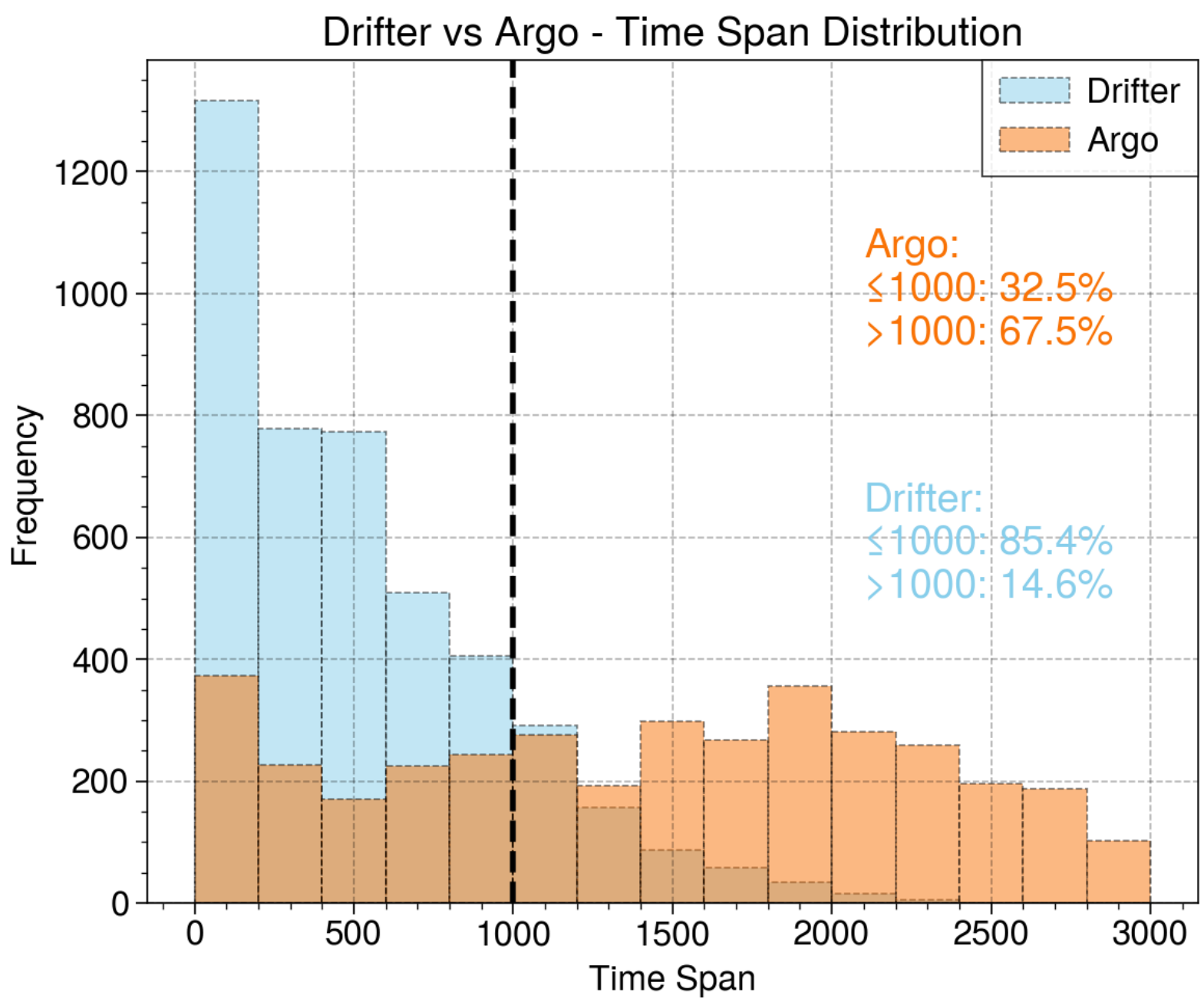

Supplementary Fig. 7 | Distribution of trajectory durations for surface drifters and Argo floats. Histogram of trajectory durations used in the trajectory analysis. Blue bars denote surface drifters, and orange bars denote Argo floats.

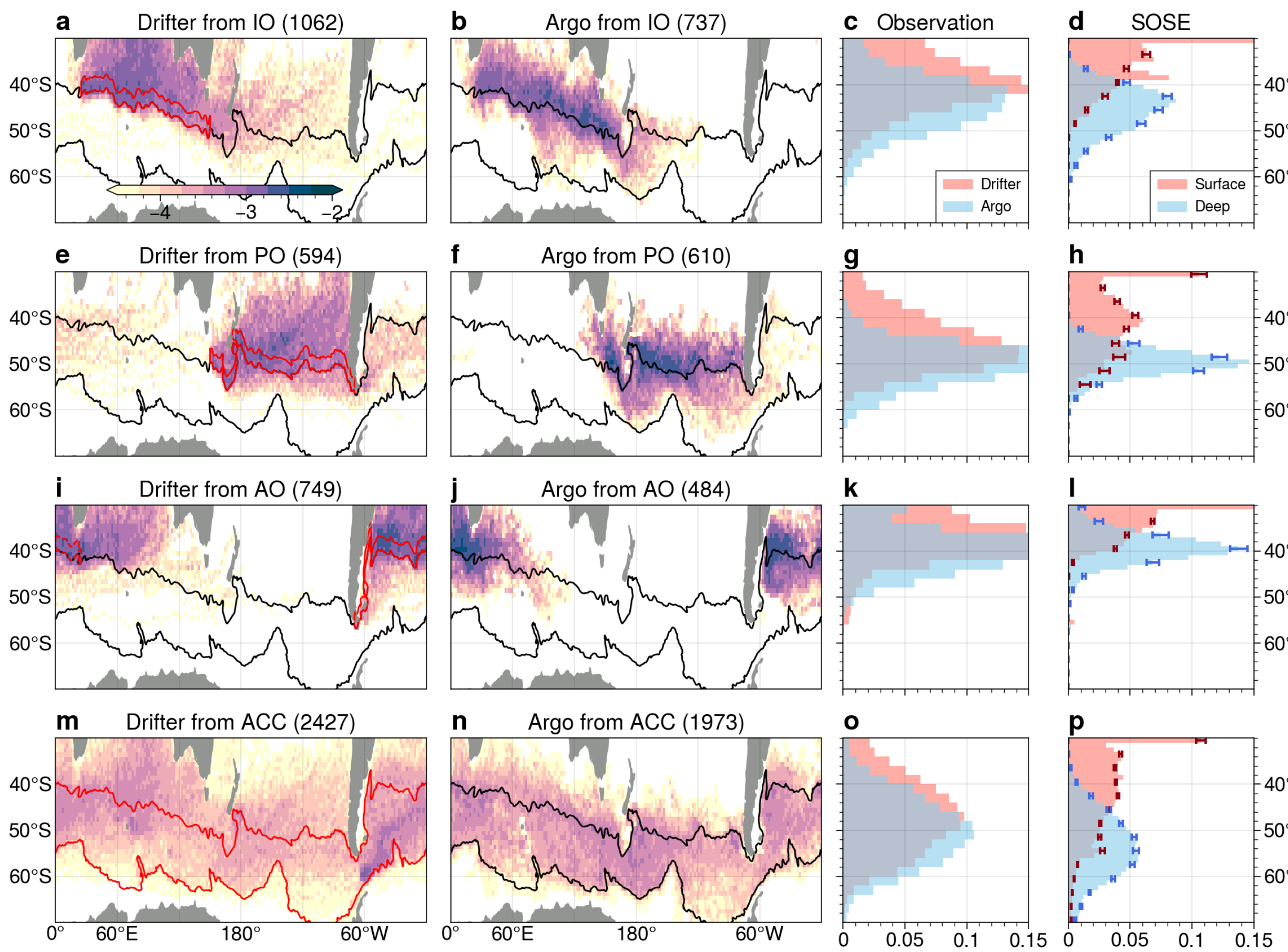

Fig. 3 | Spatial and latitudinal probability distributions of surface drifters, Argo floats, and SOSE particles in the Southern Ocean. a–d, Spatial probability distributions of surface drifters (**a**) and Argo floats (**b**) from the Indian Ocean sector over 1000 days, along with the latitudinal probability distributions of the observations (**c**) and SOSE-driven particles (**d**). **e–h**, from the Pacific Ocean sector. **i–l**, from the Atlantic Ocean sector. **m–p**, from the entire ACC region. Numbers in parentheses indicate total counts for each region. Red lines in the first column outline the initial positions of all observations and model particles. Error bars in the fourth column represent the standard deviation of 50 random subsamples. Black lines mark the northern and southern boundaries of the ACC.

R2-12 lines 180-181. "Statistical results indicate that 17% of Argo floats cross the ACC northern boundary toward the pole, compared to only 7% of surface drifters, ..." I don't understand this sentence. Is this saying

that floats move southward below the surface? That doesn't seem like a novel finding, given the bulk of previous literature. The quantitative results need to be reported with statistical uncertainties based on the standard error of the mean.

R2-13 lines 181-182. "... Argo float trajectories demonstrating greater penetration of deep water than surface water into higher latitudes." These findings are not demonstrated by Figures 2 and 3. More evidence is needed if the authors wish to make this point.

R2-14 line 184, reference to Fig. 3l. Comments about meridional movement are only meaningful if the authors can also show that the pdfs are statistically different. The distributions in Fig. 3l could result from differences in deployment locations or sparse sampling leading to non-robust statistics.

Response: As comments R2-12, R2-13 and R2-14 all concern the interpretation and robustness of the trajectory analysis in Fig. 3, we address them together here.

Following the reviewer's comments, and also in response to R1-3, we have substantially revised Fig. 3. The original trajectory plots have been replaced by trajectory probability distributions. We also restrict the initial positions of both surface drifters and Argo floats to a narrow band within 3° north of the ACC northern boundary, in order to reduce the influence of different deployment locations. The revised trajectory probability distributions in Fig. 3a and 3b show that 29% of Argo trajectory positions are located poleward of the ACC northern boundary, compared with 8% for surface drifters. The latitudinal histograms in the third column further reveal opposite meridional preferences between Argo floats and surface drifters.

To assess the robustness of this contrast, we use SOSE-driven particle experiments. We repeat the calculation with 50 random subsamples, each matching the number of observed trajectories. The resulting standard deviations are small, generally less than 8% of the corresponding mean values, indicating that the difference between the surface and deep trajectory probability distributions is robust and unlikely to result solely from sparse sampling. Therefore, the observations and SOSE-driven particle experiments support a statistically robust difference between surface and deep meridional spreading.

We agree that our previous wording overstated the direct link between Argo trajectories and eddy-driven transport. Trajectories are influenced by the full flow, including both mean flow and eddies. To further isolate the role of mesoscale eddies, we introduce tracer experiments. The tracer distributions show strong spatial consistency with the Argo trajectory probability distributions (Fig. 3b and Fig. 4a), and the comparison between

full-flow and mean-flow tracer experiments allows us to isolate the eddy-induced contribution. These experiments show that mesoscale eddies are responsible for the enhanced poleward redistribution and associated EMHT.

In addition, to provide a more direct visualization of the influence of deep EMHT on high-latitude regions, we have added a three-dimensional tracer experiment in the Supplementary Information (Supplementary Fig. 9). This experiment shows that 1000-m-origin tracer (used here as a proxy for heat) crosses the ACC more effectively and spreads farther into high-latitude regions, whereas surface-origin tracer remains largely confined near the northern boundary of the ACC. These revisions are included in Fig. 3, Supplementary Fig. 9, and Lines 193-206.

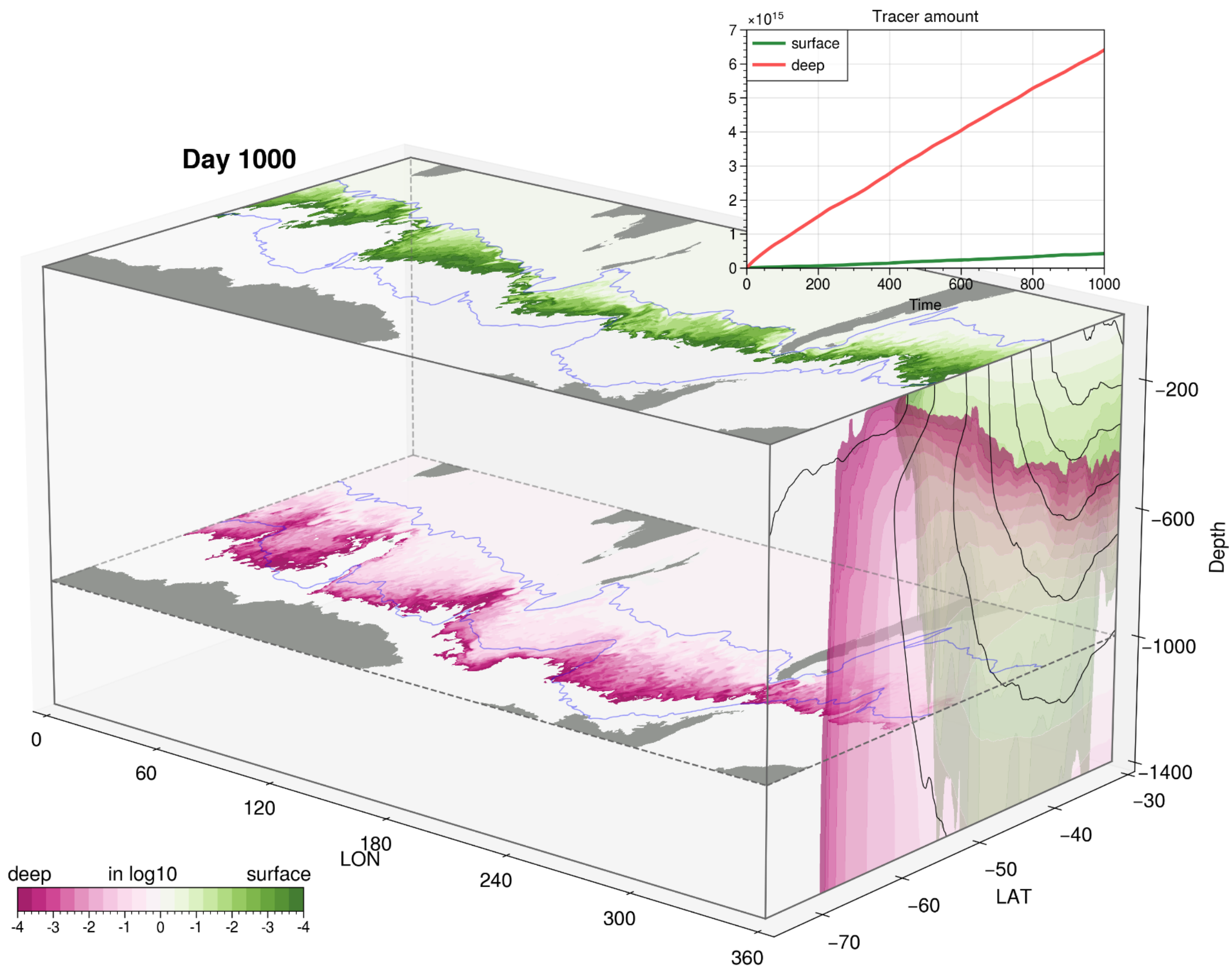

Supplementary Fig. 9 | Three-dimensional tracer dispersion in the offline experiment. The two horizontal planes show tracer concentration at the surface and 1000 m at day 1,000. The tracer is initialized to 1 north of the ACC and continuously restored to 1 in this source region. The offline experiment is driven by the three-dimensional SOSE velocity field. Green and red denote tracers released from the surface and 1000 m,

respectively. Blue solid lines mark the northern and southern boundaries of the ACC. The right-hand sections show zonal-mean tracer concentration and temperature profiles at day 1,000. The inset time series shows the cumulative tracer amount crossing the ACC, with red and green lines denoting contributions from the 1000 m and surface tracers, respectively.

Link: https://vimeo.com/1188593847?fl=pl&fe=sh

R2-15 Figure 4. As noted above, at a minimum results in Figure 4k should include uncertainties, and ideally the maps should use cross-hatching to indicate non-statistically significant concentrations (or differences), and Figures 4i and 4j should show uncertainty in the ranges of distributions.

Response: We thank the reviewer for this suggestion. We would like to clarify that Fig. 4 is based on an offline tracer experiment driven by the SOSE velocity field. For a given velocity field and initial tracer concentration, the tracer distribution is deterministic. Therefore, statistical uncertainty or statistical significance is not directly defined for this figure.

The main uncertainty we can identify is the threshold used to define the minimum tracer concentration included in the calculation. We have tested thresholds starting from 10^{-1} to 10^{-10}, and the integrated tracer amount shows little sensitivity once the threshold is smaller than 10^{-4} (Fig. C1). We therefore use 10^{-4} as the threshold and have added an explanation of this choice in the revised manuscript (Lines 487-489).

If we have misunderstood the reviewer's meaning, we would be happy to revise the figure further.

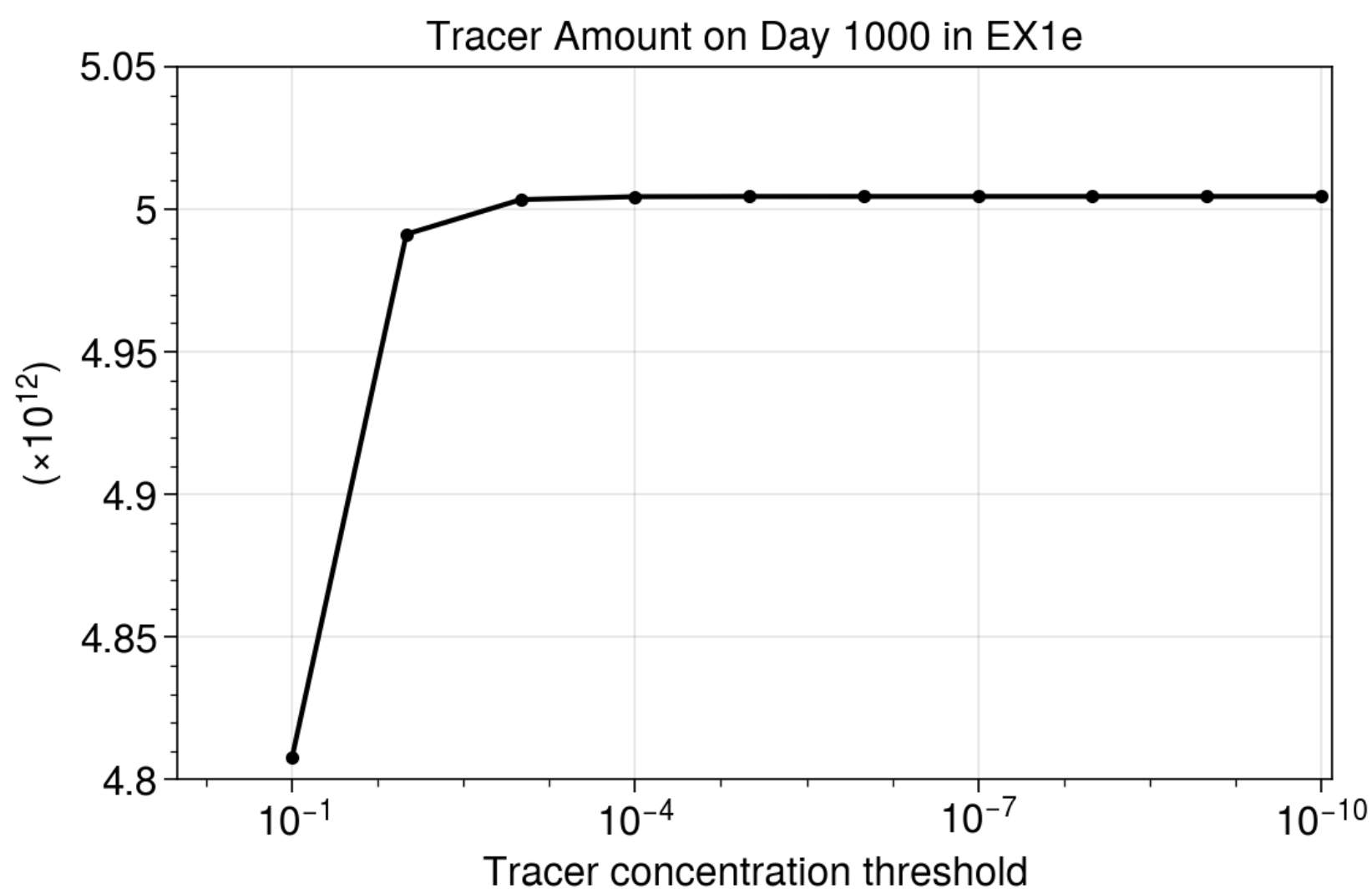

Fig. C1 | Total tracer inventory as a function of concentration threshold.

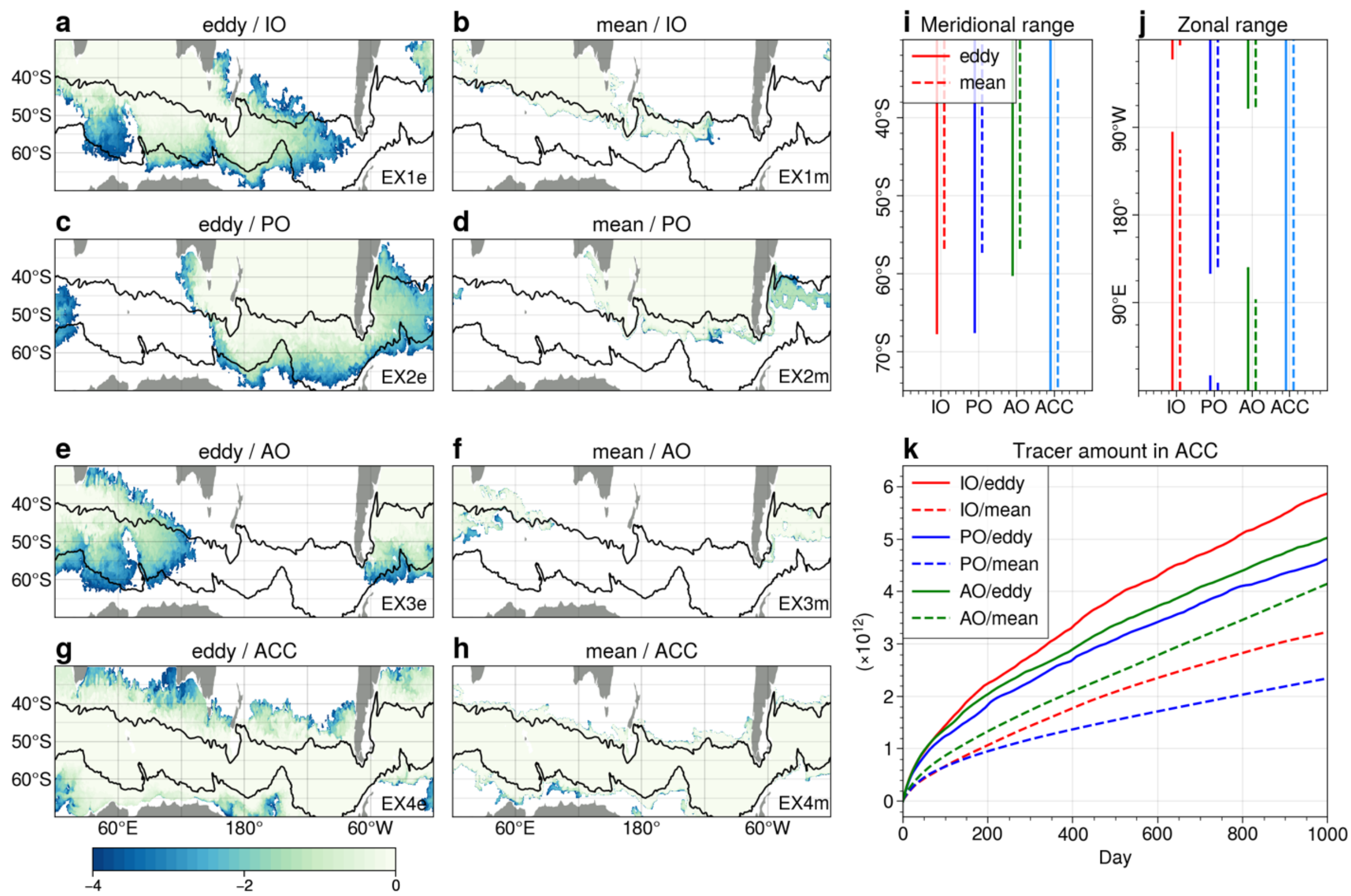

Fig. 4 | Offline tracer experiments in the deep Southern Ocean. a-h, Tracer concentration at 1000 m depth released in four regions and advected by: full flow (eddy-resolving) in the Indian Ocean (**a**), Pacific Ocean (**c**), Atlantic Ocean (**e**), and ACC region (**g**); and by mean flow alone in corresponding regions (**b,d,f,h**). **i**, Meridional range of tracer distribution across different basins. **j**, Zonal extent of tracer distribution. **k**, Temporal series of cumulative tracer amount entering the ACC from the three ocean sectors (Indian, Pacific, Atlantic), with solid and dashed lines representing full flow and mean flow experiments, respectively. Black contours mark the ACC boundaries, with colors indicating tracer concentration on a logarithmic scale. Note that the initial tracer concentration in these four experiments is 1, which is used to mark the water in different basins.

R2-16 lines 227-230. "These results establish mesoscale eddies as critical conduits for deep subtropical-origin water intrusion into polar regions, with transport magnitude and efficiency reaching twice that of mean flow, underscoring their role in meridional heat and mass exchange in the deep Southern Ocean." This is not a profound result, given that the key role of eddies in governing Southern Ocean overturning circulation and heat transport has permeated the published literature since the 1980s. (See Johnson and Bryden, 1989, for example.)

Response: We agree with the reviewer that the key role of mesoscale eddies in Southern Ocean overturning and heat transport has long been recognized, including by Johnson and Bryden (1989) and subsequent studies. Our intention was not to present the general importance of eddies as a new finding. We have revised the text to avoid this overstatement and to place the result more clearly in the context of previous work in Lines 252-254.

In this section, our goal is to use the tracer experiments to quantify the relative effects of mesoscale eddies and the mean flow under the same integration time and initial tracer conditions. This comparison shows how much farther subtropical-origin water can spread poleward when eddies are included, and how the transport magnitude and efficiency differ from the mean flow. We believe this quantitative comparison is useful for understanding the role of eddies in deep water-mass redistribution and heat exchange in the Southern Ocean.

R2-17 lines 247-284. "The preceding Argo trajectory analysis indicates these warm intrusions originate primarily from the Indian Ocean." As noted above, the notion that the ACC warms in the Indian sector has permeated the published literature for many decades. This is not a novel resut and needs to be presented in the context of prior work (e.g. De Szoeke and Levine, 1981).

Response: We thank the reviewer for this clarification. We have carefully re-read de Szoeke and Levine (1981). While this study provides important early context for eddy heat transport in the Southern Ocean, it does not specifically discuss warming of the ACC in the Indian sector. We have therefore reviewed additional relevant studies that more directly address the role of the Indian sector in Southern Ocean heat transport, including Gille (2003), Volkov et al. (2010), and Foppert et al. (2017). We have revised the text accordingly to better place our results in this context.

Although previous studies have noted the importance of the Indian Ocean sector, they did not emphasize the contrast between surface and deep EMHT. This contrast is a key point of our study. In the revised Fig. 2e, the Indian Ocean sector accounts for only about 40% of surface EMHT, because the Pacific and Atlantic sectors also contribute substantially. At 1000 m, however, eddy-induced temperature anomalies in the Pacific and Atlantic sectors are much weaker, so the Indian Ocean sector contributes about 60% of deep EMHT despite occupying only about one-third of the circumpolar longitude range. Both the Argo-based observations and tracer experiments support this basin-dependent contrast in deep EMHT. The corresponding revisions are provided in Lines 172-174 and Lines 272-274.

R2-18 lines 461-462. "To evaluate the impact of high-frequency motions," The B-SOSE results reported here appear to differ from data based results by Chinn and Gille, 2007.

Response: Chinn and Gille (2007) provided a useful benchmark for assessing sampling effects in float-based eddy flux estimates. In their study, daily float positions allowed the 1-day estimate to be used as a reference for testing the sensitivity of eddy fluxes to longer sampling intervals. Our Argo dataset differs from theirs because Argo-derived velocities are available only as approximately 10-day displacement velocities, so the same direct sensitivity test cannot be applied.

To assess the effect of high-frequency motions, we instead use SOSE velocity fields. A spectral analysis shows that motions with periods shorter than 10 days account for only about 1% of the total kinetic energy at 1000 m. This suggests that 10-day displacement velocities retain most of the velocity variability at this depth and are suitable for estimating climatological deep EMHT.

Chinn and Gille (2007) also emphasized the importance of sample number within each grid cell. They showed that eddy heat flux can be largely captured when the number of samples in a 2°×2° grid cell exceeds about 100. In our analysis, we use 2°×1° grid cells. Except poleward of 60°S, most grid cells contain more than 50 samples (Fig. C2), which is broadly consistent with their sampling criterion after accounting for our smaller meridional grid size. We have added this discussion in the revised Methods to clarify the relationship between our approach and Chinn and Gille (2007). See Lines 435-440.

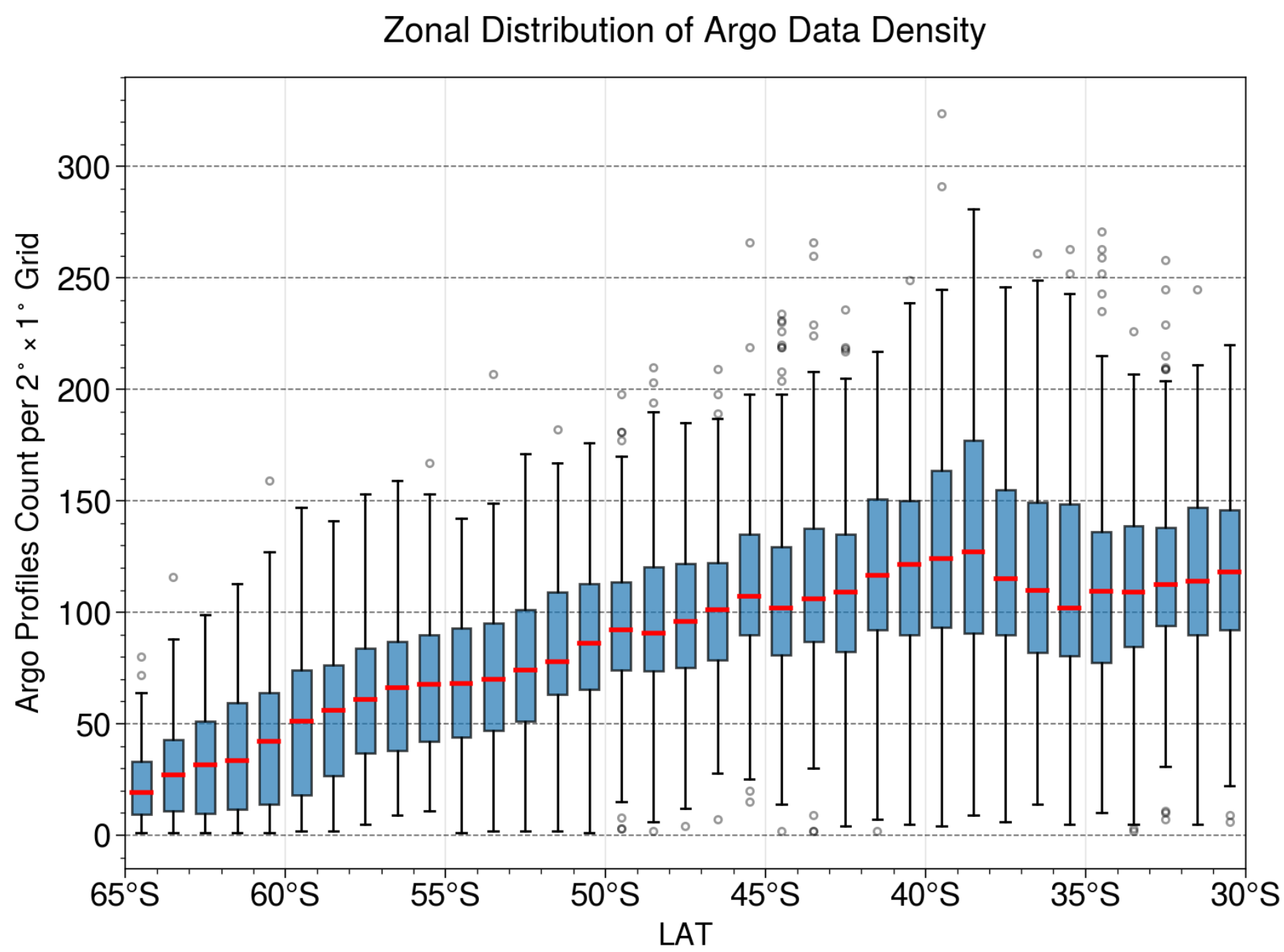

Fig. C2 | Latitudinal distribution of Argo sample density. Boxplots show the zonal distribution of the number of Argo samples per grid cell at each latitude. The red line denotes the median, boxes indicate the interquartile range, whiskers show the full range excluding outliers, and points denote outliers.

R2-19 line 479 and following. The calculation of EMHT using contributions from coherent vortices needs further explanation. Eddy fluxes are normally computed assuming eddies to encompass all anomalies---not just coherent vortices. This separation needs to be justified more thoroughly.

Response: Because the discussion on coherent eddies is disconnected from the main storyline, we have removed this section entirely from the revised manuscript.

R2-20 lines 482-483. "it it remains within the same eddy for 10 days". Assuming that the authors do intend to distinguish eddy impacts due to coherent vortices and eddy variability due to everything else, the uncertainties of this separation need to be quantified. As noted above, 10 days is fairly short, particularly given the satellite orbits and the Argo surfacing times. Do some floats appear to enter and leave and then reenter the same eddies? How uncertain is this calculation?

Response: This analysis follows the framework of Abernathey and Haller (2018). However, because this part was not central to the main focus of the present study, we have removed the stirring–trapping analysis from the revised manuscript. We think that this topic is interesting and deserves a more complete treatment. We therefore plan to address it in a separate study that combines observations and numerical simulations.

R2-21 Points of grammar/style:

a. line 67: "temperature" -> "temperatures that are"

b. line 72. "this process" -> "these processes"

c. line 74. "further": The text is unclear about what the authors investigate further than previous work investigated. I would suggest omitting the word.

Response: We thank the reviewer for pointing this out. We have rewritten the Introduction, and these errors are no longer present in the revised manuscript.

Reviewer #3:

I read the manuscript "enhanced poleward heat transport by mesoscale eddies in the deep Southern Ocean" by Liu et al. with great interest. In this manuscript the authors use observations to show nicely that the eddy heat transport across the Southern Ocean is much larger relative to surface eddy heat transports than was previously assumed. These results are quite crucial to understand future changes in the Southern Ocean, in particular since models used for climate projections usually parameterise these processes, with parameterisations generally not able to represent regionally varying eddy transport of heat with unknown downstream impacts on the rest of the climate system.

The main issues I have here are regarding some of the text which has some room for improvement. In general, I think that these are small fixes that are quick to do, and I hope to see this manuscript published soon.

Response: We thank the reviewer for the positive and encouraging comments. We have revised the manuscript accordingly to improve the clarity and precision of the text.

R3-1 title: I'm not sure I like the word "enhanced" in the title since it is unclear by reading just the title what the heat transport is enhanced relative to.

Response: We agree with the reviewer that the word "enhanced" is ambiguous in a standalone title without a clear baseline for comparison. To eliminate this ambiguity and explicitly highlight the robust, observation-based nature of our findings, we have revised the title to: "Observed substantial poleward heat transport by mesoscale eddies in the deep Southern Ocean".

R3-2 line 38: "The prevailing westerly winds over this region drive the eastward-flowing ACC….this is quite outdated thinking! One of the co-authors should surely know better (https://agupubs.onlinelibrary.wiley.com/doi/full/10.1029/2010GL044777). You get a strong ACC even in the limit of zero wind (e.g. https://journals.ametsoc.org/view/journals/phoc/43/3/jpo-d-12-095.1.xml, https://tellusjournal.org/articles/3231/files/6582dc3c97162.pdf, https://tellusjournal.org/articles/10.16993/tellusa.4107).

Response: We thank the reviewer for pointing this out. We have revised the Introduction accordingly and now cite the recommended studies, including Hogg (2010), Munday et al. (2013) and Klocker et al. (2025). See Lines 509, 514, and 516.

R3-3 line 61: “The magnitude of the EMHT is determined by both eddy activity and the background temperature field”. This, and subsequent text latter in the manuscript, makes little mention of the effects of the mean flow, and then the authors then use words like “counterintuitive” or “surprising” to describe that eddy heat transport is so large at depth. The suppression of eddy mixing by mean flow at depth is now quite well-known (e.g. https://journals.ametsoc.org/view/journals/phoc/40/7/2010jpo4278.1.xml, https://journals.ametsoc.org/view/journals/phoc/42/9/jpo-d-11-0205.1.xml, https://elischolar.library.yale.edu/journal_of_marine_research/352/). So rather than explaining these results as so surprising, the authors should explain that according to theory/idealised experiments eddy mixing is dependent on EKE and mixing length, with the latter being modified by the mean flow. As a next step, the authors can then move on to the main point of the manuscript, showing that their observations are consistent with this theory, leading to enhance heat transport at depth.

Response: We thank the reviewer for this helpful suggestion. We have revised the Introduction and related text to explicitly acknowledge the suppression of eddy mixing by the mean flow, following Ferrari and Nikurashin (2010) and Klocker et al. (2012a, b). We have also removed terms such as “counterintuitive” and “surprising”. The revised manuscript now emphasizes that our main contribution is to provide direct observational evidence for deep EMHT in the Southern Ocean based on two decades of discrete Argo observations. We further emphasize that the observed vertical structure of EMHT provides observational support for the theoretical framework in which mean flows regulate eddy mixing. See Lines 162-174, 551, 555, and 559.

R3-4 line 127-129: Are the acronyms “AC” and “EAC” really necessary for this little use in the manuscript?

Response: We have removed the acronyms "AC" and "EAC" and spelled out their full names throughout the revised manuscript.

R3-5 line 148-156: see comment on line 61; relate to the existing literature rather than pretend this is so counterintuitive. These results are actually quite logic, and that doesn't make them less exciting.

Response: Following our response to R3-3, we have revised this section to relate the results to the existing theoretical literature on mean-flow suppression of eddy mixing. The term "counterintuitive" has been removed.

R3-6 line 157-166: Is this paragraph really needed in the manuscript? I'm not convinced it adds anything.

Response: We agree with the reviewer. The discussion on eddy trapping and eddy stirring was tangential to the core message of the paper. We have removed this paragraph from the revised manuscript, which helps streamline the text and keeps the focus tightly on our main findings.

R3-7 line 200-202: I'm not sure why the mean flow experiments are necessary. Of course there is almost no meridional flow here. And if wanting to "..evaluate the role of eddies…" why not show eddy-only simulations (such as https://elischolar.library.yale.edu/journal_of_marine_research/352/) or at least compare to previous results.

Response: Although the meridional component of the mean flow is weak, the mean flow still strongly shapes the zonal distribution of the tracer and therefore provides an important background state for tracer dispersion (Abernathey et al. 2013; Liu et al. 2024). In our offline simulations, the tracer distribution driven by the full flow closely resembles the observed Argo trajectory patterns (Fig. 3b and Fig. 4a), whereas an eddy-only experiment would not be expected to reproduce this structure.

Our experiment differs in purpose from eddy-only experiments such as Klocker et al. (2012b). Their study uses eddy-only and full-flow comparisons to demonstrate the suppression of eddy mixing by the mean flow. In contrast, our goal is to isolate the heat redistribution associated with mesoscale eddies by comparing the full-flow simulation with a mean-flow simulation. This comparison allows us to quantify the magnitude and efficiency of eddy-induced heat transport relative to the mean-flow baseline.

We also add a new sensitivity experiment, EX6, in which the tracer is initialized with an idealized north-to-south linearly decreasing temperature field. This experiment demonstrates the importance of the background tracer gradient in setting both the magnitude and spatial distribution of EMHT (Lines 279-284). In addition, we conduct a three-dimensional tracer simulation to illustrate the potential influence of deep EMHT on high-

latitude regions (Line 310). This simulation is included in the Supplementary Information, together with a 3D movie provided through a link.

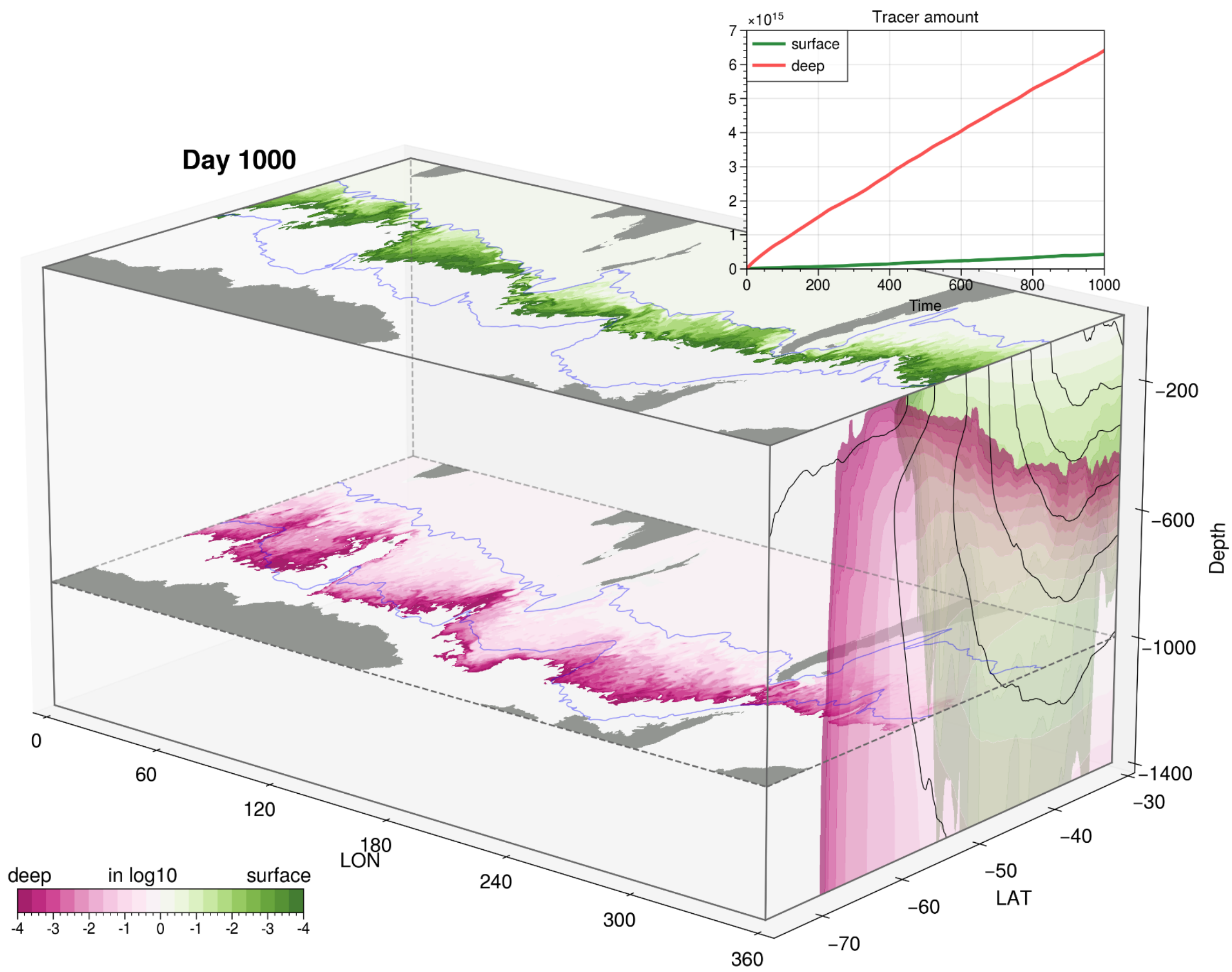

Supplementary Fig. 9 | Three-dimensional tracer dispersion in the offline experiment. The two horizontal planes show tracer concentration at the surface and 1000 m at day 1,000. The tracer is initialized to 1 north of the ACC and continuously restored to 1 in this source region. The offline experiment is driven by the three-dimensional SOSE velocity field. Green and red denote tracers released from the surface and 1000 m, respectively. Blue solid lines mark the northern and southern boundaries of the ACC. The right-hand sections show zonal-mean tracer concentration and temperature profiles at day 1,000. The inset time series shows the cumulative tracer amount crossing the ACC, with red and green lines denoting contributions from the 1000 m and surface tracers, respectively.

Link: https://vimeo.com/1188593847?fl=pl&fe=sh

I hope this helps to improve your manuscript a bit!

References

Abernathey, R., & Haller, G. (2018). Transport by Lagrangian vortices in the eastern Pacific. Journal of Physical Oceanography, 48(3), 667-685.

Abernathey, R. P., & Marshall, J. (2013). Global surface eddy diffusivities derived from satellite altimetry. Journal of Geophysical Research: Oceans, 118(2), 901-916.

Chinn, B. S., & Gille, S. T. (2007). Estimating eddy heat flux from float data in the North Atlantic: The impact of temporal sampling interval. Journal of Atmospheric and Oceanic Technology, 24(5), 923-934.

de Szoeke, R. A., & Levine, M. D. (1981). The advective flux of heat by mean geostrophic motions in the Southern Ocean. Deep Sea Research Part A. Oceanographic Research Papers, 28(10), 1057-1085.

Ferrari, R., & Nikurashin, M. (2010). Suppression of eddy diffusivity across jets in the Southern Ocean. Journal of Physical Oceanography, 40(7), 1501-1519.

Foppert, A., Donohue, K. A., Watts, D. R., & Tracey, K. L. (2017). Eddy heat flux across the Antarctic Circumpolar Current estimated from sea surface height standard deviation. Journal of Geophysical Research: Oceans, 122(8), 6947-6964.

Frenger, I., Münnich, M., Gruber, N., & Knutti, R. (2015). Southern Ocean eddy phenomenology. Journal of Geophysical Research: Oceans, 120(11), 7413-7449.

Gille, S. T. (2003). Float observations of the Southern Ocean. part II: Eddy fluxes. Journal of Physical Oceanography, 33(6), 1182-1196.

Hogg, A. M. (2010). An Antarctic Circumpolar Current driven by surface buoyancy forcing. Geophysical Research Letters, 37(23).

Johnson, G. C., & Bryden, H. L. (1989). On the size of the Antarctic Circumpolar Current. Deep Sea Research Part A. Oceanographic Research Papers, 36(1), 39-53.

Klocker, A., Ferrari, R., & LaCasce, J. H. (2012a). Estimating suppression of eddy mixing by mean flows. Journal of Physical Oceanography, 42(9), 1566-1576.

Klocker, A., Ferrari, R., LaCasce, J. H. & Merrifield, S. T. (2012b). Reconciling float-based and tracer-based estimates of lateral diffusivities. Journal of Marine Research, 70(4), 509-534.

Klocker, A., Munday, D. R., & Gayen, B. (2025). Aspects of the Global Thermohaline Circulation in the Absence of Wind Forcing. Tellus A: Dynamic Meteorology and Oceanography, 77(1), 221-239.

Liu, T., Qian, Y. K., Liu, X., Peng, S., & Chen, D. (2024). Irreversible mixing induced by geostrophic turbulence over the global ocean. Journal of Physical Oceanography, 54(3), 911-928.

Morrison, A. K., Waugh, D. W., Hogg, A. M., Jones, D. C., & Abernathey, R. P. (2022). Ventilation of the Southern Ocean pycnocline. Annual Review of Marine Science, 14, 405-430.

Riser, S. C., Freeland, H. J., Roemmich, D., Wijffels, S., Troisi, A., Belbéoch, M., ... & Jayne, S. R. (2016). Fifteen years of ocean observations with the global Argo array. Nature Climate Change, 6(2), 145-153.

Volkov, D. L., Fu, L. L., & Lee, T. (2010). Mechanisms of the meridional heat transport in the Southern Ocean. Ocean Dynamics, 60(4), 791-801.

Responses to the reviewers

We would like to thank the three anonymous reviewers for their constructive comments on our manuscript, which have significantly helped us to improve the quality of this work. Based on these valuable suggestions, we have performed a comprehensive revision of the manuscript.

We provide a detailed, point-by-point response to each reviewer's comment below, with the reviewers' comments shown in blue and our responses in black.

Reviewer #1

I would like to thank the authors for their careful and complete resubmission. They have significantly improved this manuscript, and I believe it is just about ready for publication after a few minor edits.

We thank the reviewer for the positive assessment of our revised manuscript and for the constructive suggestions. We have carefully addressed the remaining comments and made the corresponding revisions, as described below.

R1-1 Paragraph starting at L116: After removing the eddy trapping/stirring section, this paragraph/analysis feels a bit out of place and confusing. Consider removing from here and the methods section (L390).

We thank the reviewer for this suggestion. We prefer to retain this analysis because it provides useful context for interpreting the difference between B-SOSE and the observations. In particular, the comparison indicates that B-SOSE underestimates the strength of mesoscale eddies relative to the observations, which helps explain why the model does not fully reproduce the magnitude of the observed deep EMHT. Therefore, we believe this analysis remains relevant even after the removal of the eddy trapping/stirring section.

R1-2 L169: I feel like this sentence needs a bit more. Explain what the theoretical framework of eddy mixing suppression is.

We thank the reviewer for this helpful suggestion. We have revised the sentence in Lines 172-174.

"This vertical structure provides observational support for the theoretical framework of eddy mixing suppression across strong jets[24,26,38], in which strong mean flows such as the ACC suppress cross-jet eddy mixing near the surface, while this suppression weakens at depth as mean flows slow."

R1-3 L176: I thought the SOSE validation was a great addition, nice job!

We thank the reviewer for the positive feedback. This validation analysis was motivated by the reviewer's previous suggestion, and we appreciate the helpful guidance.

R1-4 Fig 3: can the colorbar be moved off the figure, like in figure 4? Also I assume the values are negative because these are exponents? Maybe just make that clear in the colorbar label. Same goes for colorbar on fig 4.

Revised as suggested. We have moved the Fig. 3 colorbar outside the plotting panels and revised the Fig. 3 caption to clarify that the colorbar values are shown on a log10 scale.

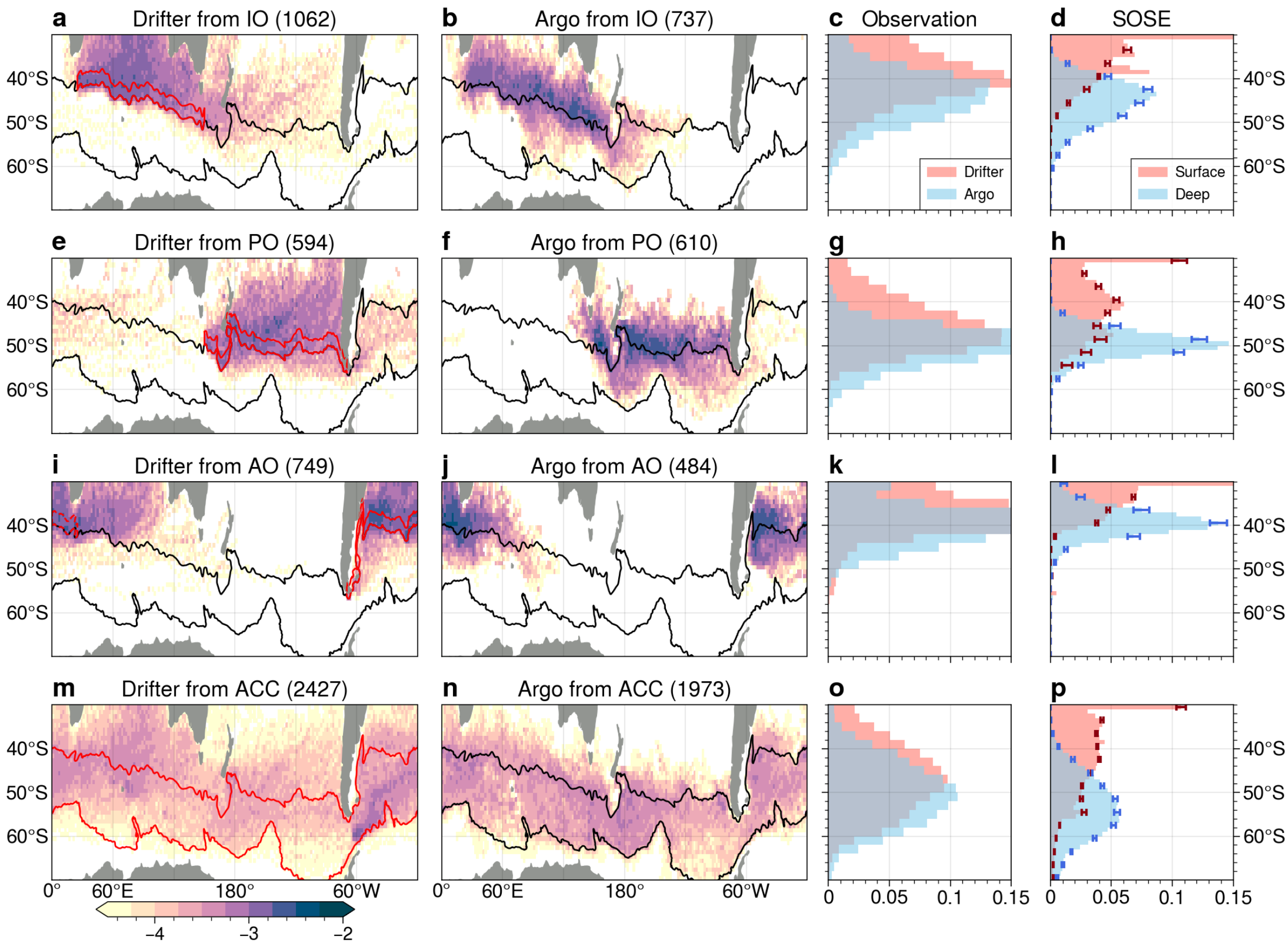

Fig. 3 | Spatial and latitudinal probability distributions of surface drifters, Argo floats, and B-SOSE **particles in the Southern Ocean. a–d**, Spatial probability distributions of surface drifters (**a**) and Argo floats (**b**) from the Indian Ocean sector over 1000 days, along with the latitudinal probability distributions of the observations (**c**) and B-SOSE particles (**d**). **e–h**, from the Pacific Ocean sector. **i–l**, from the Atlantic Ocean sector. **m–p**, from the entire ACC region. Numbers in parentheses indicate total counts for each region. Red lines in the first column outline the initial positions of all observations and model particles. Color shading indicates log10 probability density. Black lines

mark the northern and southern boundaries of the ACC. Error bars in the fourth column represent the standard deviation of 50 random subsamples.

R1-5 L271: I know what you mean when you say ‘without external replenishment’ but this phrasing is a bit weird. That water moving southward obviously has to be replaced somehow. Is it upwelling? Is it possible that cooler waters are being moved northward?

We thank the reviewer for pointing this out. We agree that the phrase “without external replenishment” was unclear. We have revised the sentence to clarify that this result comes from a 2D offline tracer experiment, in which the tracer field is advected horizontally and is not restored or externally replenished. Lines 274-278.

“As temperature tracers evolve, relatively warm tracer north of the ACC is advected southward into the ACC region. Because the tracer field is not restored or externally replenished in a 2D offline experiment, negative anomalies emerge north of the ACC. Meanwhile, the ACC region exhibits significant warming, particularly in the EKE hotspots where positive temperature anomalies exceed 1.5°C.”

R1-6 L273: I’m not exactly sure which part of the analysis you are pointing to when you say ‘preceding Argo trajectory analysis further…’ sentence is just a bit confusing. Revise.

We thank the reviewer for pointing this out. We have revised the sentence to specify the relevant analyses more clearly. Lines 279-280.

“Building on previous studies[11,19,36], the Argo float distributions shown in Fig. 3 and Supplementary Fig. 7 indicate that these deep warm intrusions originate primarily from the Indian Ocean sector.”

R1-7 L297: I find this sentence a bit misleading, you have shown that mean EMHT at depth is about half that at the surface. You noted that at depth, it can reach values of that at the surface but I took that to mean instantaneous deep EMHT can reach values of mean EMHT at surface.

We apologize that the original wording was unclear. As clarified in our response to the next comment, our diagnosis is based on more than 550,000 individual Argo velocity samples and provides a climatological estimate of deep EMHT in the Southern Ocean. The EMHT values shown in Fig. 2 are climatological estimates, not instantaneous values. We find that the zonally integrated EMHT at 1000 m is about half of the surface value. In addition, the B-SOSE validation indicates that the particle-based estimate tends to underestimate the gridded EMHT magnitude,

suggesting that the Argo-based estimate is likely conservative. We therefore consider it reasonable to conclude that deep EMHT reaches the same order of magnitude as the surface transport. To avoid further ambiguity, we have revised the sentence to explicitly include the climatological nature of the estimate and the approximately half-surface magnitude. Lines 305-307.

"We find that climatological EMHT in the deep Southern Ocean reaches at least the same order of magnitude as the surface transport, with its zonally averaged value at 1000 m reaching about half of the surface value, even though deep EKE is only one-fifth of surface levels."

R1-8 Section starting at L403: I'm sorry to harp on this, I think I understand but I just want you to be explicitly clear on how you arrived at your values of u' and v'. Do you get them directly from the Argo Trajectory based velocity product? Meaning, does this product have a time dimension? I was under the impression that the Argo product produced a time mean velocity field, but I could absolutely be wrong about that. If not, I assume the prime quantities were calculated directly by you from individual trajectories?

We thank the reviewer for raising this point. To answer the question directly, u' and v' are not obtained directly from the Argo product, but are calculated from the individual velocity estimates provided by the product.

The Argo trajectory-based velocity product provides individual float-cycle velocity estimates at the parking depth (~1000 m), with associated positions and times, rather than a time-mean gridded velocity field. In our analysis, we use more than 500,000 individual velocity samples from this product (see Lines 354-377).

Because the Argo-derived velocities are based on approximately 10-day displacements, they cannot resolve motions with periods shorter than 10 days. We therefore use B-SOSE to assess the importance of these unresolved high-frequency motions and show that they are negligible at 1000 m. On this basis, the observed Argo velocities could be treated as instantaneous velocities for estimating mesoscale perturbations. We calculate the local mean velocity within each 2° × 1° grid cell from all individual Argo samples and define the anomalies as $u' = u - \bar{u}$ and $v' = v - \bar{v}$. Similar approaches have been applied to earlier sparse float observations by Gille (2003) and Chinn and Gille (2007). We also validate this procedure using B-SOSE fields and show that the same particle-based sampling method can reproduce the climatological deep EKE and EMHT. Lines 431-452.

Gille, S. T. (2003). Float observations of the Southern Ocean. part II: Eddy fluxes. Journal of physical oceanography, 33(6), 1182-1196.

Chinn, B. S., & Gille, S. T. (2007). Estimating eddy heat flux from float data in the North Atlantic: The impact of temporal sampling interval. Journal of Atmospheric and Oceanic Technology, 24(5), 923-934.

R1-9 L433: remove 'at' at the end of this sentence I think?

Revised as suggested. Line 458.

R1-10 The table at the end is helpful, thank you. Nice edits!

We thank the reviewer for the positive and useful feedback.

Reviewer #2

In this revised manuscript, the authors have made a significant effort to address reviewer comments. I appreciate their efforts to provide greater clarity and better citation of previous work in this area. I continue to have several concerns that need to be addressed. At a basic level, the authors have not met fundamental expectations for reproducible science by providing uncertainty estimates and by documenting known challenges associated with the data used for the study.

We thank the reviewer for acknowledging the efforts made in our previous revision. We also appreciate the reviewer's important comments regarding uncertainty estimates and data limitations. Following this suggestion, we have further revised the manuscript to improve uncertainty quantification and to more clearly document the limitations of the datasets used in this study. The specific changes made in response to each comment are described in detail below.

R2-1 Line 89 and following. Resolution and high-resolution performance of the Biogeochemical version of SOSE. In the results discussion of Biogeochemical-SOSE, it would be useful to indicate the resolution of the version being used here. (Note that by convention, the developers of the BGC version of SOSE refer to it as "B-SOSE".) More importantly, in the methods section, there seem to be gaps in the information about the fidelity of the state estimate fields at high wavenumber. Is the spectral slope realistic for scales shorter than 50 km? Are eddies realistic? Supplementary Fig. 2 shows spectra from SOSE, but it would be useful to compare high-wavenumber and/or high-frequency variability in the model with mooring data or with an equivalent assessment of a higher-resolution forward

model (such as llc4320), or other independent information, to back up the assertion that motions with scales less than 10 days account for only about 1% of kinetic energy. The drop-off in energy in Supplementary Fig. 2 for temporal scales shorter than about 25 days is consistent with behavior that might result from the model's suppression of energy at scales shorter than about 4 times the grid spacing. This could suggest that the model behavior is governed by imposed diffusivity, which could have distinctly different physics than the eddies in the ocean.

We thank the reviewer for this helpful suggestion. We have revised the manuscript to use the conventional abbreviation "B-SOSE" throughout and added the horizontal resolution of the B-SOSE version used in this study at its first mention.

We agree that the high-frequency behavior of B-SOSE should be evaluated using independent information. Following the reviewer's suggestion, we repeat the same temporal spectral analysis using the 1/12° GLORYS reanalysis over the same period as B-SOSE, 2013–2017 (see Respones Fig. 1). The GLORYS result shows that motions with periods shorter than 10 days account for only 2.3% of the total kinetic energy at 1000 m, consistent with the B-SOSE estimate of 1.2%.

We further examine four publicly available deep mooring records from three characteristic Southern Ocean regions, including the Agulhas Retroflection, the open Southern Ocean southwest of Tasmania, and Drake Passage (Supplementary Table 1). These records comprise WHOI 835 (40.133°S, 16.576°E, 958 m, February 1985–February 1987), SAZ47-2010 (46.830°S, 141.650°E, 1100 m, September 2010–August 2011), SAZ47-2015 (46.830°S, 141.650°E, 1100 m, March 2015–March 2016), and Drake Passage M7 (58.958°S, 58.096°W, 980 m, January 2006–December 2007). The first three records are sampled at 30-min intervals. We apply a 1-day running mean before the spectral analysis to suppress tidal and near-inertial motions, which are expected to make only a small net contribution to the eddy heat transport considered here. The M7 record is provided as daily output after 2-day smoothing. Motions with periods shorter than 10 days account for approximately 7%, 5%, 3%, and 2% of the total kinetic energy in the four records, respectively. Although these fractions are somewhat higher than the B-SOSE estimate, they remain below 8%. This suggests that B-SOSE likely underestimates sub-10-day kinetic energy, but does not alter our conclusion that the kinetic energy near 1000 m relevant to our eddy-transport analysis is dominated by low-frequency variability with periods longer than 10 days.

We agree that comparison with an ultra-high-resolution forward model such as LLC4320 would also be useful. However, LLC4320 has an extremely large data volume and is difficult for us to access and process for this analysis. We therefore use GLORYS and the mooring observations as independent constraints on the B-SOSE-based spectral

result. Because the GLORYS spectrum is very similar to B-SOSE, we report the GLORYS result in this response, and the revised Supplementary Fig. 2 shows the comparison between B-SOSE and the mooring observations. We have also revised the relevant text in the main manuscript. Lines 341-344 and 440-447.

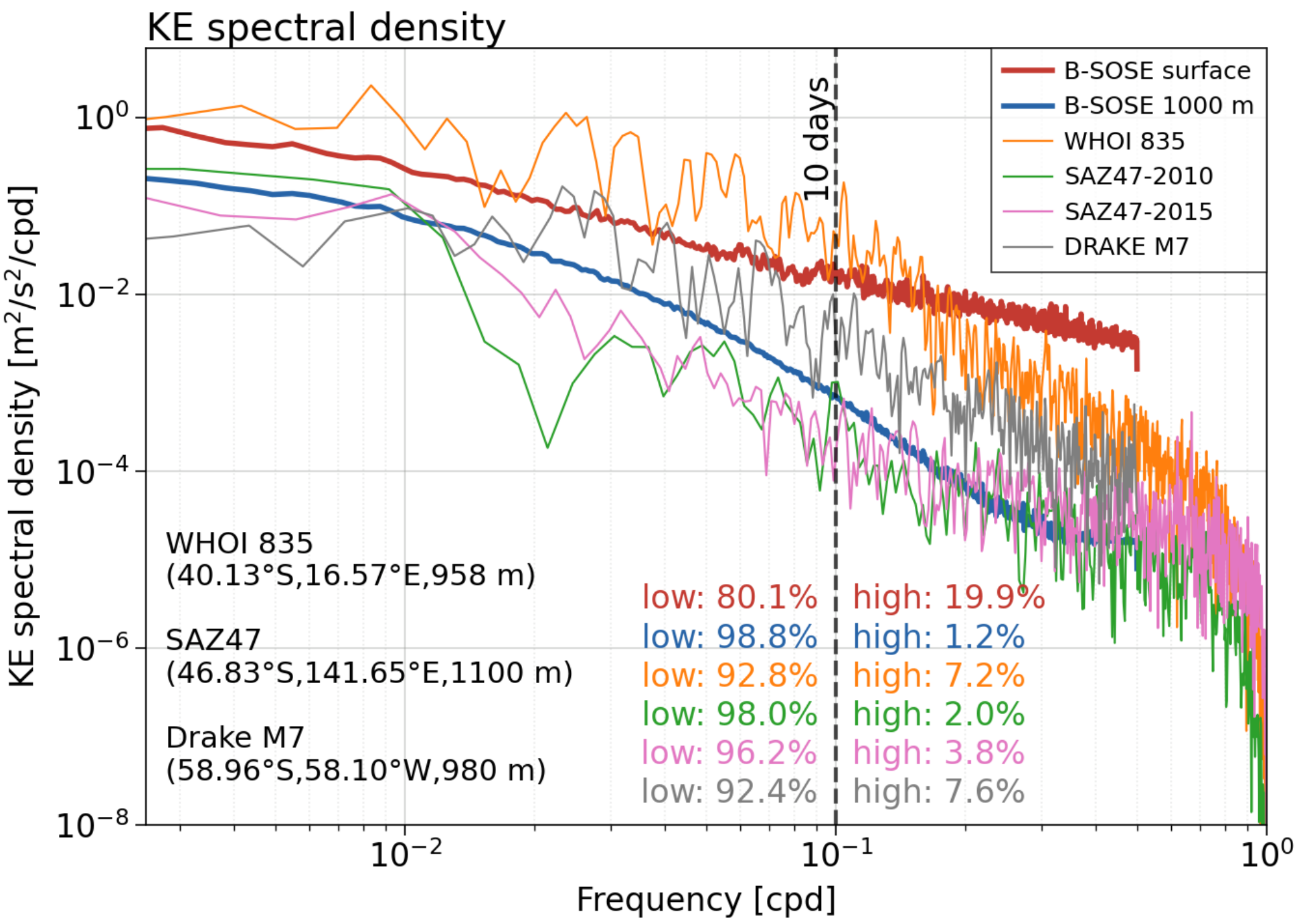

Supplementary Fig. 2 | Temporal spectra of kinetic energy from B-SOSE and deep mooring observations. Temporal power spectra of kinetic energy derived from spatially averaged B-SOSE velocity fields over the Southern Ocean at the surface (red) and 1000 m (blue). The four mooring spectra are from WHOI 835 (40.13°S, 16.57°E, 958 m; orange), SAZ47-2010 (46.83°S, 141.65°E, 1100 m; green), SAZ47-2015 (46.83°S, 141.65°E, 1100 m; pink), and Drake M7 (58.96°S, 58.10°W, 980 m; grey). The WHOI 835, SAZ47-2010 and SAZ47-2015 records have 30 min sampling and are smoothed in this study with a 1-day running mean. The Drake M7 record is distributed as daily output with 2-day smoothing. The dashed vertical line marks a period of 10 days. Percentages indicate the fractions of kinetic energy contained in low-frequency (>10 days) and high-frequency (<10 days) motions.

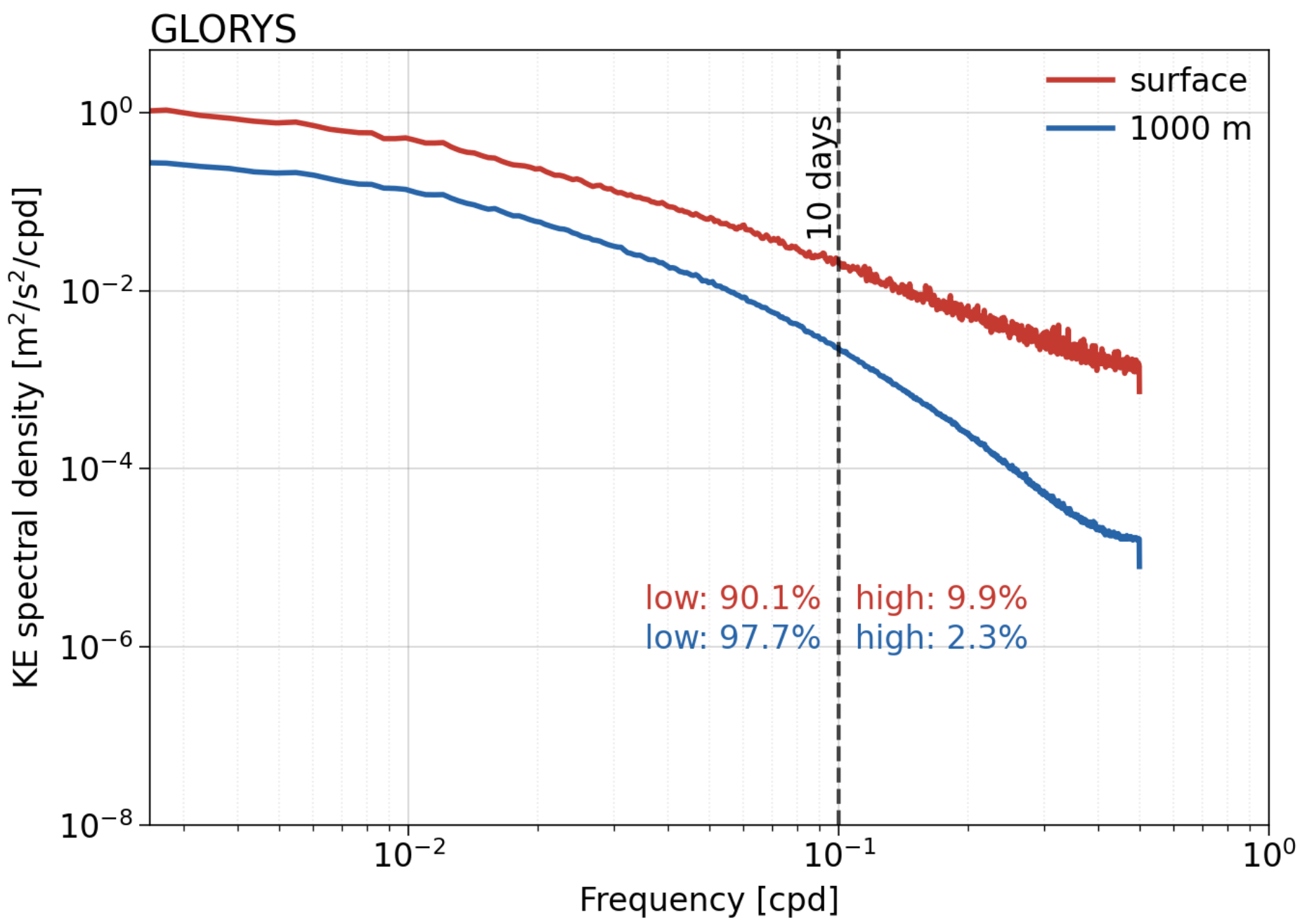

Response Fig. 1 | Temporal spectra of kinetic energy from GLORYS dataset.

R2-2 Figure 2 caption. I appreciate the inclusion of shaded uncertainty ranges in panel b, but I do not understand why the surface values do not include an uncertainty range. The surface values should be reported with an uncertainty.

We thank the reviewer for this helpful comment. We agree that the uncertainty information in Fig. 2b should be clearly described. The shaded range in Fig. 2b represents the bootstrap sampling uncertainty of the Argo-derived 1000 m EMHT estimate, which arises from the sparse sampling of float observations. In contrast, the surface EMHT shown by the black line is calculated from gridded satellite-based surface fields and is included as a reference estimate for comparison. Its uncertainty would involve different sources, such as gridded-product and mapping errors as well as temporal or spatial variability, rather than the Argo sampling uncertainty quantified by our bootstrap analysis. To avoid mixing non-equivalent uncertainty measures, we did not include a directly comparable uncertainty envelope for the surface estimate in the previous version. Following the reviewer's suggestion, we have now added a grey shaded range for the surface EMHT, representing the standard deviation from the monthly climatology, and clarified this in the Fig. 2 caption. Lines 160-161.

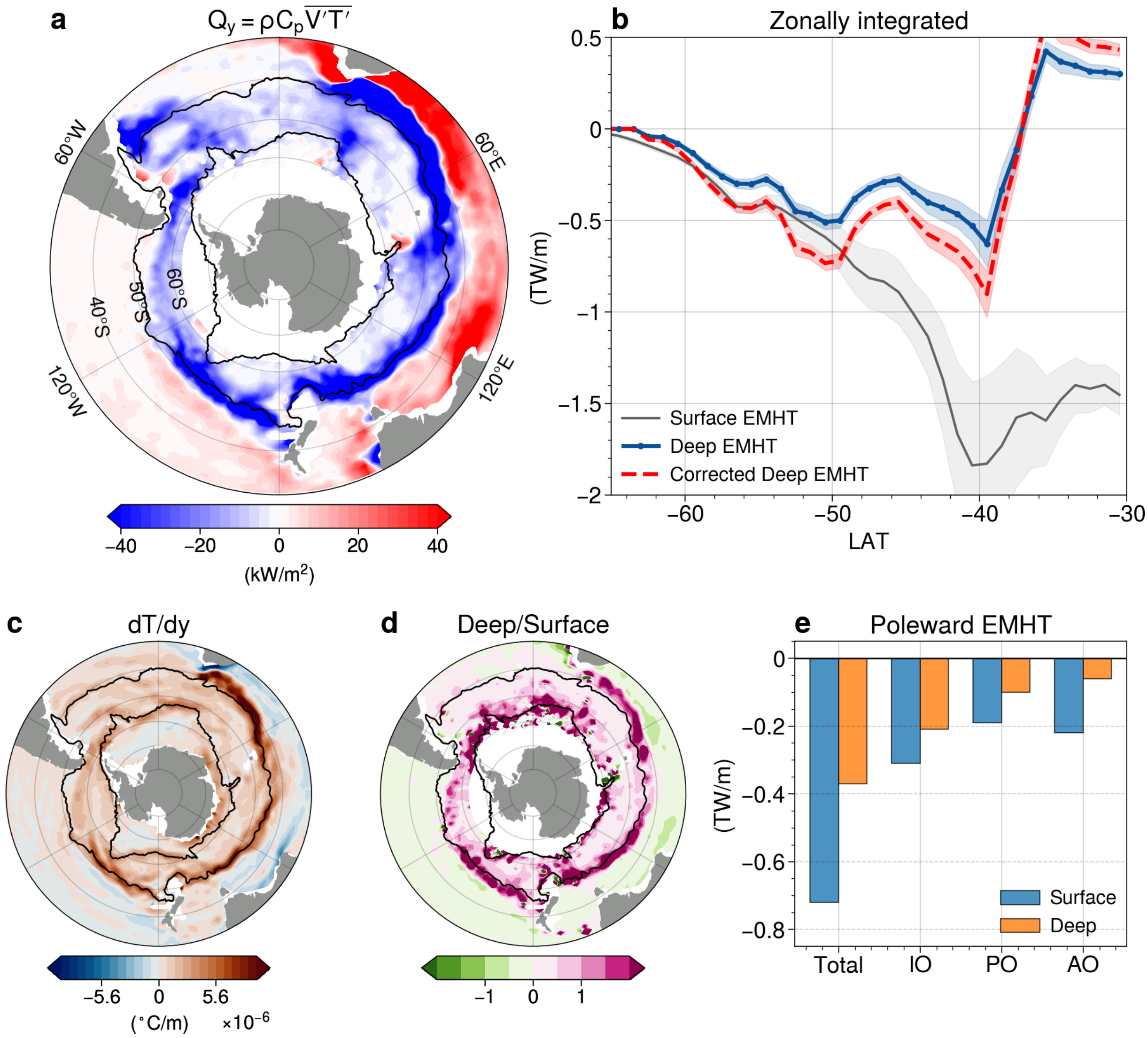

Fig. 2 | EMHT in the deep Southern Ocean. a, Spatial distribution of EMHT at 1000 m depth (color shading; kW/m²). Black contours mark the northern and southern boundaries of the ACC. **b**, Zonally integrated EMHT at 1000 m depth versus latitude (blue line; TW/m). Shading denotes twice the standard deviation from 1000 bootstrap resamples. The red line shows the bias-corrected estimate. The black line shows surface EMHT from gridded satellite-based fields for comparison, with grey shading indicating the standard deviation from monthly climatology. **c,** Spatial distribution of the meridional temperature gradient at 1000 m. **d,** Ratio of EMHT at 1000 m depth to surface EMHT. **e,** Basin-scale comparison of poleward EMHT at 1000 m and at the surface.

R2-3 Line 163. The maximum and mean EMHT values should be reported with uncertainty estimates. It's worth noting that the mean surface transport of -0.71 TW/m represents a mean of a highly variable quantity. Typically, readers expect mean quantities to be reported along with a standard error of the mean.

We thank the reviewer for this suggestion. We have added uncertainty estimates for the Argo-derived deep EMHT values reported in the text, including both the maximum poleward flux and the mean transport between 40°S and 60°S. These uncertainties are estimated from 1000 bootstrap resamples and are now reported as standard deviations. Lines 165, 166-167, and 180.

R2-4 Figure 3 shows trajectories from Argo floats over 1000 days. As my previous review mentioned, Argo trajectories are not meaningful representations of 1000-day motions because the floats move much faster near the surface than they do at 1000 m. The reply to reviewers indicates that the authors have not fully grasped this point. They write, "Once a drifter or float first enters the prescribed source region, we follow its subsequent trajectory for up to 1,000 days, if such records are available." Let me be clear about this. In order to transmit data, the older pre-Iridium floats spent as much as 24 hours at the surface during each 10-day cycle. An estimated 50% of net displacement occurred at the surface. Thus the 1000-day trajectories are as much an indicator of surface motion as 1000-m motion. Iridium floats avoid this challenge for the most part, but they still are imperfect Lagrangian particles, and their trajectories should be interpreted with caution. Caveats about the trajectories appear to be missing from the manuscript.

We thank the reviewer for clarifying this important point. We have revised the Argo data description to provide more background on the surface-drift issue and on the velocity product used in this study. Specifically, we now note that traditional Argo floats may remain at the sea surface for 6–18 hours during data transmission, whereas modern Iridium Argo floats typically require less than 20 minutes, following Wong et al. (2020). We also clarify that the Scripps Argo trajectory-based velocity product estimates parking-depth velocities using extrapolated descent and arrival positions, which reduces contamination from surface drift. In our analyzed dataset, corrected traditional Argos-transmitting records and Iridium Argo observations together account for 97.8% of the samples used for the velocity analysis.

We agree that this correction for velocity estimates does not make the 1000-day float trajectories ideal Lagrangian trajectories at 1000 m. We have therefore added an explicit caveat in the Trajectory analysis section. We state that Argo floats are profiling instruments rather than ideal Lagrangian particles, and that 1000-day Argo float trajectories should not be interpreted as exact trajectories of water parcels at 1000 m. This limitation is particularly relevant for older Argos-transmitting Argo floats, for which surface drift during data transmission may introduce additional uncertainty into the total displacement. Accordingly, we interpret the Argo-based distributions in Fig. 3 as

broad float displacement patterns rather than precise Lagrangian pathways of individual deep water parcels. Lines 362-363, 373-377, and 482-487.

Wong, A. P., Wijffels, S. E., Riser, S. C., Pouliquen, S., Hosoda, S., Roemmich, D., ... & Park, H. M. (2020). Argo data 1999–2019: Two million temperature-salinity profiles and subsurface velocity observations from a global array of profiling floats. Frontiers in Marine Science, 7, 700.

R2-5 With reference to my recommendation to add error bars to Figure 4, the authors wrote in their response document, "For a given velocity field and initial tracer concentration, the tracer distribution is deterministic. Therefore, statistical uncertainty or statistical significance is not directly defined for this figure." This comment suggests that the authors have not fully thought out the nature of a random variable. Although they have only one realization of the model, we can hypothesize the possibility of an ensemble of realizations, or an ensemble of particle release experiments, all with slightly different values. Uncertainty estimates are important as a means for assessing robustness for these values. Small perturbations in release data and small perturbations in velocity would imply differences in distribution that should be assessed.

We thank the reviewer for pointing this out. We agree that our previous response does not adequately address the need to assess the robustness of the tracer diagnostics. Although each offline tracer simulation is deterministic for a prescribed velocity field and initial tracer distribution, the results can still be sensitive to the release timing and to the phase of the resolved velocity field. Following the reviewer's suggestion, we perform four additional sensitivity experiments in which the velocity fields are initialized 90, 180, 270, and 360 days after the original start date of 1 January 2013, with each experiment integrated for 1000 days. These experiments provide a small ensemble for evaluating the sensitivity of the tracer distribution and related diagnostics to perturbations in the advecting flow.

We considered adding the resulting standard deviations directly to Fig. 4, but the ensemble spread is very small and is difficult to distinguish in the main figure. For example, the standard deviation of the meridional extent for the Indian, Pacific, and Atlantic tracer releases is less than 0.2°, and the remaining extent diagnostics show negligible sensitivity to the release-time perturbations. The time-dependent standard deviation normalized by the ensemble mean tracer amount entering the ACC is generally less than 2% after 400 days, with the largest values occurring only during the initial adjustment period. We therefore add Supplementary Fig. 10 to summarize these uncertainty

estimates and revise the corresponding text in the manuscript accordingly. These results indicate that the tracer diagnostics are robust to the tested release-time perturbations. Lines 523-527.

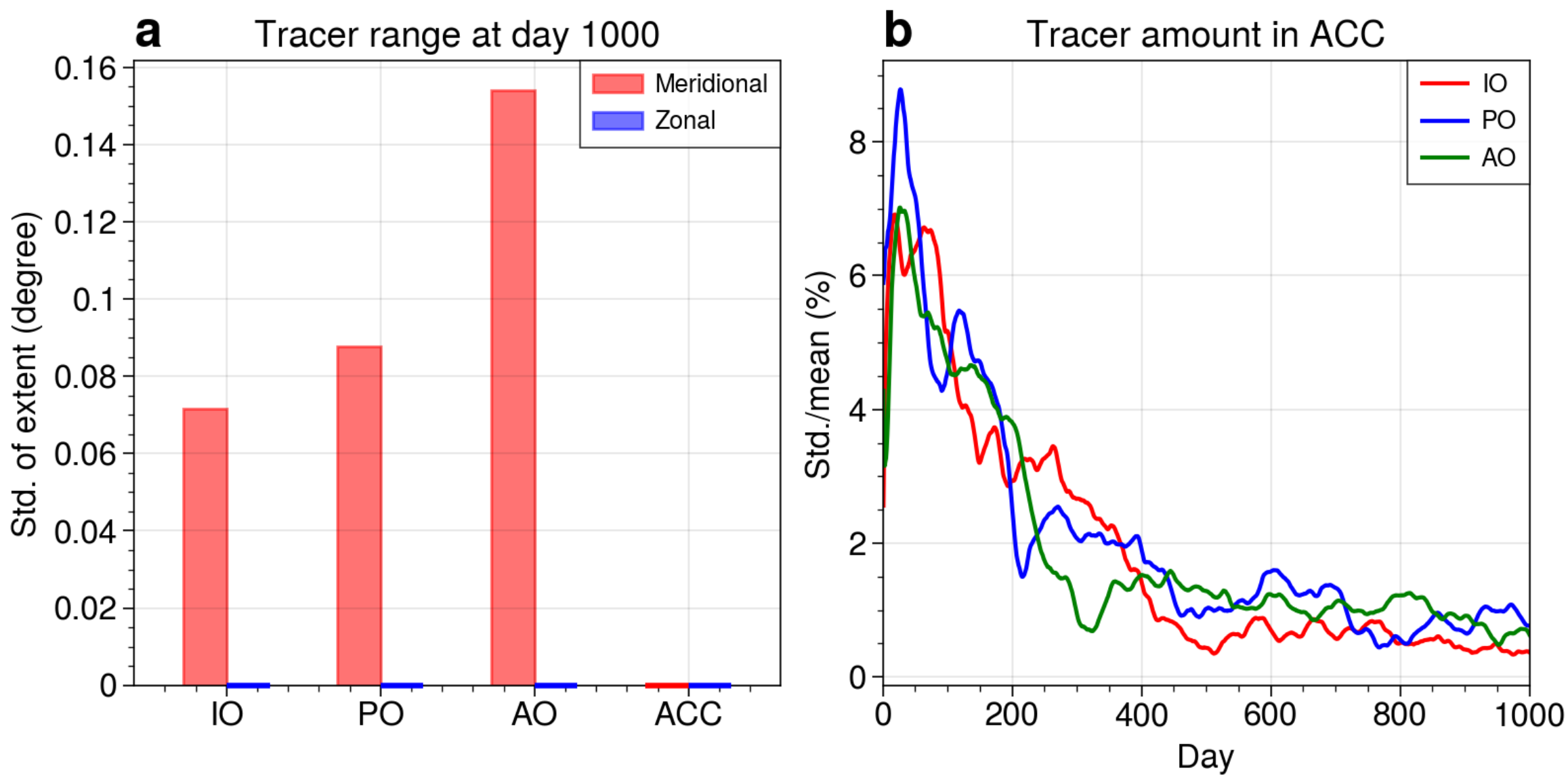

Supplementary Fig. 10 | Sensitivity of tracer diagnostics to release-time perturbations. **a**, Standard deviation of the meridional and zonal extents of tracer distributions at day 1000 across the five eddy-resolving experiments. **b**, Time-dependent ratio of the standard deviation to the ensemble mean of cumulative tracer amount entering the ACC, calculated across the five experiments. The five experiments use velocity fields initialized on 1 January 2013 and 90, 180, 270, and 360 days later.

R2-6 Experiments 5 and 6 are compared in Figure 5. Notably, Fig 5c reports heat flux in units of J/m as function of time. It's not clear if the average temperature gradient is the same in Experiments 5 and 6. Since the results are reported in units of J/m (rather than as an effective diffusivity), the net heat transport is expected to vary with the magnitude of the temperature gradient. More detail is needed to demonstrate that the plotted results have any useful meaning.

We thank the reviewer for this important comment. Figure 4 shows the spatial dispersion of water masses originating from different ocean basins. However, a uniformly initialized tracer only reflects water-mass dispersion and cannot directly diagnose heat transport. Therefore, in Fig. 5, we use a tracer field initialized with the climatological temperature distribution to diagnose EMHT. Figure 5c shows the temporal evolution of the accumulated heat-content difference between the eddy-resolving and mean-flow simulations, which represents the cumulative net heat transport induced by eddies. The calculation is performed on a two-dimensional horizontal plane

at 1,000 m and is expressed per unit vertical thickness. Therefore, its unit is J m^{-1}. We have revised the figure caption and Methods to clarify the definition and physical interpretation of this quantity.

Our comparison of EX5 and EX6 focuses on changes in the relative contributions of the different ocean basins rather than requiring identical mean temperature gradients. EX5 uses the climatological temperature distribution to estimate EMHT under a realistic background temperature field. The dominant contribution from the Indian Ocean sector is consistent with our preceding analysis showing strong regional temperature gradients in this sector. EX6 is an idealized sensitivity experiment initialized with a tracer field that decreases linearly from north to south, thereby removing the realistic basin-dependent structure of the background temperature field. In EX6, the relative contribution of the Atlantic sector increases from 10% to 46%, whereas that of the Indian Ocean sector decreases from 63% to 46%. These changes demonstrate that differences in EMHT among the ocean basins are strongly influenced by the magnitude and spatial structure of the background temperature gradients. We have revised the corresponding text to clarify the purpose of the experiments and the interpretation of their comparison. Lines 266-267, 285-288, and 290-292.

R2-7 Line 366. The drifters are reported as representing velocities at a nominal depth of 15 m. It would be useful if the methods section clarified whether drifters are excluded when they lose their drogues.

We thank the reviewer for this helpful suggestion. We have clarified in the Methods that we did not exclude drifters after drogue loss. As shown in Supplementary Fig. 7, fewer than 15% of the drifter trajectories last longer than 1000 days. Applying an additional drogue-status filter would have further reduced the already limited sampling. To retain sufficient spatial and temporal coverage, we used all available drifter trajectories. We now explicitly acknowledge that undrogued drifters may be more strongly affected by wind, which could introduce additional uncertainty in the drifter trajectories. However, the surface particle experiments based on B-SOSE show a similar transport pathway (Fig. 3), supporting the robustness of our main interpretation. Lines 383-388.

"It should be noted that we do not exclude drifters after drogue loss in order to retain sufficient spatial and temporal coverage. We acknowledge that undrogued drifters may be affected by wind, which could introduce additional uncertainty in the drifter trajectories. However, the validation analysis based on the B-SOSE surface particle experiments shows pathways similar to the drifter observations (Fig. 3), supporting the robustness of our interpretation."

R2-8 Supplementary Fig. 2. The caption is not clear about the data source for this figure.

We have redrawn Supplementary Fig. 2 to include mooring observations and revised the caption to clearly state the data sources. See our response to R2-1.

R2-9 Supplementary Fig. 3. The caption is not clear about whether this figure is based on observed floats and drifters or on particle release experiments using SOSE fields. The caption should be rewritten for clarity. If SOSE fields are used, then clarity should be provided about differences between SOSE and particle means.

We apologize that the captions were not sufficiently clear. Supplementary Figs. 3 and 5 are both intended to validate the particle-based estimation method using the B-SOSE dataset. We have rewritten both captions to clarify that the gridded B-SOSE estimates are calculated directly from the B-SOSE fields, whereas the particle-based estimates are derived from particles advected by the B-SOSE velocity fields. As described in the main text, the particle-based estimates reproduce the spatial patterns of the gridded B-SOSE estimates well, although the EMHT magnitude is slightly underestimated (Lines 93-98 and 132-137). This validation supports the use of the particle-based approach for the sparse Argo observations. Lines 499-500.

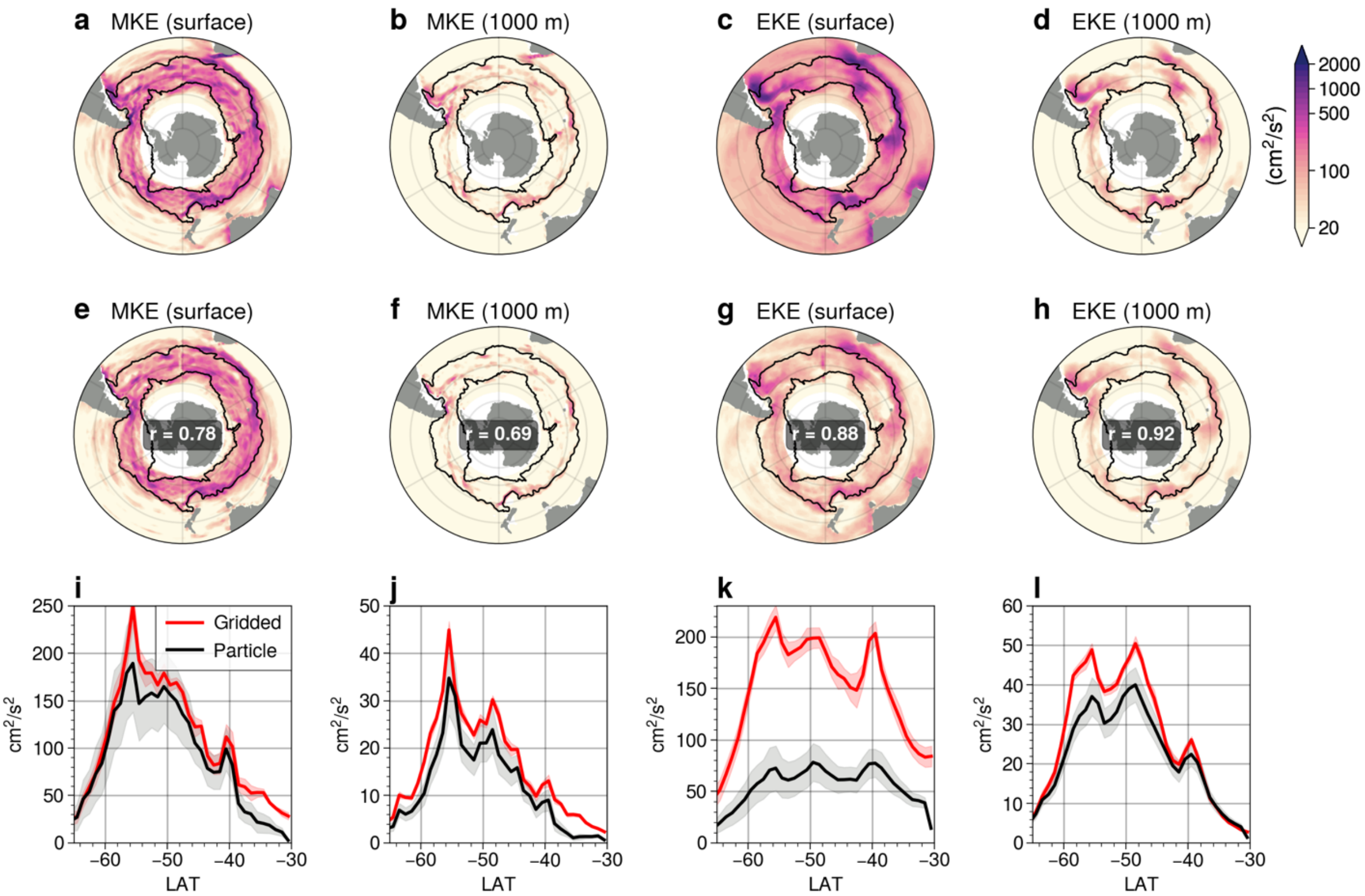

Supplementary Fig. 3 | Validation of particle-based MKE and EKE using B-SOSE dataset. a–d, Gridded B-

SOSE estimates of surface MKE (**a**), MKE at 1000 m (**b**), surface EKE (**c**), and EKE at 1000 m (**d**). **e–h**, As in **a–d**, but for estimates based on particles advected by B-SOSE velocity fields. Numbers in **e–h** indicate the spatial correlation with the corresponding gridded B-SOSE estimates. **i–l**, Zonal-mean comparisons between the gridded B-SOSE estimates and particle-based estimates for surface MKE (**i**), MKE at 1000 m (**j**), surface EKE (**k**), and EKE at 1000 m (**l**). Red lines denote gridded B-SOSE estimates, with shading showing the standard deviation from monthly climatology. Black lines denote particle-based estimates, with shading indicating the standard deviation from 50 random subsamples. Random subsampling is performed to match the sample size of the Argo velocity product used in the analysis.

R2-10 Supplementary Fig. 4. Mean anomalies should be reported with uncertainties representing the standard error of the mean.

We thank the reviewer for this suggestion. We have now reported the mean anomalies with uncertainties calculated as the standard deviation and updated Supplementary Fig. 4 accordingly.

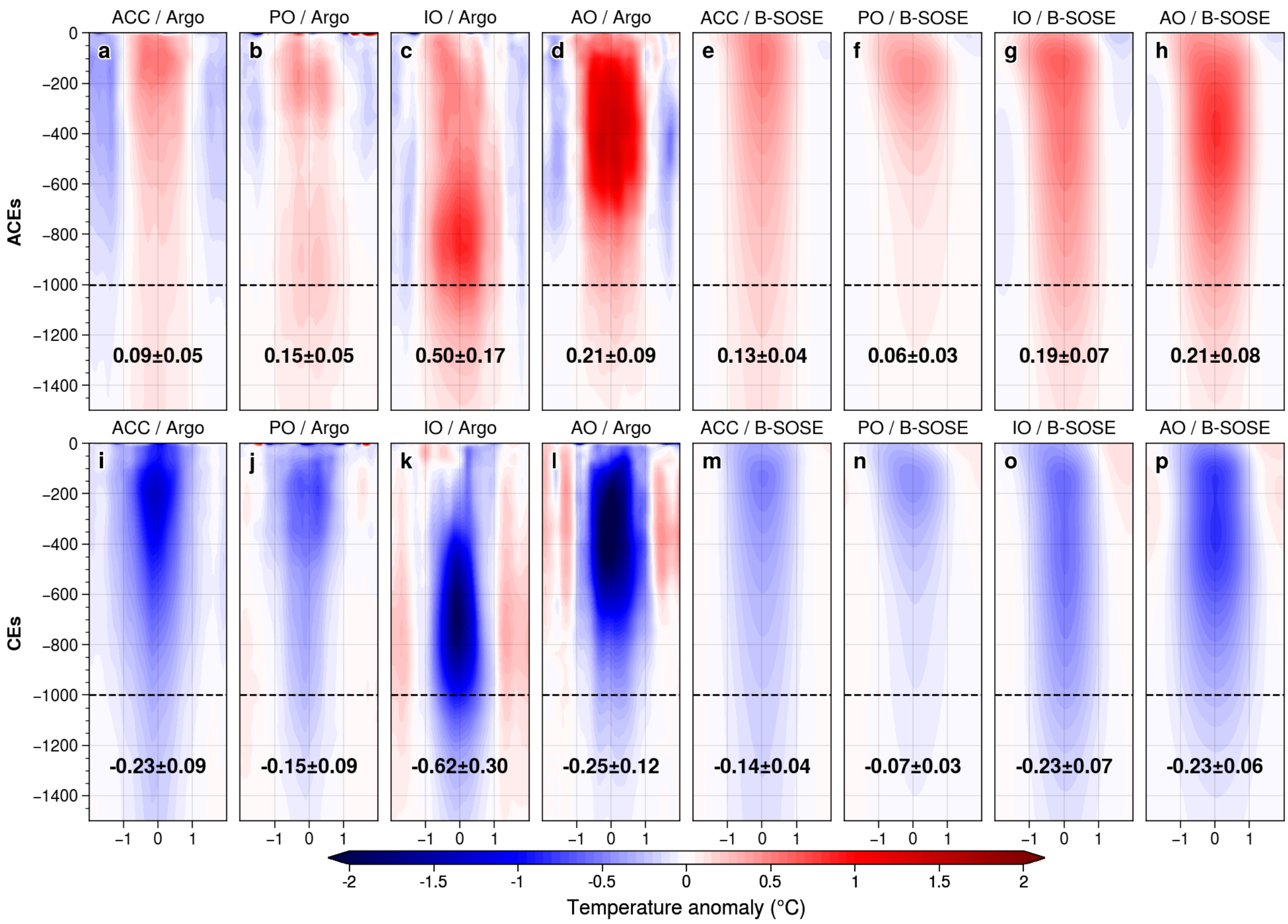

Supplementary Fig. 4 | Composite vertical structure of mesoscale eddy temperature anomalies from Argo and

B-SOSE. a–d, Composite temperature anomalies for anticyclonic eddies derived from Argo in the ACC (**a**), Pacific (**b**), Indian (**c**), and Atlantic (**d**) sectors. **e–h,** Same as **a–d**, but derived from B-SOSE. **i–l,** Composite temperature anomalies for cyclonic eddies derived from Argo in the ACC (**i**), Pacific (**j**), Indian (**k**), and Atlantic (**l**) sectors. **m–p,** Same as **i–l**, but derived from B-SOSE. Numbers in each panel indicate the mean temperature anomaly ± one standard deviation within one normalized eddy radius of the eddy center. The standard deviation is calculated across all individual temperature-anomaly samples. Temperature anomalies are calculated relative to the monthly climatological mean field.

R2-11 Supplementary Fig. 5. The red lines indicating SOSE values are shown without uncertainty estimates. It's not clear why uncertainties are omitted. Does SOSE provide a fully closed budget?

We thank the reviewer for this comment. Similar to our clarification for Fig. 2, the uncertainty shading in Supplementary Fig. 5 represents the uncertainty of the particle-based EMHT estimates, calculated from 50 random subsamples. The SOSE estimates shown by the red lines are computed directly from the gridded SOSE velocity and temperature fields and are used as reference estimates for validating the particle-based method. We do not assume that SOSE provides a perfect or fully closed heat budget. The purpose of Supplementary Fig. 5 is to test whether the particle-based EMHT estimates can reproduce EMHT calculated directly from the same SOSE fields. Consistent with the revisions made to Supplementary Fig. 3, we have added uncertainty shading for gridded B-SOSE estimates and updated the figure caption.

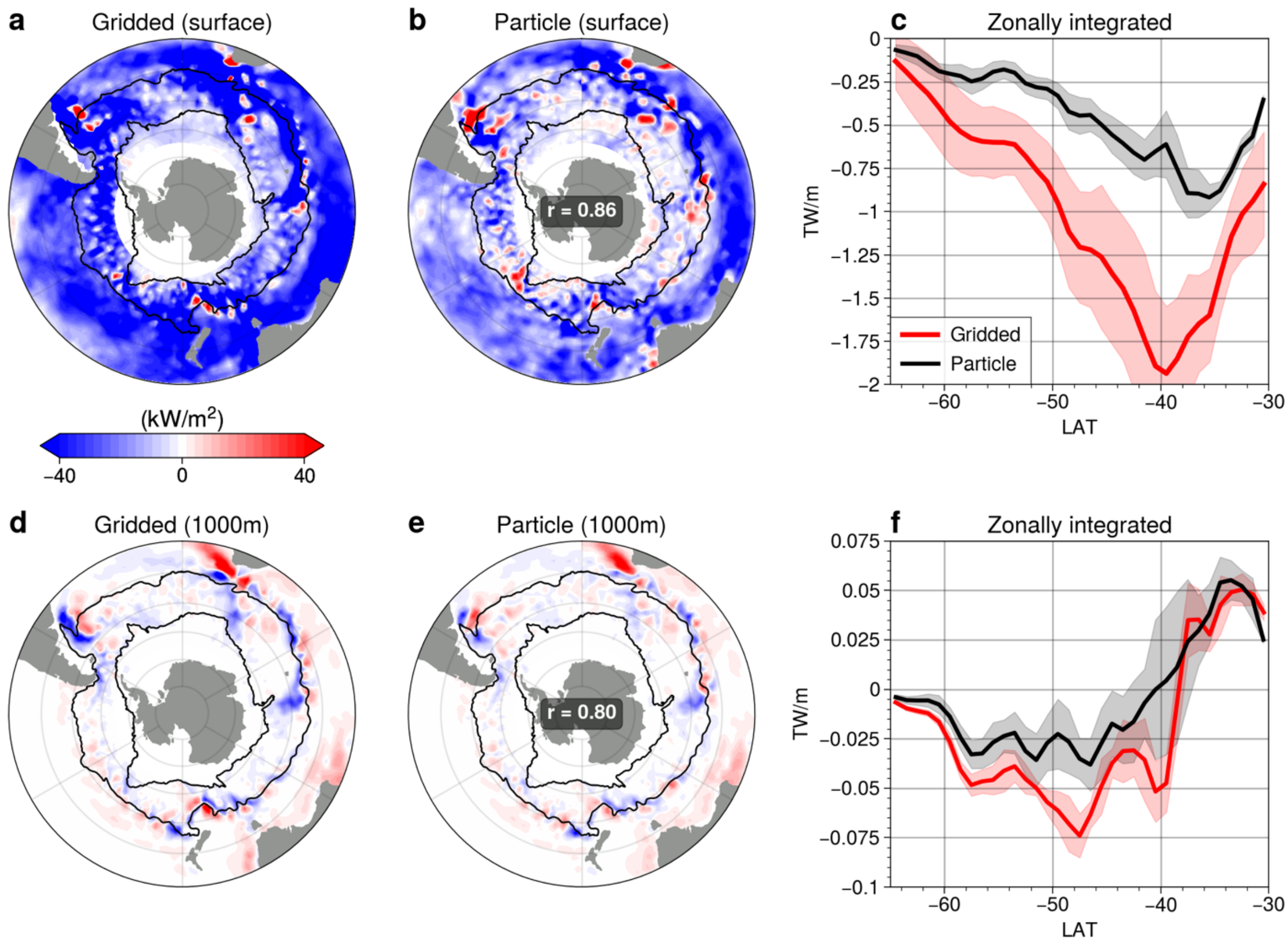

Supplementary Fig. 5 | Validation of particle-based EMHT estimates using the B-SOSE dataset. a,**b**, Surface EMHT estimated from gridded B-SOSE fields (**a**) and from particles advected by B-SOSE fields (**b**). **c**, Zonal-mean comparison of surface EMHT between the gridded B-SOSE and particle-based estimates. **d**,**e**, As in **a**,**b**, but for EMHT at 1000 m. **f**, Zonal-mean comparison of EMHT at 1000 m between the gridded B-SOSE and particle-based estimates. Numbers in **b** and **e** indicate the spatial correlation with the corresponding gridded B-SOSE estimates. In **c** and **f**, red lines denote gridded B-SOSE estimates, with shading showing the standard deviation from monthly climatology, and black lines denote particle-based estimates, with shading indicating the standard deviation from 50 random subsamples. Random subsampling is performed to match the sample size of the Argo velocity product used in the analysis.

R2-12 Supplementary Fig. 5. “grey shading indicates the standard deviation from 50 random subsamples”. This wording is ambiguous. Is the standard deviation dependent on the number of elements included in each subsample? Are the number of samples scaled to match the number of Argo floats used for this analysis?

We thank the reviewer for pointing out that the caption was not sufficiently clear. While the subsampling procedure was described in the Methods (Line 496), we agree that the figure captions should be more self-contained. We have therefore revised the captions of Supplementary Figs. 3 and 5 by adding the following clarification: "Random subsampling is performed to match the sample size of the Argo velocity product used in the analysis."

R2-13 Supplementary Fig. 6. It's important to reiterate that 1000-day Argo trajectories are not meaningful because Argo floats are not designed as Lagrangian particles, and in particular pre-Iridium Argo floats experience a major fraction of their total motion at the surface.

We thank the reviewer for emphasizing this important caveat. Following the response to R2-4, we have added an explicit discussion of this limitation in the Trajectory analysis section (Lines 482-487).

A caveat is that Argo floats are profiling instruments rather than ideal Lagrangian particles. Therefore, 1000-day Argo float trajectories should not be interpreted as exact trajectories of water parcels at 1000 m. This limitation is particularly relevant for pre-Iridium Argo floats, for which surface drift during data transmission may introduce additional uncertainty into the total displacement. We therefore interpret the Argo-based distributions as broad float displacement patterns rather than precise Lagrangian pathways of individual deep water parcels.

R2-14 Grammar and style

Line 44. "deeply (> 1500 m)" would be clearer as "to depths greater than 1500 m"

Revised as suggested. Line 44.

Line 108. "that" -> "their role"

Revised as suggested. Line 110.

Line 131. I would remove "further", since the opening clause of the sentence clarifies the temporal sequence.

Revised as suggested. Line 133.

Line 132. "particle-based estimates". Please clarify that this refers to "SOSE-derived particle-based estimates".

Revised as suggested. Line 134.

Line 452-454. "The number of samples obtained from drifters (Argo floats) in the Indian, Pacific, Atlantic Ocean sectors, and ACC region is 1062 (737), 594 (610), 749 (484), and 2427 (1973), respectively". The use of contrasting examples in parentheses makes the text difficult for readers to interpret. It would be worth putting the information in a table, or else stretching out the sentence to be clearer about the drifter vs Argo examples.

Revised as suggested. We have rewritten the sentence as two separate sentences to clarify the sample numbers for drifters and Argo floats. Lines 477-479.

Reviewer #3

I'm happy with the changes the authors made in response to my comments, and hope to see this nice manuscript published soon!

Thank you very much for your positive evaluation. We greatly appreciate your careful review and valuable suggestions throughout the revision process.
